# Pleistocene Climate Changes and Lineage Diversification of *Sphenarium* Grasshoppers (Orthoptera: Pyrgomorphidae)

**DOI:** 10.1002/ece3.72209

**Published:** 2025-10-10

**Authors:** Salomón Sanabria‐Urbán, Andrés Torres‐Miranda, David A. Prieto‐Torres, Ken Oyama, Raúl Cueva del Castillo

**Affiliations:** ^1^ Laboratorio de Ecología, UBIPRO, Facultad de Estudios Superiores Iztacala Universidad Nacional Autónoma de México (UNAM) México México; ^2^ Escuela Nacional de Estudios Superiores (ENES) Unidad Morelia UNAM Morelia Michoacán México; ^3^ Laboratorio de Biodiversidad y Cambio Global (LABIOCG); Facultad de Estudios Superiores Iztacala Universidad Nacional Autónoma de México Tlalnepantla Estado de México México; ^4^ Laboratorio Nacional SECIHTI de Biología del Cambio Climático Ciudad de México México

**Keywords:** genetic differentiation, mitochondrial introgression, niche divergence, phylogeography, Pleistocene refugia

## Abstract

Pleistocene climatic fluctuations have significantly influenced biotic diversification globally, but their impacts on Neotropical insects remain understudied. Here, we evaluated the predictions of the Glacial and Interglacial Refugia models to investigate the effects of Pleistocene climate changes on four *Sphenarium* species: *S. purpurascens* , *S. rugosum* , *S. variabile* , and *S. zapotecum*. We analyzed phylogeographic patterns using a mitochondrial marker and conducted ecological niche and paleodistribution modeling to evaluate niche conservatism and historical distribution dynamics. We found high haplotype diversity (Hd > 0.87) and significant phylogeographic structure (*N*_ST_–*G*_ST_ = 0.17–0.37) in all species except *S. zapotecum*, with non‐monophyly and limited haplotype sharing among *S. purpurascens* , *S. variabile* , and *S. zapotecum*. A few populations showed consistent changes in population size over time. Niche analyses rejected niche conservatism among species (overlap scores < 0.20; *p ≥* 0.05) and minimal geographic overlap of suitable climatic areas since the Late Pleistocene. Climatic stability areas (i.e., potential refugia) cover less than 20% of the currently known distributions of *S. purpurascens* , *S. zapotecum*, and *S. rugosum* . Our findings suggest that Pleistocene climatic changes drove population divergence in *Sphenarium* grasshoppers; however, the impacts varied among species, and neither of the refugial models fully explained all patterns. The observed genetic differentiation, niche divergence, and demographic trends suggest recent differentiation, local adaptation, and complex evolutionary histories shaped by geography and climate. This study highlights the intricate interplay between environmental changes and evolutionary processes in shaping Neotropical insect diversity.

## Graphical abstract

We investigate the phylogeographic history and paleodistribution patterns of four closely related grasshopper species of the genus *Sphenarium* to test the relative impact of Pleistocene climate fluctuations on their diversification. We found that each species responded differently to these fluctuations, but their divergence, distribution, and demographic shifts closely follow historical climate events. This study also advances ecological and evolutionary insights, hinting that natural selection through niche divergence could also contribute to speciation events in *Sphenarium*.

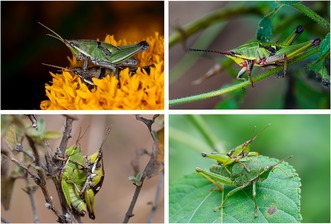

## 1 Introduction

The glacial–interglacial cycles of the Pleistocene (~2.6 to 0.011 million years ago [Mya]) caused repeated fluctuations in temperature and precipitation regimes in Mexico (e.g., Caballero et al. 2010; Lozano‐García et al. 2005; Metcalfe 2006; Sedov et al. 2009; Vázquez‐Selem and Heine 2011), leading to recurrent shifts in the distribution ranges of many species. These climatic fluctuations drove the fragmentation, isolation, and divergence of ancestral populations (e.g., Toledo 1982; Lozano‐García and Ortega‐Guerrero 1998; McAuliffe and Devender 1998; Ortega‐Rosas et al. 2008). Concordantly, both interspecific and intraspecific lineages of multiple taxa in Mexico diverged during the last 2.6 Mya (Aguirre‐Planter et al. 2020; Bessa‐Silva et al. 2020; De‐la‐Mora et al. 2018; Nolasco‐Soto et al. 2017; Osuna et al. 2020; Rodríguez‐Gómez et al. 2021). Temporal correlation between lineage divergences and the glacial–interglacial cycles does not necessarily indicate a causal relationship (Knowles 2001). Therefore, to infer whether glacial–interglacial cycles were likely drivers of divergence, we must test if the observed demographic and genetic patterns correspond with the expected consequences of these climatic events (Knowles 2001).

Two mutually exclusive models have been proposed to explain the effects of the Pleistocene climatic changes on species distribution, historical demography, and lineage diversification in Mexican taxa: the Glacial Refugia versus Interglacial Refugia models (Cornejo‐Romero et al. 2017; Mastretta‐Yanes et al. 2015; Ornelas et al. 2018). The Glacial Refugia model, originally developed from patterns observed in temperate taxa of the Northern Hemisphere (e.g., Soltis et al. 2006), postulates that populations contracted into lowland refugia during cold glacial periods, such as the Last Glacial Maximum (~22 to 18 thousand years ago [kya]), leading to isolation, reduced genetic diversity, and increased phylogeographic structure (Godfrey M. Hewitt 2001; Knowles 2001). Subsequent warming during interglacial periods, including the Holocene (~14 kya–present), facilitated demographic expansions and recolonization of formerly unsuitable areas (Godfrey M. Hewitt 2001). In contrast, the Interglacial Refugia model proposes the opposite dynamic: glacial periods favored range expansion and connectivity of populations in lowland areas, while interglacial warming led to contraction into isolated refugia at higher elevations or wetter habitats (Haffer 1969; Leite et al. 2016). This scenario predicts increased gene flow and reduced population structure during glacial times, with less pronounced genetic divergence due to more recent isolation events (Schoville et al. 2011; Ramírez‐Barahona and Eguiarte 2013; Mastretta‐Yanes et al. 2015). Both models predict contrasting genetic outcomes, ranging from increased differentiation to diffuse phylogeographic structure, depending on species' dispersal abilities, ecological requirements, geographic range size, and the topographical complexity of their habitats (Hernández‐Langford et al. 2020; Mastretta‐Yanes et al. 2015; Ramírez‐Barahona and Eguiarte 2013).

Empirical support for each model has been documented across a range of environments in Mexico. For example, phylogeographic and paleo‐distribution data consistent with both the Glacial Refugia and Interglacial Refugia models have been reported in arid (e.g., Aguirre‐Planter et al. 2020; Cornejo‐Romero et al. 2017), temperate (e.g., Zamudio‐Beltrán et al. 2020; Osuna et al. 2020), and tropical environments (e.g., Ornelas et al. 2016; Hernández‐Langford et al. 2020). Moreover, central and southern Mexico are notable for their exceptional biological and environmental complexity (Morrone 2010), which likely contributes to the diversity of responses observed among taxa. Overall, these findings show that even closely related, co‐distributed, and ecologically similar species can exhibit divergent responses to the Pleistocene climate changes. Despite increasing evidence, our understanding of how these historical processes have shaped the evolutionary trajectories of Mexican biotas remains incomplete (Peñaloza‐Ramírez et al. 2020). This is especially true for Neotropical insects, which, despite their high ecological and economic relevance, remain underrepresented in phylogeographic studies (Menezes et al. 2020).

**FIGURE 1:**
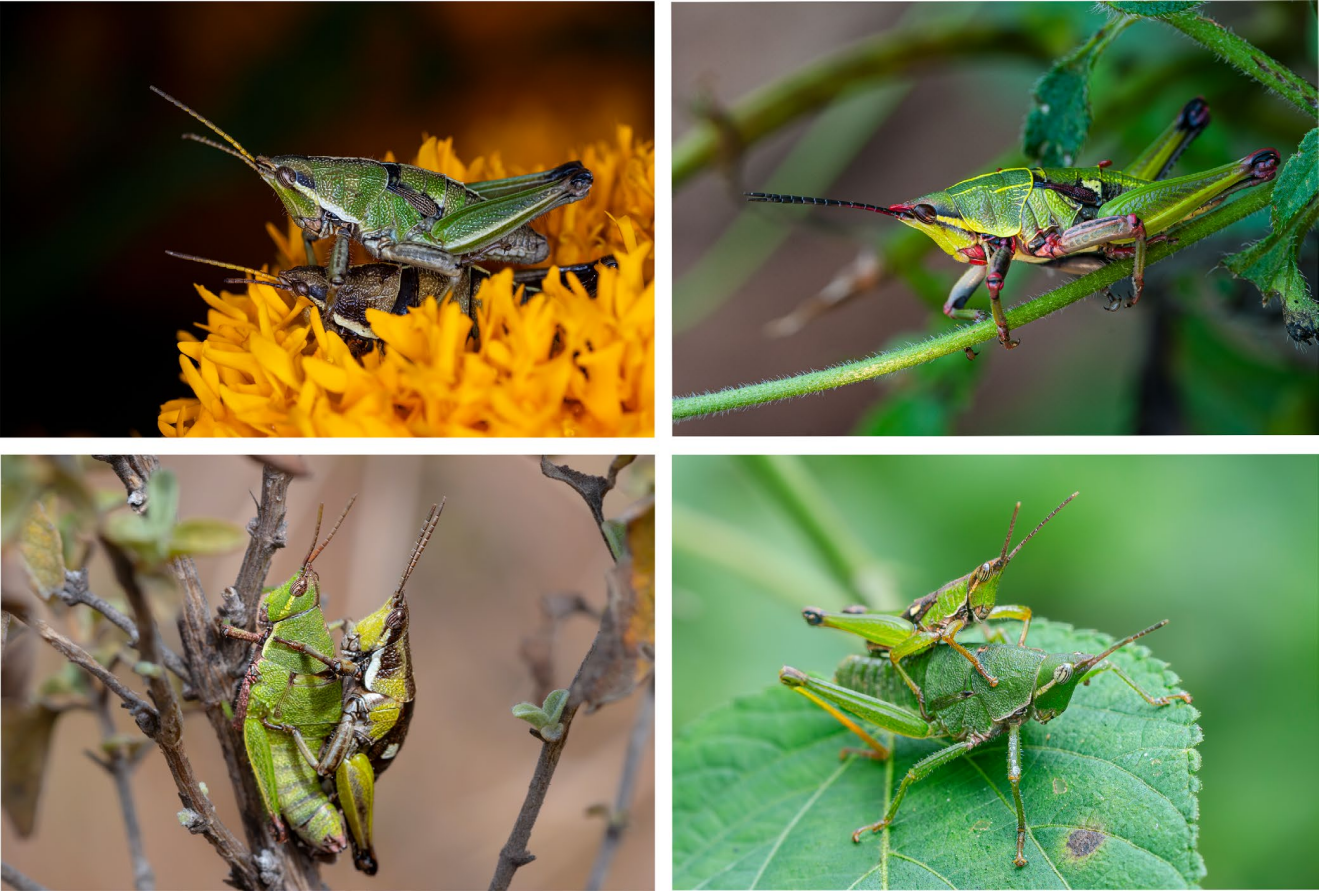
Field photographs of the four *Sphenarium* species studied: *S. purpurascens* (top‐left), *S. rugosum* (top‐right), *S. variabile* (bottom‐left), and *S. zapotecum* (bottom‐right). Mating pairs (male mounted on female) are shown for all species except *S. rugosum* , which depicts a single adult male. Photographs of *S. purpurascens* and *S. rugosum* were provided by Leopoldo D. Vázquez‐Reyes (BioPic A.C.—AMFC).

In this study, we evaluated whether genetic and geographic patterns in Neotropical grasshoppers of the genus *Sphenarium* Charpentier, 1842 correspond with those predicted by the Glacial Refugia or the Interglacial Refugia model. These species are both significant crop pests and edible insects that have been consumed in Mexico since pre‐Columbian times (Cerritos and Cano‐Santana 2008; Sanabria‐Urbán et al. 2017). *Sphenarium* species are univoltine, flightless, and polyphagous herbivores that inhabit open areas of different environments from central Mexico to northwestern Guatemala (Sanabria‐Urbán and del Castillo 2020). We focused on four species that diverged during the Pleistocene and are distributed parapatrically (Sanabria‐Urbán et al. 2017): *S. purpurascens* , *S. rugosum* , *S. variabile* , and *S. zapotecum* (Figure 1). The species *S. purpurascens* is widely distributed in temperate and xeric highlands from the Mexican Plateau to the Sierra Madre del Sur. *Sphenarium variabile* and *S. zapotecum* occur in the temperate highlands and humid Pacific slope, respectively, of the Sierra Madre del Sur in Oaxaca. Lastly, *S. rugosum* inhabits tropical and temperate environments in the Balsas River Basin (Sanabria‐Urbán et al. 2017). Although previous phylogenetic studies suggested close relationships among these species (Sanabria‐Urbán et al. 2015, 2017), a recent phylogenomic analysis revealed more distant relationships and widespread mitochondrial DNA (mtDNA) introgression among *S. purpurascens* , *S. variabile* , and *S. zapotecum* (Benites et al. 2023). Although introgression can limit the utility of mtDNA for resolving species‐level phylogenies (Joly et al. 2009), the spatial distribution of mtDNA lineages can still be informative about historical patterns of isolation and connection, especially when genetic structure is detected (Bryson et al. 2010, 2014; Marková et al. 2013; Mulcahy et al. 2006).

Because *Sphenarium* grasshoppers are ectotherms with low dispersal capability, we hypothesize that their evolutionary history and phylogeographic patterns have been strongly influenced by Pleistocene climatic dynamics. Moreover, cycles of habitat isolation–connection during glacial–interglacial cycles could have driven divergence among *Sphenarium* lineages via local adaptation to different environmental conditions or even ecological niche conservatism (see Peterson et al. 2011 for a more detailed explanation). Although niche conservatism is commonly associated with allopatric speciation, the parapatric distribution of these four species suggests that climatic differences may have promoted population differentiation and niche divergence, as documented in other montane Mexican taxa (e.g., Moreno‐Contreras et al. 2020).

To test these hypotheses, we conducted a phylogeographic analysis using mtDNA sequences to examine spatial and temporal patterns of genetic variation among these neotropical grasshoppers. Our aim was to assess whether the observed genetic structure and demographic history are more consistent with expectations from the Glacial Refugia or Interglacial Refugia models. We used mtDNA as a tool to detect broad signatures of population differentiation and historical range dynamics among these four *Sphenarium* species. We then complemented this data by using ecological niche modeling (ENM) to evaluate the niche conservatism hypothesis and reconstruct paleo‐distribution patterns during the last glacial and interglacial episodes among the four studied species. Together, these analyses allow us to explore how past climatic fluctuations may have shaped the genetic and ecological diversity of these neotropical grasshoppers.

## 2 Materials and Methods

### 2.1 Sample Collection

**FIGURE 2:**
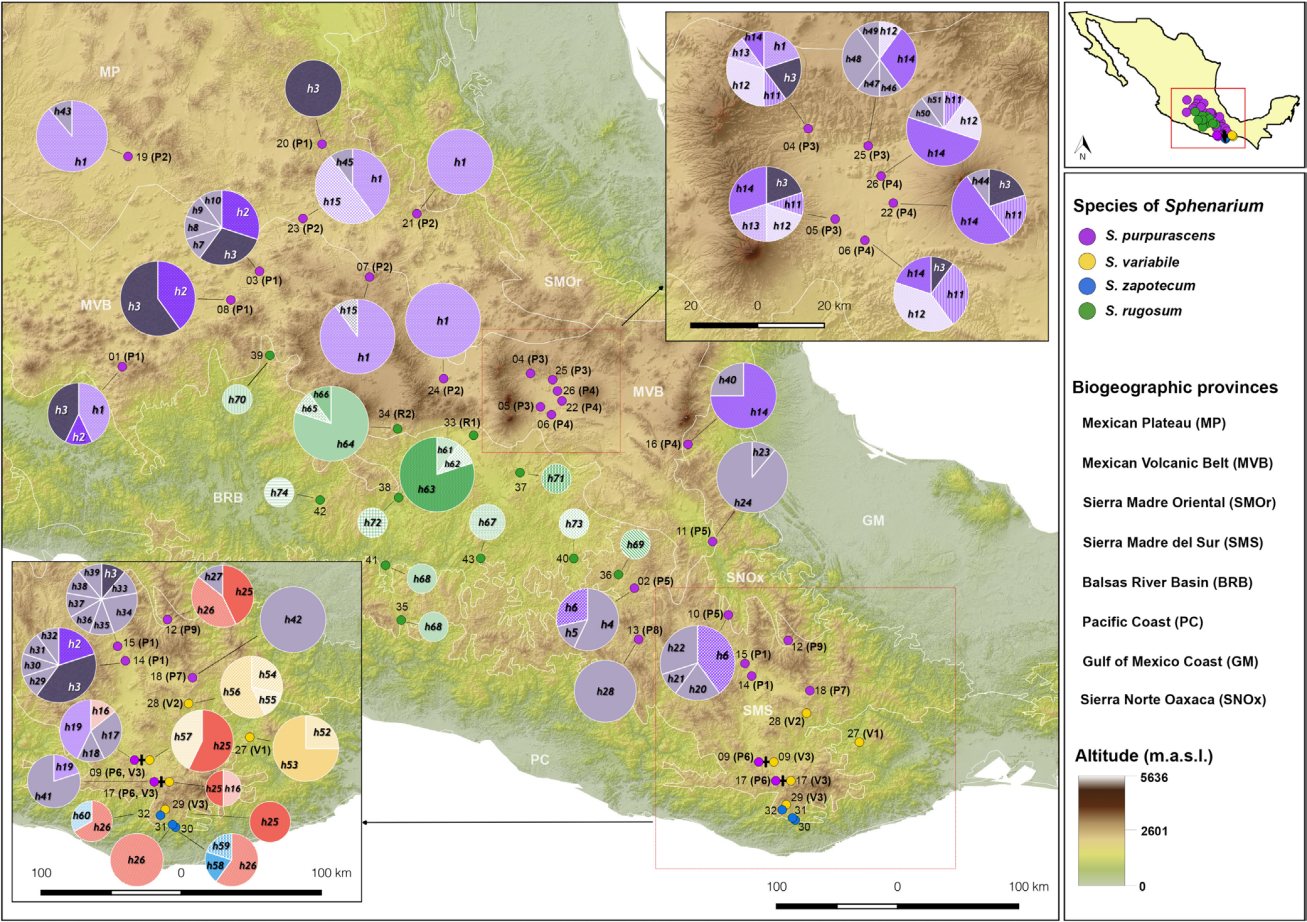
Geographic distribution of CO1 haplotypes of four *Sphenarium* species. Sampling localities are represented by small colored dots with black labels indicating the locality number and population (numbers in parenthesis) to which each locality was assigned according to the genetic structure analysis. Pie charts show the relative frequency of the haplotypes identified within each locality (labels in italics) with the size of the chart proportional to the sample size at the locality. For simplicity, all locally restricted haplotypes identified *S. purpurascens* are in solid pale‐purple, while haplotypes that are shared among localities are represented by distinct purple patterns in the pie plots. For the other three species, distinct colored patterns indicate different haplotypes. Patterns with the same base color (purple, yellow, blue, or green) in the pie plots indicate species‐specific haplotypes ( *S. purpurascens* , *S. variabile* , *S. zapotecum* or *S. rugosum* , respectively); whereas shared haplotypes among species are denoted by different patterns in red.

The genetic analyses in this study relied on previously collected samples (2012–2018) of *S. purpurascens* , *S. variabile* , *S. zapotecum*, and *S. rugosum* across their known geographic distribution ranges, as defined by prior studies (Sanabria‐Urbán et al. 2017). Sampling sites were selected to represent both central and peripheral portions of each species' range, with the goal of capturing potential geographic and genetic structure. The number of sites and individuals varied among species, reflecting differences in their distributional extent and specimen availability. Overall, we included 45 sampling localities: 227 *S. purpurascens* individuals from 26 localities, 30 *S. rugosum* from 11 localities, 27 *S. variabile* from 5 localities, and 13 *S. zapotecum* from 3 localities (Figure 2). The number of individuals per locality ranged from 1 to 10, but in most cases, 5–10 individuals were collected per site (Table S1). We included both male and female specimens from most sites (43 out of 45). All but two localities had only one species. At localities L9 and L17, *S. purpurascens* and *S. variabile* were sympatric (Figure 2); thus, we included only males in our analyses to ensure accurate species identification based on the most recent taxonomic revision of the genus (Sanabria‐Urbán et al. 2017). Detailed information on each specimen and the geographic coordinates of the sampling localities are provided in Table S1. All specimens were stored at −80°C in individual vials and vouchered in the Laboratory of Genetic and Molecular Ecology (ENES Morelia, UNAM) and Laboratory of Ecology, UBIPRO (FES‐Iztacala; UNAM). Specimens are available upon request to the corresponding author.

### 2.2 DNA Extraction and Sequencing

Previous phylogenetic studies in the genus *Sphenarium* have reported relatively high levels of polymorphism in the mitochondrial gene *Cytochrome c Oxidase subunit 1* or CO1 (Sanabria‐Urbán et al. 2015, 2017). Based on this, we selected this locus for our genetic analyses. Genomic DNA was extracted from a single hind femur of each specimen using the Qiagen DNeasy Blood and Tissue Kit (Qiagen). A 1065 bp fragment of the CO1 gene was amplified following the protocols and conditions described in Sanabria‐Urbán et al. (2015). Single‐band PCR products were purified using the PrepEase Purification 96‐well plate kit (USB Corporation). Both DNA strands were sequenced using the BigDye Terminator v3.1 Cycle Sequencing Kit (Applied Biosystems) on a 3730XL DNA Analyzer (Applied Biosystems). We assembled and edited forward and reverse sequences of each sample using Sequencher 4.2 (Gene Codes Corporation) under default parameters. Sequences were translated in MEGA X (Kumar et al. 2018) to check for stop codons and to confirm their identity as CO1 sequences by comparison with previously reported sequences. All newly generated sequences were deposited in GenBank (Benson et al. 2017). Table S1 shows the accession numbers of these sequences.

### 2.3 Genetic Diversity Analyses

We used DNAsp v6 (Rozas et al. 2017) to estimate the haplotype diversity (Hd) and nucleotide diversity (*π*), as well as their standard errors (SE) for each species, considering the total number of individuals obtained. These genetic diversity parameters were also estimated at the intraspecific levels for *S. purpurascens* , *S. variabile* , and *S. rugosum* , considering the populations inferred by the genetic structure analyses (see Section 3). These diversity estimates were based on unequal numbers of individuals among species and populations, as indicated in Table 1. However, in all cases, sample sizes were ≥ 5 individuals, which have been shown to be sufficient to distinguish between low and high levels of genetic diversity using mitochondrial markers (Goodall‐Copestake et al. 2012).

**TABLE 1:** Estimates of genetic diversity and demographic parameters of four S*phenarium* species and their populations.

Species and populations	*l*	*n*	*h*	*e*	*s*	Hd (SE)	𝜋 (SE)	Tajima's *D*	Fu's *Fs*
*S. purpurascens*	26	227	51	77	73	0.92 (0.009)	0.009 (0.0002)	−0.51	−8.01
P1 (L1, L3, L8, L14, L15, and L20)	6	52	19	39	39	0.78 (0.054)	0.005 (0.001)	−1.17	−3.09
P2 (L7, L19, L21, L23, and L24)	5	47	4	4	4	0.30 (0.080)	0.0003 (0.0001)	−1.40	−1.66
P3 (L4, L5, and L25)	3	30	10	28	28	0.89 (0.031)	0.008 (0.001)	0.93	2.94
P4 (L6, L16, L22, and L26)	4	38	8	5	5	0.73 (0.061)	0.001 (0.0002)	0.40	−2.20
P5 (L2, L10, and L11)	3	26	9	10	10	0.85 (0.043)	0.002 (0.0002)	−1.02	−1.95
P6 (L9 and L17)	2	12	5	6	6	0.80 (0.078)	0.002 (0.0004)	−0.32	1.09
P7 (L18)	1	8	1	—	—	—	—	—	—
P8 (L13)	1	7	1	—	—	—	—	—	—
P9 (L12)	1	7	3	18	18	0.71 (0.127)	0.008 (0.001)	−1.15	5.54
*S. variabile*	5	40	8	27	27	0.87 (0.028)	0.005 (0.0006)	1.81	4.85
V1 (L27)	1	8	3	2	2	0.71 (0.123)	0.0008 (0.0002)	−0.10	3.16
V2 (L28)	1	7	3	2	2	0.67 (0.160)	0.0007 (0.0002)	0.33	0.54
V3 (L9, L17, and L29)	3	12	3	2	2	0.53 (0.136)	0.0009 (0.0002)	1.41	5.42
*S. rugosum*	11	30	14	72	68	0.89 (0.042)	0.013 (0.0012)	1.75	6.21
R1 (L33)	1	10	3	2	2	0.38 (0.181)	0.0004 (0.0002)	−1.40	1.16
R2 (L34)	1	10	4	14	14	0.53 (0.180)	0.004 (0.0019)	−0.16	2.85
*S. zapotecum* (L30, L31, and L32)	3	13	4	27	26	0.42 (0.164)	0.005 (0.0024)	−1.83*	4.84

*Note:* Sampling localities included within each population are shown in parentheses.

Abbreviations: 𝜋, nucleotide diversity; *h*, number of haplotypes; Hd, haplotype diversity; *l*, number of sampling localities; *m*, number of mutations; *n*, sample size; *s*, number of segregating sites; SE, standard error.

* Significant values at *p* < 0.05.

### 2.4 Phylogeographic and Genetic Structure Analyses

To minimize potential bias in the inference of genetic structure due to unequal sample sizes, we excluded all sampling sites with fewer than three individuals. As a result, only two localities (L33 and L34) were retained for *S. rugosum* . For the remaining species, three sites with three individuals per site were included (L17 and L29 for *S. variabile* , and L32 for *S. zapotecum*), while all remaining sites had 5–10 individuals per site (Table S1). To assess phylogeographic structure, we calculated population differentiation coefficients based on unordered (*G*_ST_) and ordered (*N*_ST_) haplotypes using SPADS 1.0 (Dellicour and Mardulyn 2014). The recommended sampling size for optimal estimates of these indices ranges from approximately 5 to 7 individuals per population (Pons and Petit 1996), which aligns with most of our dataset. We used 10,000 permutations to test the significance of *N*_ST_ versus *G*_ST_: a significantly higher *N*_ST_ than *G*_ST_ indicates phylogeographic structure, meaning that closely related haplotypes are spatially clustered.

For species with at least three localities showing significant genetic differentiation ( *S. purpurascens* and *S. variabile* ; see Section 3), we performed spatial analyses of molecular variance (SAMOVAs; Dupanloup et al. 2002) using SPADS to infer the optimal number of geographically homogeneous and maximally differentiated groups (with higher *F*_CT_ values). We tested values of *K* (number of groups) ranging from 2 to 15 for *S. purpurascens* and from 2 to 4 for *S. variabile* , with 10 replicates per *K* value. To quantify genetic differentiation among the identified groups, we calculated pairwise *F*_ST_ values using the distance method implemented in Arlequin 3.5 (Excoffier and Lischer 2010), assessing significance with 10,000 permutations. Moreover, for each species and for populations comprising at least three localities, we performed Mantel tests to evaluate the correlation between genetic (*F*_ST_/1 − *F*_ST_) and geographic distances (i.e., isolation by distance). Significance for Mantel test was also assessed with 10,000 permutations. Both SAMOVA and Arlequin implement Weir and Cockerham's (1984) *F*‐statistics with weighted variances, which account for unequal sample sizes (Excoffier et al. 1992; Michalakis and Excoffier 1996; Dupanloup et al. 2002).

### 2.5 Phylogenetic Analysis and Divergence Time Estimation

We used BEAST 1.10.4 (Suchard et al. 2018) to infer phylogenetic relationships and estimate divergence times among the haplotypes identified in the four studied species of *Sphenarium*. For the outgroups, we retrieved from GenBank CO1 sequences of five *Sphenarium* species that are closely related to the study species (Sanabria‐Urbán et al. 2017): *S*. *crypticum*, *S. macrophallicum*, *S. minimum* , *S. planum* , and *S. tarascum* (accession numbers are in Table S1). We compiled a dataset of all CO1 sequences and aligned them using the software Muscle (Edgar 2004) with default parameters. The alignment was initially partitioned by codon positions, and we used PartitionFinder 2 (Lanfear et al. 2014) to identify the best partitioning scheme and substitution model, applying the greedy algorithm and the Bayesian Information Criterion. The results indicated that all codon positions could be treated as a single partition evolving under the HKY + I + G model.

In BEAST, we performed Markov chain Monte Carlo simulations using a Yule tree model and a strict molecular clock. The substitution rate parameter was given a uniform prior ranging from 1.15 × 10^−2^ to 2.0 × 10^−2^ substitutions/site^−1^/million years^−1^, reflecting rates previously reported for insects (Brower 1994) and Orthoptera (Shapiro et al. 2006; Allegrucci et al. 2011). We ran three independent analyses of 20 million generations each, sampling every 2000 generations. Convergence and mixing were assessed in Tracer v.1.6 (Rambaut and Drummond 2013), and all parameters had effective sample sizes (ESS) > 200. We discarded the first 25% of samples as burn‐in and generated a maximum clade credibility tree with TreeAnnotator in BEAST. To visualize haplotype distributions in each species, we constructed a haplotype network using the statistical parsimony method (Clement et al. 2000) implemented in PopART (Leigh and Bryant 2015) with the default settings.

### 2.6 Historical Demography and Migration

We explored the demographic history of the studied species and their populations using neutrality tests and coalescent‐based approaches. In Arlequin, we estimated the Fu's *Fs* (Fu 1997) and Tajima's *D* (Tajima 1989), evaluating their significance with 10,000 simulations. The significance of these parameters demonstrates a deviation in the samples from the equilibrium between mutation and drift, indicating phenomena such as rapid range expansion or population “bottlenecks” (Schneider and Excoffier 1999). In addition, we performed Bayesian Coalescent Bayesian Skyline Plot (BSP) analyses in BEAST to infer changes in effective population size (*N*_e_) over time for each species and population (except P7 and P8 of *S. purpurascens* , which had only one haplotype). Substitution models for each dataset were selected using PartitionFinder 2. BSP analyses used either 5 or 10 segments, depending on the number of coalescent events in each dataset (see Table S2 for more details). We applied a strict molecular clock with a uniform substitution rate that was previously converted to a per‐year rate (1.15 × 10^−8^ to 2.0 × 10^−8^ substitutions/site/year), as in the divergence time analysis. All other priors were left at default values. Each analysis was run twice for 40 × 10^6^ generations, sampling every 4000 steps and discarding the first 10% as burn‐in. We confirmed convergence and ESS > 200 for all parameters using Tracer.

**FIGURE 3:**
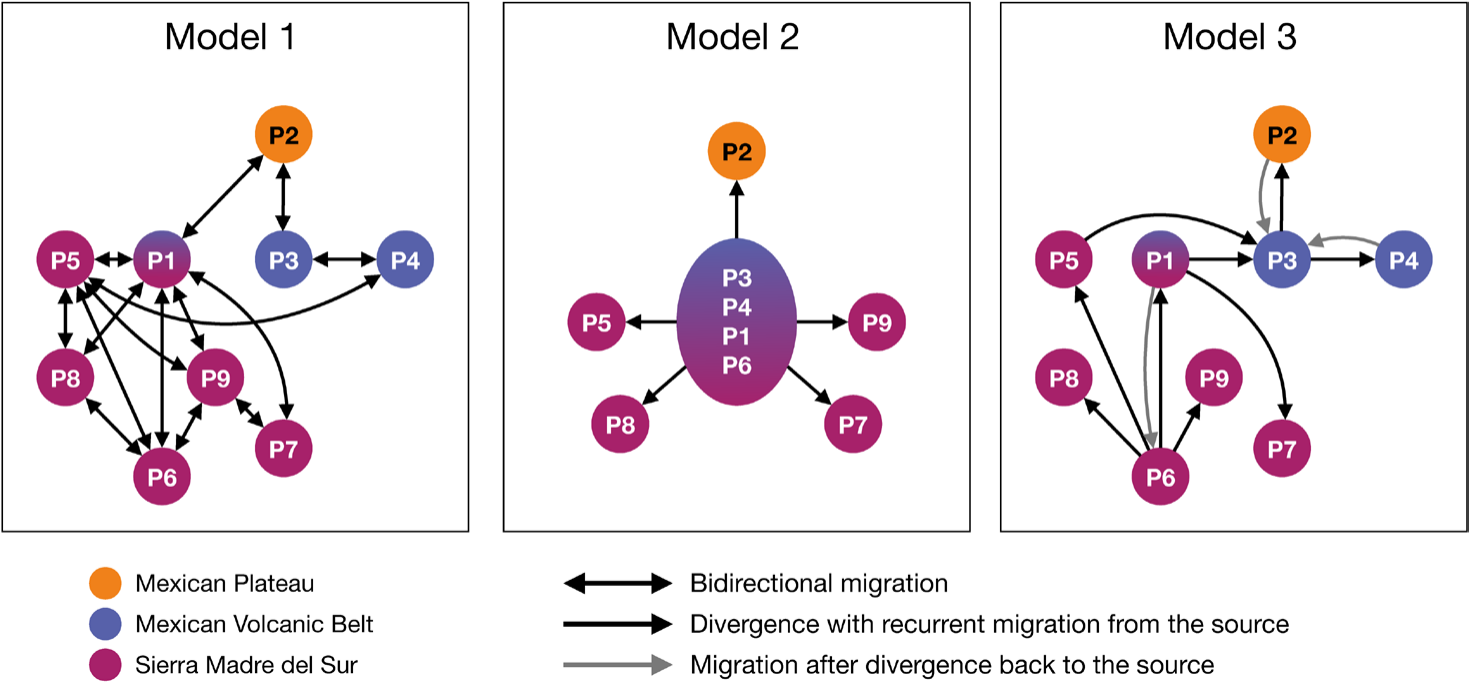
Schematics of the models of migration built to evaluate historical gene flow and divergence patterns between populations of *Sphenarium purpurascens*. Arrows indicate migration or divergence among populations (circles). The arrangement of the populations approximately reflects their geographic distribution (from south to north) within the biogeographic regions of central and southern Mexico. For simplicity, the full model assuming free bidirectional migration among all populations is not shown.

Because we detected shared haplotypes among several sampling localities of *S. purpurascens* (see Section 3), we evaluated historical gene flow among the inferred genetic groups using coalescent‐based Bayesian analysis in MIGRATE‐N v5.0.4 (Beerli 2006; Beerli and Felsenstein 2001; Beerli and Palczewski 2010). We tested four migration models (Figure 3): (i) unrestricted gene flow among all populations (Full model); (ii) bidirectional migration between neighbor populations (model 1); (iii) formation of a panmictic unit among central populations that share haplotypes (P1, P3, P4, and P6) with peripheral populations (P7, P9, P5, P8, and P2) diverging from this unit while maintaining recurrent migration (model 2); and (iv) a south‐to‐north divergence pathway (P6 → P1 → P3 → P4, P3 → P2, and P5 → P3) with recurrent migration from the source, as well as migration back to the source for some derived populations (P6 ← P1, P3 ← P4, and P3 ← P2), considering the southernmost populations (P1 and P6) as the source for other peripheral populations (P7, P9, P5, and P8) in the south (model 3 [most complex]). This last model was based on the observed haplotype distribution patterns and *F*_ST_ values for the populations of this species.

Analyses in migrate‐N used a HKY substitution model (with transition/transversion ratio = 4.7), a static heating scheme with four temperatures (1.00, 1.50, 3.00, and 1,000,000), and default settings for all other parameters. We ran three replicates per model, each with a single long chain of 500,000 steps, sampling every 100 generations and discarding the first 100,000 steps as burn‐in. Model selection was based on log Bayes factor (LBF) computed with the R package “*mtraceR*” (Pacioni et al. 2015), using the thermodynamic integration value (BTI) from the Migrate‐N outputs. LBF values less than 2 indicated strong preference for the top model (Kass and Raftery 1995).

### 2.7 Niche Conservatism Analyses and Paleodistribution Modeling

To assess the variation of environmental conditions across the distribution of the four *Sphenarium* species, we applied ENM as an approximation of their Grinnellian niches (Rödder and Engler 2011). In this sense, we analyzed whether the observed phylogeographic pattern among species corresponded to a niche divergence shaped by geography and particular climate conditions for the same environmental factors (Salariato and Zuloaga 2017). Specifically, we correlated the field occurrence data with environmental variables to estimate niche overlap and the potential distribution of suitability areas for each taxon (Peterson et al. 2011). The dataset used included species records from Sanabria‐Urbán et al. (2017) and recent field surveys (Table S3). To reduce sampling bias, we used the “*spThin*” R package (Aiello‐Lammens et al. 2015) to spatially rarefy the records, optimizing the minimum distance between occurrence points. The final dataset comprised 136 spatially rarefied records: 102 for *S. purpurascens* , 12 for *S. variabile* , 8 for *S. zapotecum*, and 51 for *S. rugosum* . Additionally, we delimited the accessible area (“M” in the BAM framework; Soberon and Peterson 2005) for each species using a simulation‐based method implemented in the “*Grinnell*” R library (Machado‐Stredel et al. 2021).

To characterize the ecological niches, we selected a set of uncorrelated (Spearman coefficient < 0.8) climatic variables from WorldClim 2.1 (Hijmans et al. 2005; Fick and Hijmans 2017), at ~5 km^2^ (0.041665°) spatial resolution. We prioritized variables representing annual or seasonal climate trends, as suggested by Shabani et al. (2019). The selected variables were: annual mean temperature (AMT), mean diurnal range (MDR), isothermality (ISO), mean temperature of wettest quarter (MTWQ), mean temperature of coldest quarter (MTCQ), annual precipitation (PREC), precipitation of driest month (PDM), precipitation seasonality (PSE), and precipitation of driest quarter (PDQ). Furthermore, since habitat preferences are considered important drivers of speciation in tropical regions (e.g., Warren et al. 2008; Salisbury et al. 2012), we generated an alternative set of present‐day predictors by combining climatic variables with elevation (see USGS 2001) and vegetation heterogeneity (measured as dissimilarity in the Enhanced Vegetation Index; see Tuanmu and Jetz 2015). This allowed us to evaluate the influence of additional environmental factors on niche differentiation. Nevertheless, we found no substantial differences in niche differentiation patterns (see below) between the “climatic only data” and “climate‐plus‐habitat” models (see Table S6). Therefore, to ensure consistency with projections to paleoclimatic scenarios for which only climatic data were available, we present here the results from climate‐only models.

Our first approach to evaluating the role of ecological and geographical drivers in lineage diversification was to test whether the ecological niches used by the taxa were more similar than expected by chance. We used the PCA‐env method (Broennimann et al. 2012), which included: (1) estimating the density of occurrences and environmental conditions (based on the selected climatic variables) along principal component axes; (2) calculating niche overlap using *Schoener*'s *D* index (ranging from 0 [no overlap] to 1 [complete overlap]; Rödder and Engler 2011); and (3) performing statistical tests (niche equivalency vs. background similarity) to compare the empirically observed distributions of Schoener's *D* to 1000 randomly generated simulated values (see Warren et al. 2008; Broennimann et al. 2012). Niche equivalency tests evaluate whether two species occupy statistically indistinguishable environmental spaces using Monte Carlo resampling, while background similarity tests assess whether observed overlap is greater than expected given random distributions of occurrences within each species' accessible area (Broennimann et al. 2012; Brown and Carnaval 2019). Importantly, the PCA‐env framework estimates species' environmental occupancy using a kernel density function, which generates a smoothed density of occurrences across environmental space and is particularly robust to small sample sizes (see Broennimann et al. 2012). In both tests, we considered niche conservatism to be supported when the observed *D* values were significantly higher (*p* < 0.05) than those from null distributions. All analyses were performed using the R packages “*ecospat*” (Di Cola et al. 2017) and “*Humboldt*” (Brown and Carnaval 2019).

As a second approach to evaluating the role of geographical drivers, we generated suitability maps to estimate the potential geographic distributions of the four *Sphenarium* species during the Late Pleistocene climate cycles. We used MaxEnt 3.4.4k (Phillips et al. 2017) following the ENM calibration protocol of Muscarella et al. (2014) to select optimal model parameters and evaluate model performance (Table S5). To do this, we used two R packages: “*kuenm*” (Cobos et al. 2019) for species with > 20 independent records and “*ENMeval*” (Kass et al. 2021) for those with 8–12 records. In the cases of *S. variabile* and *S. zapotecum*, final models were built using a leave‐one‐out approach (i.e., Jackknife method; Pearson et al. 2007) to evaluate model performance and by partitioning the occurrence localities into training and testing sets using the *n*‐fold cross‐validation method implemented via the *partition_type* function in the “*ENMeval*” (Kass et al. 2021). For all species, we projected suitable conditions onto paleoclimatic reconstructions for three periods: one glacial (the Last Glacial Maximum [LGM], ~21 kya) and two interglacial (the Mid‐Holocene, ~6 kya; and the Last Interglacial [LIG], ~120 kya). Binary presence‐absence maps were generated using the 10th percentile training presence threshold (Liu et al. 2013). For this study, past climate data for the Mid‐Holocene and LGM were based on three global climate circular models (CCSM, MIROC, and MPI‐ESM; see Cooper et al. 2016), while for the LIG only the CCSM model was available (Otto‐Bliesner et al. 2006). We also assessed the reliability of our model projections by calculating the mobility‐oriented parity (MOP) metric (Owens et al. 2013).

We then examined the potential historical range extension, connectivity, and stable areas that may have influenced the divergence and persistence of these four *Sphenarium* species. To do this, we specifically calculated geographic overlap (i.e., alloprediction) between the species' predicted distributions across different paleoclimatic scenarios. We hypothesized that the projected ranges would be similar among species if their climatic niches were historically conserved and ecologically similar, regardless of geographic barriers (e.g., Peterson et al. 2011; Castillo‐Chora et al. 2021; Rivera‐Ortíz et al. 2023). Finally, for each species, we identified areas of long‐term climatic stability (i.e., areas deemed suitable in all scenarios analyzed) as potential refugia (Terribile et al. 2012) and analyzed overlaps among species in these stable areas.

## 3 Results

### 3.1 Haplotype Distribution

We identified 74 distinct CO1 haplotypes in the 297 sequenced individuals across the four species of *Sphenarium*: 51 in *S. purpurascens* , 8 in *S. variabile* , 4 in *S. zapotecum*, and 14 in *S. rugosum* (Table 1; Figure 2). The number of segregating sites (S) per species was 73 in *S. purpurascens* , 27 in *S. variabile* , 26 in *S. zapotecum*, and 68 in *S. rugosum* (Table 1). Notably, the only species that had shared haplotypes across multiple localities was *S. purpurascens* , with 10 haplotypes shared, including the most frequent ones (h1, h2, h3, h12, and h14). Of these, two haplotypes (h2 and h3) were widely distributed across *S. purpurascens* collection localities, while eight haplotypes (h1, h6, h11, h12, h13, h14, h15, and h19) were shared among geographically proximate localities within the same region—the Mexican Plateau, the Mexican Volcanic Belt, or the Sierra Madre del Sur (Figure 2). All other haplotypes in *S. purpurascens, S. variabile*, and *S. zapotecum* were exclusive to a single locality (Figure 2).

Three of the 74 haplotypes (h16, h25, h6) were shared among different species in the Sierra Madre del Sur region: h16 and h25 were shared between *S. purpurascens* and *S. variabile* individuals, whereas h26 was shared between *S. purpurascens* and *S. zapotecum* individuals. Haplotype h16 was detected in adjacent localities (L9 and L17), while h25 and h26 were found in more distant and isolated localities (Figure 2). *Sphenarium rugosum* did not share haplotypes with the other species, and most of its haplotypes were restricted to a single locality, except h68, which was shared between two neighboring localities (L35 and L41) in the Central Balsas River Basin.

### 3.2 Genetic Structure

**FIGURE 4:**
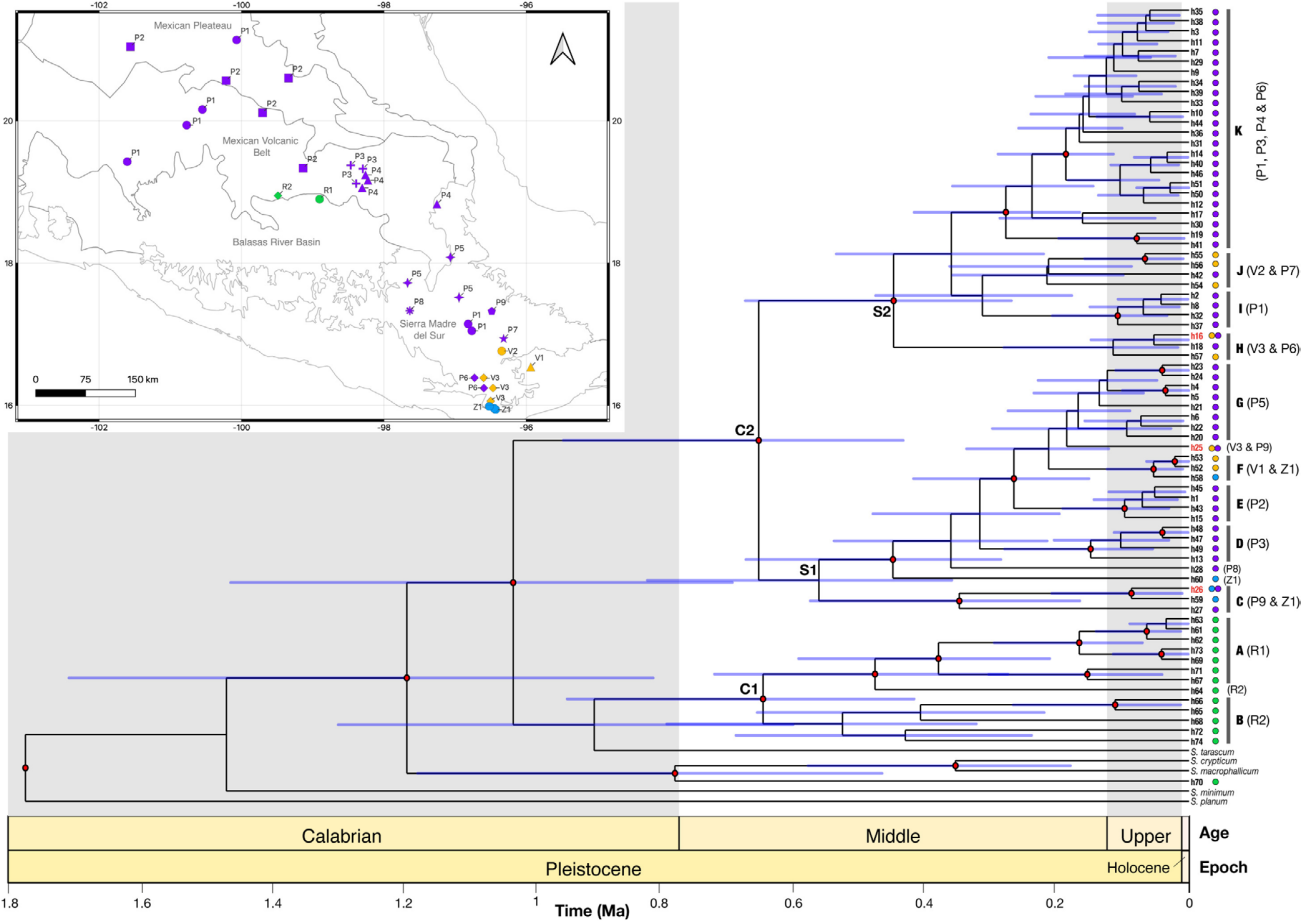
Time‐calibrated Bayesian phylogeny of mitochondrial CO1 haplotypes from the four *Sphenarium* species studied and their closest relatives. Red dots denote nodes supported by posterior probabilities ≥ 0.9, while blue shaded bars represent the 95% credibility intervals for node ages. Colored dots next to haplotype names (h1–h74) indicate the species in which they were identified: purple for *S. purpurascens* , yellow for *S. variabile* , blue for *S. zapotecum*, and green for *S. rugosum* . Labels on nodes and in front of tips highlight the major mitochondrial clades (C1, C2, S1, and S2), haplogroups (A–K), and the populations (noted in parentheses) where they were found. The map in the top‐left corner illustrates the geographic distribution of the populations (different symbols) identified by the genetic structure analyses, using the same color scheme as described above.

Genetic differentiation (*G*_ST_ and *N*_ST_) was moderate to strong and statistically significant for all species except for *S. zapotecum* (Table 2). All localities of *S. zapotecum* were therefore considered to constitute a single population. In *S. rugosum* , the only two localities sampled (L33 and L34) showed strong and significant genetic differentiation (*F*_ST_ = 0.85, Table 2) and were thus considered to constitute independent populations: R1 in the east and R2 in the northern Balsas Basin (Figures 2 and 4; Table 1). In *S. purpurascens* , *S. variabile* , and *S. rugosum* , *N*_ST_ values were significantly higher than *G*_ST_ values (Table 2, *N*_ST_–*G*_ST_), indicating strong phylogeographic structure.

**TABLE 2:** Results of the permutation tests on ordered (*N*_ST_) and unordered (*G*_ST_) genetic differentiation values in the four studied species of *Sphenarium*.

Species	*G*_ST_	*N*_ST_	*N*_ST_–*G*_ST_
*S. purpurascens*	0.43**	0.72**	0.29**
*S. variabile*	0.33**	0.70**	0.37**
*S. rugosum*	0.54**	0.84*	0.30*
*S. zapotecum*	−0.03^ns^	−0.06 ^ns^	−0.03 ^ns^

Abbreviation: ns, nonsignificant.

* Significant values at *p* < 0.05.

** Significant values at *p* < 0.001.

The SAMOVAs detected strong geographic structure in *S. purpurascens* and *S. variabile* , identifying nine (*K* = 9, 𝛷_CT_ = 0.7) and three (*K* = 3, 𝛷_CT_ = 0.8) optimal population groupings, respectively (Figures 2 and 4; Table 1; Table S4). In *S. purpurascens* , population P1 included six localities spanning three biogeographic regions: L20 from the Mexican Plateau; L1, L3, and L8 from the Trans‐Mexican Volcanic Belt; and L14 and L15 from the Sierra Madre del Sur. The other eight populations of this species grouped localities within a single region: for instance, population P2 comprised most Mexican Plateau localities (L7, L19, L21, L23, and L24), populations P3 and P4 grouped eastern Mexican Volcanic Belt localities (P3: L4, L5, and L25—P4: L6, L16, L22, and L26), and the remaining populations corresponded to Sierra Madre del Sur localities (P5: L2, L10, and L11; P6: L9 and L17; P7: L18; P8: L13; and P9: L12) (Figures 2 and 4; Table 1). All populations' pairwise *F*_ST_ values were moderate–high and were highly significant (*F*_ST_ = 0.14 to 1, *p* < 0.001; see Table 3). In *S. variabile* , two populations (V1: L27–V2: L28) were each represented by a single locality, and a third population (V3) grouped L9, L17, and L29. These three populations were geographically separated (east, north, and south Sierra Madre del Sur, respectively) and showed high, significant differentiation among them (*F*_ST_ = 0.71–0.93, *p* < 0.0001; Figures 2 and 4; Table 4). Mantel tests revealed significant isolation by distance in both *S. variabile* and *S. purpurascens* , but this correlation was considerably higher in *S. variabile* (*r* = 0.86, *p* = 0.02) than in *S. purpurascens* (*r* = 0.16, *p* = 0.02). No significant correlations were found for *S. zapotecum*, *S. rugosum* , or any of their populations (data not shown), likely due to limited sample sizes.

**TABLE 3:** Pairwise *F*_ST_ comparisons among populations of *Sphenarium purpurascens* .

Populations	P1	P2	P3	P4	P5	P6	P7	P8	P9
P1	—								
P2	0.80**	—							
P3	0.14*	0.72**	—						
P4	0.29**	0.95**	0.25**	—					
P5	0.71**	0.80**	0.57**	0.90**	—				
P6	0.32*	0.93**	0.31*	0.72**	0.81**	—			
P7	0.57**	0.98**	0.48**	0.89**	0.87**	0.77**	—		
P8	0.70**	0.97**	0.53**	0.93**	0.73**	0.85**	1.00*	—	
P9	0.57**	0.84**	0.39*	0.83**	0.56**	0.58**	0.69*	0.52*	—

* Significant values at *p* < 0.001.

** Significant values at *p* < 0.00001.

**TABLE 4:** Pairwise *F*_ST_ comparisons among populations of *Sphenarium variabile*.

Populations	V1	V2	V3
V1	—		
V2	0.93**	—	
V3	0.71**	0.75**	—

** Significant values at *p* < 0.00001.

### 3.3 Genetic Diversity

High haplotype diversity was found in *S. purpurascens* (Hd = 0.92), *S. rugosum* (Hd = 0.89), and *S. variabile* (Hd = 0.87), while *S. zapotecum* exhibited moderate diversity (Hd = 0.42) (Table 1). However, *S. rugosum* showed the highest nucleotide diversity (𝜋 = 0.013), followed by *S. purpurascens* (𝜋 = 0.009), whereas *S. variabile* and *S. zapotecum* showed similar lower values (𝜋 = 0.0005) (Table 1). Within *S. purpurascens* , genetic diversity was moderate in population P2 (Hd = 0.3; 𝜋 = 0.0003) and high in most other populations (Hd = 0.71–0.89; 𝜋 = 0.001–0.005), except for P7 and P8, in which a single haplotype was found (Hd and 𝜋 = 0). *Sphenarium variabile* populations showed similarly high genetic diversity (Hd = 0.53–0.73; 𝜋 = 0.007–0.009); while in *S. rugosum* , diversity was moderate in R1 (Hd = 0.38; 𝜋 = 0.0004) and high in population R2 (Hd = 0.53; 𝜋 = 0.004; see Table 1).

### 3.4 Haplotype Relationships

**FIGURE 5:**
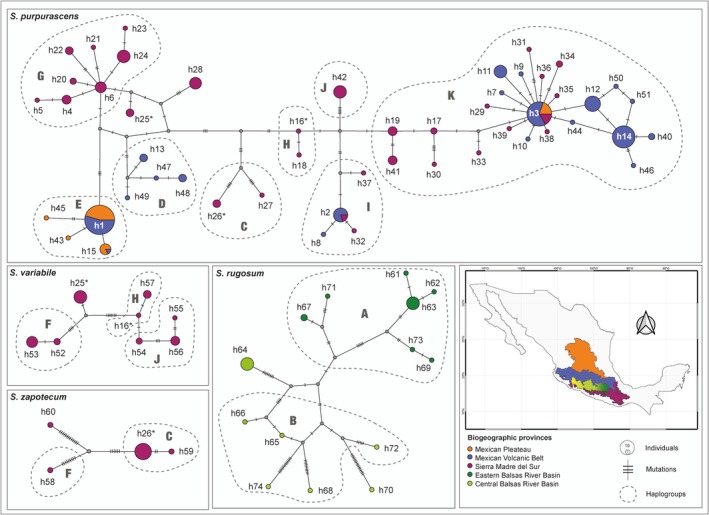
Haplotype network for the CO1 haplotypes of 127 individuals of *Sphenarium purpurascens*, *Sphenarium variabile*, *Sphenarium zapotecum*, and *Sphenarium rugosum*. Haplotypes are connected assuming a 95% parsimony threshold. Slashes across the lines connecting the haplotypes represent mutational steps. The size of the haplotypes (colored circles) is proportional to their frequency, and the dashed lines around them indicate the haplogroups (A–K) to which they belong. Colors of the haplotypes indicate their geographic distribution within the biogeographic regions of central and southern Mexico (see map on the bottom‐right). Haplotypes that are shared between species are denoted by an asterisk.

None of the four *Sphenarium* species formed a monophyletic group in the CO1 phylogenetic tree (Figure 4). Most *S. rugosum* haplotypes formed a well‐supported monophyletic clade (C1; Posterior Probability [PP] = 1), except for haplotype h70, which was paraphyletic relative to other species (Figure 4). Haplotypes from population R2 (h61, h62, and h63) of *S. rugosum* formed a monophyletic group (PP = 1) that was closely related to the eastern Balsas River Basin haplotypes (haplogroup A, PP = 0.9), while R1 haplotypes were paraphyletic and related to the central Balsas River Basin haplogroup B (PP = 0.8). The haplotypes of *S. purpurascens* , *S. variabile* , and *S. zapotecum* were also paraphyletic but formed a well‐supported clade (C2, PP = 1) that was subdivided into two major subclades (S1 [PP = 0.83] and S2 [PP = 0.92]), which were widely distributed from the Mexican Plateau to the Sierra Madre del Sur regions. Notably, within these subclades, we recognized eight haplogroups (C–K) that were mostly well‐supported (PP > 0.9), geographically restricted to a single region, and had poorly supported relationships among them (PP < 0.5). Some haplogroups were exclusive to one population (e.g., D, E, G, and I belonged to populations P3, P2, P5, and P1 of *S. purpurascens* , respectively), while others spanned populations (e.g., haplogroup *K* for populations P1, P3, P4, and P6 of *S. purpurascens* ) or species (haplogroups C, F, H, and J). Interestingly, populations V1, V2, and V3 of *S. variabile* comprised different haplogroups (F, J, and H, respectively), whereas haplotypes of *S. zapotecum* (with a single population) and *S. purpurascens* (P9) belonged to different haplogroups (see Figures 4 and 5).

BEAST analyses suggested very recent divergence among the mitochondrial lineages of the four species (Figure 4). The divergence time between haplotypes of *S. rugosum* and Clade 2 (comprising *S. purpurascens* , *S. variabile* , and *S. zapotecum*) was estimated at 1.03 Mya (95% HPD = 1.47–0.7 Mya), and most divergence within subclades or haplogroups was estimated to occur during the Middle Pleistocene (0.77 and 0.129 Mya). Additionally, haplotype network analyses showed that local haplotypes in *S. purpurascens* and *S. variabile* typically differed by few mutations (1–5) from widespread haplotypes (e.g., h1, h2, h3, and h14 of *S. purpurascens* ; h53 and h56 of *S. variabile* ). In contrast, *S. rugosum* and *S. zapotecum* haplotypes were more divergent (Figure 5). The most divergent haplotypes (h58 and h60) and those connecting the major network clusters (h57 and h16) were found in individuals from the Sierra Madre del Sur ( *S. purpurascens* , *S. variabile* , and *S. zapotecum*) or central Balsas River Basin ( *S. rugosum* ).

### 3.5 Historical Demography and Migration

**FIGURE 6:**
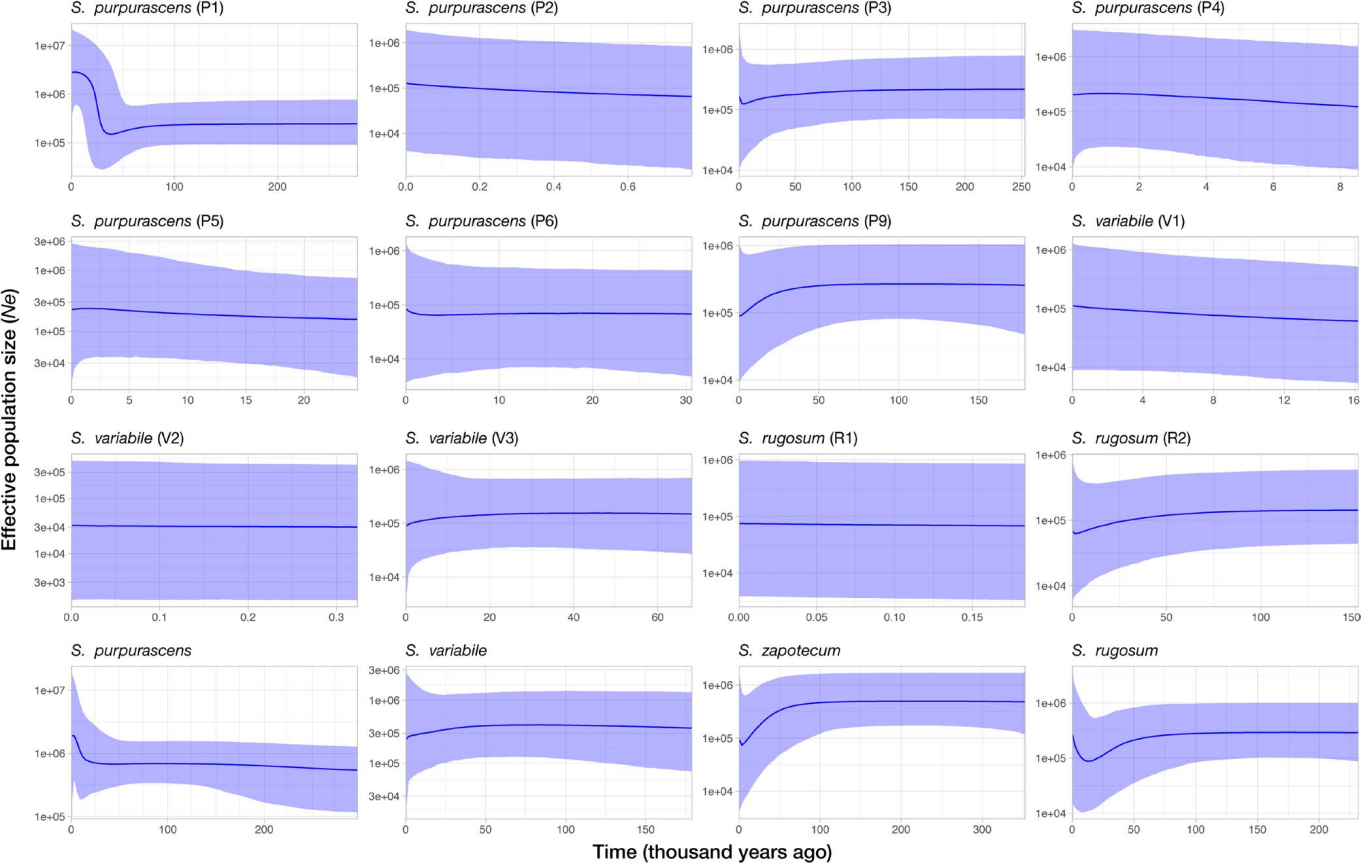
Bayesian skyline plots showing changes in effective population size (*N*_e_) for each population identified and pooling populations by species (bottom row). In all cases, the *x* axis represents time thousands of years ago (kya); while the *y* axis shows the scaled *N*_e_. Solid lines represent the median estimates and blue‐shaded areas represent 95% confidence intervals.

Tajima's *D* was significantly negative only for *S. zapotecum* (*D* = −1.83, *p* = 0.02), suggesting recent population expansion or bottleneck. No significant deviations were observed in other species or populations (Table 1). However, the Bayesian Skyline Plot for *S. purpurascens* population P1 indicated a continuous increase in *N*_e_ beginning ~22 kya, just before the Last Glacial Maximum (Figure 6). No significant demographic changes were detected in other populations, but species‐level analyses showed trends of increasing *N*_e_ in *S. purpurascens* (~25 kya) and *S. rugosum* (~9 kya), and a decline in *S. zapotecum* since ~50 kya, consistent with Tajima's *D*.

The migration analysis in Migrate‐N supported model 3 as the best‐fitting model (BIT = −2725.27, LBF = 0; Table 5; Figure 3), suggesting south‐to‐north divergence in *S. purpurascens* populations, with recurrent migration and central population P3 founded by two southern populations (P1 and P5). Moreover, this model suggests that the northernmost population (P2) likely diverged from P3, while isolated southern populations (P5, P7, P8, P9) diverged from the most southern (P6) or widespread (P1) populations.

**TABLE 5:** Results of model comparisons in MIGRATE‐N.

Model number	BIT	LBF	Choice rank	Probability
*Sphenarium purpurascens* models				
Full model	−2778.88	−107.22	4	0
Model 1	−2768.03	−85.52	3	0
Model 2	−2729.78	−9.02	2	0
Model 3	−2725.27	0	1	1

Abbreviations: *BTI*, marginal likelihood Bezier‐approximated values; LBF, log Bayes factors of the models and their associated probabilities.

### 3.6 Niche Conservatism and Paleodistribution Patterns

**FIGURE 7:**
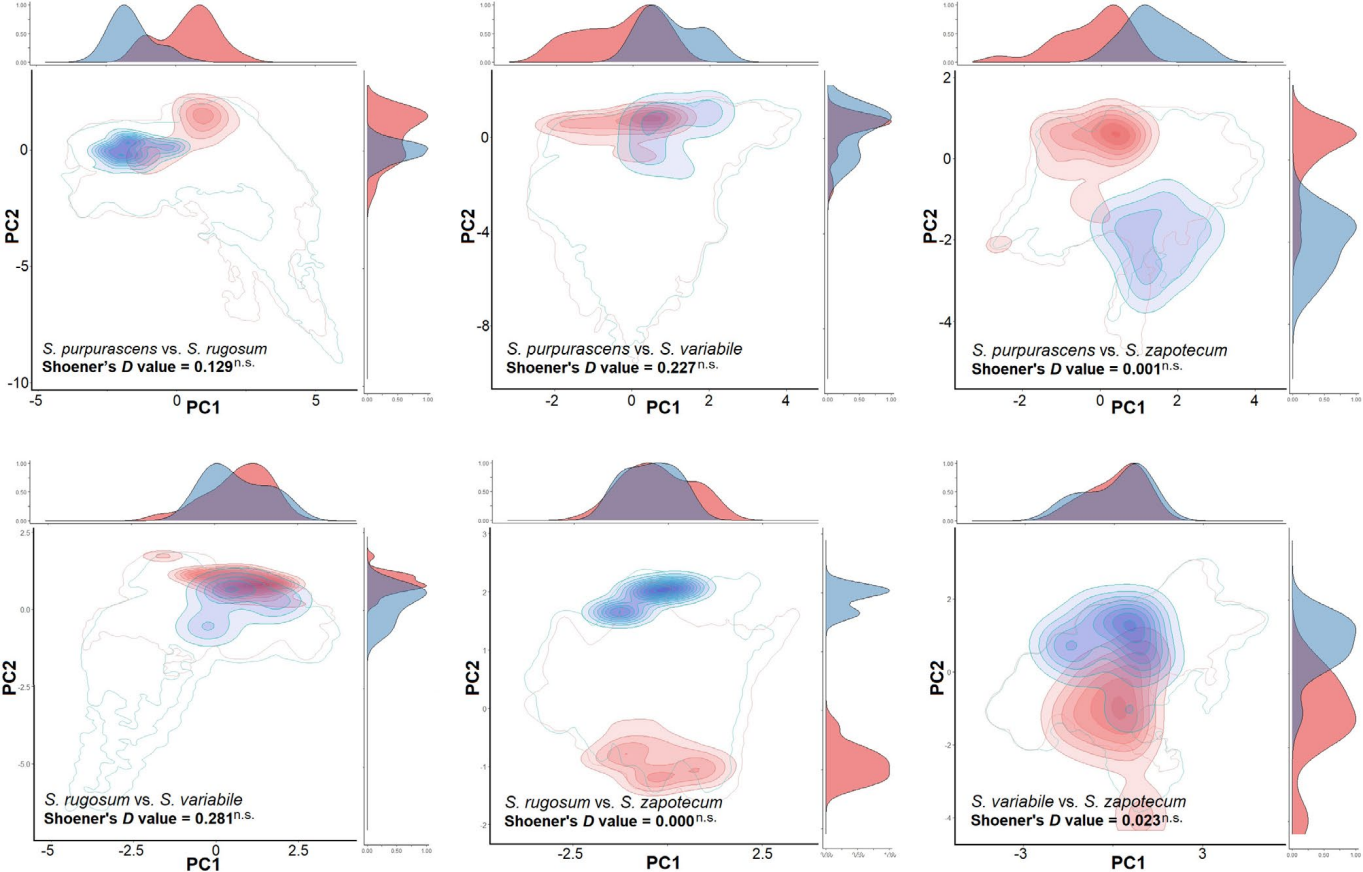
Summary of pairwise comparisons of “climate‐only” niche overlap patterns (based on Schoener's *D* index and equivalency/similarity tests for the four *Sphenarium*' species across the first two axes of the PCA‐env). Red and dark‐red shading represents the kernel density isopleths at different densities (from 10% to 100%) of the niche for species A, while dark‐blue shading represents the same for the species B. The solid lines (red and blue) indicate the available (background) environment for each species. In all plots, the black numbers show the observed values of niche overlap and their significance (ns = nonsignificant [*p* < 0.05]) based on 1000 pseudo‐replicates.

PCA‐Env analyses based on occurrence records revealed significant niche differences among species (Figure 7, Table S6). The first two principal axes explained ~76% (only climatic data) and ~66% (climate‐plus‐habitat) of total variance observed. Niche overlap scores from both approaches indicated that 66.7% of pairwise comparisons showed no or very low overlap (< 0.20), with only two pairs ( *S. purpurascens* vs. *S. variabile* and *S. rugosum* vs. *S. variabile* ) showing low or moderate overlap (> 0.2). Both equivalence and background similarity tests revealed that the niches were not equivalent in any of the comparisons and were not more similar than expected by chance in all climate‐only cases and in 75% of climate‐plus‐habitat cases (see Table S6). These findings generally support the rejection of the niche conservatism hypothesis among species under climate‐only conditions, suggesting that they occupy markedly different environmental conditions.

**FIGURE 8:**
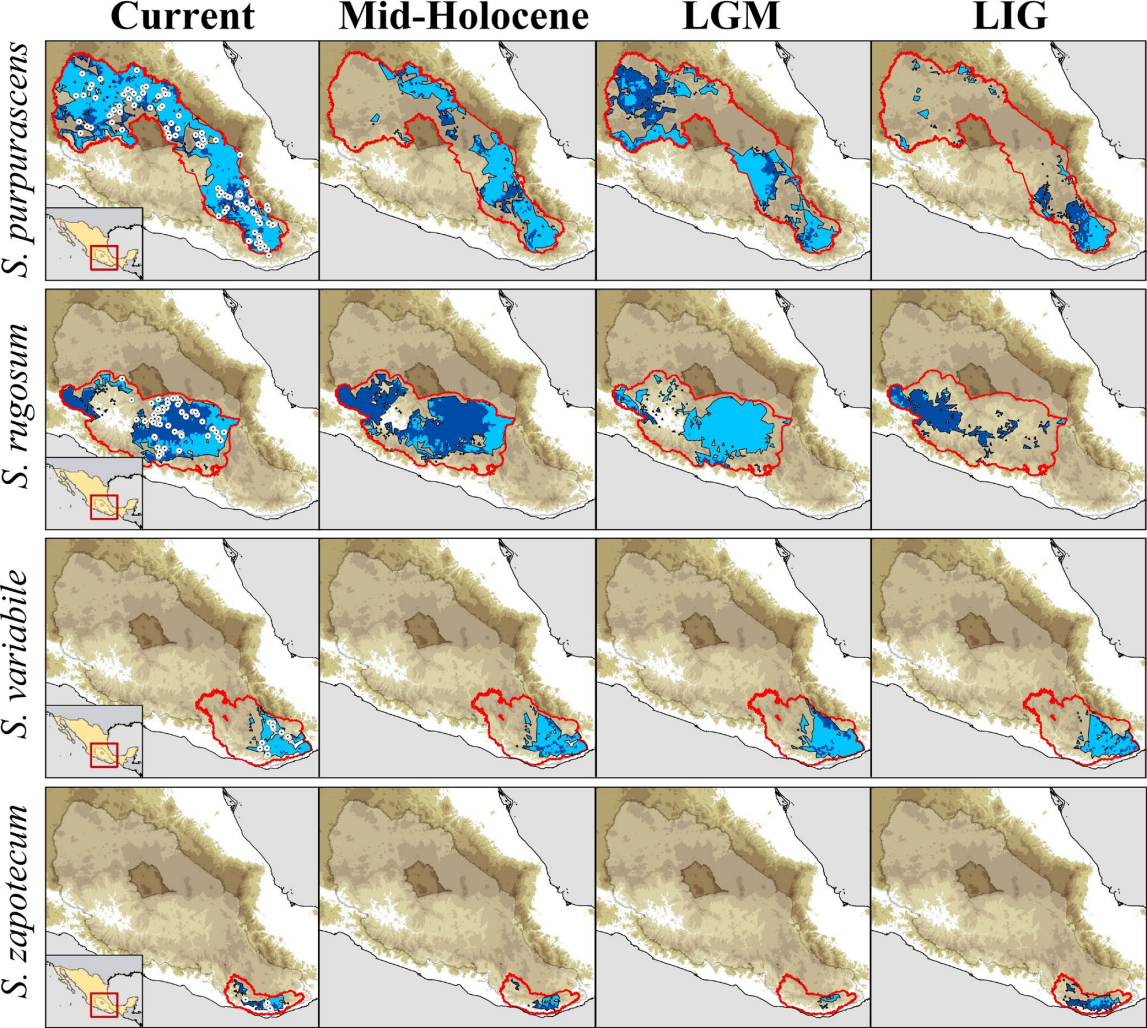
Consensus maps for the Ecological niche model projected onto the geographic areas for the four *Sphenarium*' species during the last glacial and interglacial episodes at Quaternary climate conditions: Mid‐Holocene, Last Glacial Maximum (LGM), and Last Interglacial (LIG). Left maps depict suitability areas for each lineage under Current climatic conditions, showing the sites of historical occurrence records for each species (white points). For all maps, we showed the alloprediction areas among species (lighter blue) and exclusive suitability climatic condition (darker blue) for each species and climate scenario. Models of the Mid‐Holocene and the Last Glacial Maximum (LGM) represent an ensembled projection of three general circulation models used (CCSM4, MIROC, and MPI‐ESM). Red polygon in maps corresponds to the “M” calibration area defined individually for each species, while the lighter white polygon represents the geographical areas considered for past projections and alloprediction analyses.

The distribution models for the four species showed high performance and were better than random expectations (Table S5). Based on these performance metrics, we considered the models reliable for describing species' niches and potential geographic distributions (Figure 8). The smallest estimated geographic range was for *S. zapotecum* (4525 km^2^, representing 28.02% of the calibration area used), while *S. purpurascens* had the largest range extent (121,325 km^2^, 75.21% of the calibration area). *S. rugosum* and *S. variabile* had intermediate ranges of 58,775 km^2^ (52.82% of the calibration area) and 12,975 km^2^ (31.0% of the calibration area), respectively. Additionally, the Jackknife test and variable contributions from MaxEnt revealed interspecific differences in the key environmental variables influencing species distributions (Figure 8): *S. purpurascens* was mainly limited by PREC (18.2%), PDQ (16.7%), MTCQ (16.3%), and PDM (16.3%); *S. rugosum* was influenced primarily by PREC (34.2%), PDM (16.1%), and PDQ (14.5%); *S. variabile* was constrained by MDR (59.44%), PDQ (17.63%), and PDM (12.01%); and *S. zapotecum* was mostly limited by PSE (50.98%), PREC (45.17%), and PDM (3.85%).

Consistent with the observed niche differentiation, geographic overlap among species was minimal. The majority of the analyzed landscape (ca. 99,000 km^2^, 46.72% of the study area) was predicted to be suitable for only one *Sphenarium* species, while 96,575 km^2^ (45.61%) were defined as suitable for at least two species. Besides, only 16,200 km^2^ (7.65% of the total area predicted as suitable for individual species) corresponded to sites predicted to be suitable for at least three species (Figure 8), and only 50 km^2^ (0.02% of the study area) was estimated to be suitable for the co‐occurrence of all four species. Species‐specific exclusive areas (represented by dark colors in Figure 8) ranged from 7.3% (for *S. variabile* ) to 61.46% (for *S. rugosum* ) for total species' area, with *S. purpurascens* and *S. zapotecum* having exclusive ranges of 26.85% and 60.77%, respectively.

Paleodistribution models (Figure 8) suggested changes in the extent, connectivity, and location of potential ranges of the four species since the Late Pleistocene. Overall, we identified several patterns: (i) geographic overlap among models was low, with most suitable areas predicted for a single species (mid‐Holocene = 70.04%; LGM = 45.31%; and LIG = 69.41%) or for at least two species (mid‐Holocene = 23.14%; LGM = 40.33%; and LIG = 26.72%); (ii) the highest values of alloprediction (i.e., spatial predictions of species A under species B's model) occurred during the LGM (81.0% ± 15.0%), with lower niche overlap during the LIG (46.5% ± 31.9%) and mid‐Holocene (58.5% ± 27.0%) compared to the present (60.9% ± 26.6%); (iii) for *S. variabile* , larger and more continuous suitable areas were predicted during the LGM and LIG (~30% more than current); (iv) suitable areas for *S. zapotecum* and *S. rugosum* expanded post‐LGM (32% and 150%, respectively), while *S. purpurascens* expanded only after the LIG, experiencing a contraction during the mid‐Holocene; (v) *S. zapotecum* had smaller and more fragmented suitable areas during the LGM; (vi) predicted climatic stability areas since the Late Pleistocene covered only 10.9%–17.65% of the current distributions of *S. purpurascens* , *S. zapotecum*, and *S. rugosum* ; and (vii) in contrast, stable areas for *S. variabile* represented 90.56% of its current distribution. Notably, climatic stability areas were primarily located in lowland regions across species' geographic ranges. Finally, mean MOP analyses for the mid‐Holocene and LGM periods (Figure S1) indicated that non‐analog climatic conditions do not strongly affect our interpretation of the models. However, during the LIG, a high proportion of the study area (> 50%) exhibited strict extrapolation and non‐analogous climates for all species, which may limit the reliability of model predictions for this period.

## 4 Discussion

This study explored the phylogeographic history of four species of *Sphenarium* grasshopper using a broader mitochondrial dataset and more detailed paleoclimatic models than previous research. We evaluated the predictions of the Glacial and Interglacial Refugia models to assess how Pleistocene climate changes influenced the evolution of these species. Notably, our findings indicate that neither refugial model fully explains the observed genetic and paleodistribution patterns. While the phylogeographic and genetic structure of *S. purpurascens* , *S. rugosum* , and *S. variabile* are consistent with the Glacial refugia model, the paleoclimatic reconstructions and historical demography support the Interglacial model for *S. purpurascens* and *S. variabile* and the Glacial model for *S. rugosum* and *S. zapotecum*. As discussed below, the observed patterns likely result from the combined effects of Pleistocene climate shifts, the complex topography of Mexico, and the limited dispersal abilities of these flightless grasshoppers.

Pleistocene climatic fluctuations probably shaped the genetic diversity of *Sphenarium* species. In particular, the higher genetic diversity observed in *S. purpurascens* populations from the Mexican Volcanic Belt and Sierra Madre del Sur, compared to those from the Mexican Plateau, supports the hypothesis that ancestral populations persisted in climatically stable areas and retained greater genetic diversity than those in recently colonized, less stable environments (Gavin et al. 2014; Loera et al. 2017). Our paleoclimatic models suggest that ancestral and refugial populations of *S. purpurascens* persisted in the lower‐elevation regions of the Mexican Volcanic Belt and the Sierra Madre del Sur, while the northern highlands of the Mexican Plateau were colonized more recently. This interpretation is consistent with our historical migration analyses, which indicate a south‐to‐north colonization pattern (model 3; Figure 3), in which population P2 (Mexican Plateau) diverged from P3 (Trans‐Mexican Volcanic Belt). Although similar patterns of genetic diversity were not observed for the other species, the paleoclimatic reconstructions also indicate past range contractions toward lower elevations, suggesting that lowland refugia likely existed within each species' distribution. Consistent with this, the most differentiated haplotypes of *S. rugosum* (which are indicative of large and stable population size; Avise 2000) were also found in the lowlands of the Balsas Basin.

In *S. purpurascens* , *S. variabile* , and *S. rugosum* , we observed significant population and phylogeographic structure, suggesting historical isolation among their populations. This isolation likely resulted from past range shifts, Mexico's complex orography, and the grasshoppers' limited dispersal capacity. The distribution of these species is commonly interrupted by multiple low‐elevation basins and mountain peaks (Sanabria‐Urbán et al. 2017; see also Figures 2 and 8), which likely act as geographical barriers. For example, in the Sierra Madre del Sur, populations P7 and P9 of *S. purpurascens* exhibit high genetic differentiation (*F*_ST_ = 0.69) despite being separated by just 46 km of linear distance. However, the intervening mountains exceed 3000 m a.s.l., which is higher than the known altitudinal range of *Sphenarium* species (Sanabria‐Urbán et al. 2017). Similar genetic discontinuities associated with orography have been found in other taxa across the Balsas River Basin, Trans‐Mexican Volcanic Belt, and Sierra Madre del Sur, including insects (De‐la‐Mora et al. 2015; Moo‐Llanes et al. 2019), frogs (Bryson et al. 2014), and plants (Fukunaga et al. 2005; Hufford et al. 2012; Gamez et al. 2014).

Geographic distance also appears to contribute significantly to the genetic structure of *S. purpurascens* and *S. variabile* , as expected for species with limited movement (Meirmans 2012). This effect may have been intensified by recent range expansions, as suggested by our paleoclimatic models (G. M. Hewitt 2004; Excoffier et al. 2009). Such expansions could have led to secondary contact between previously isolated populations (Godfrey M. Hewitt 2001), explaining the lack of clear geographic barriers among some genetically distinct populations (e.g., P4–P5 in *S. purpurascens* , V2–V3 in *S. variabile* ). Despite their low dispersal ability, six geographically distant localities of *S. purpurascens* (separated by up to 560 km apart) comprising a single genetic group (population P1) shared the most common haplotypes (h3 and h2), suggesting extensive historical gene flow. Paleodistribution analyses support this, indicating that *S. purpurascens* and *S. variabile* had broader distributions during the LGM, which could have facilitated increased gene flow, as reported in other co‐distributed taxa (Pérez‐Crespo et al. 2017; Rodríguez‐Gómez et al. 2021). Additionally, *S. purpurascens* population P1 spans the Mexican Volcanic Belt and the Sierra Madre del Sur regions (Figure 2), which are recognized as key corridors connecting central and southern Mexican montane biotas (Bryson et al. 2014; Peñaloza‐Ramírez et al. 2020; Ruiz‐Sanchez et al. 2012). Nevertheless, the presence of unique haplotypes in each region suggests limited current gene flow. Furthermore, the retention of ancestral polymorphisms, associated with the recent divergence of mitochondrial lineages for this species (Figure 4), could also explain haplotype sharing between distant populations (Posada and Crandall 2001; Hobolth et al. 2011; Zhou et al. 2017).

On the other hand, rainfall emerged as a key limiting factor shaping the distribution of *Sphenarium* species, as noted by previous studies (Sanabria‐Urbán et al. 2015; Sanabria‐Urbán and del Castillo 2020). Accordingly, historical variations in precipitation have likely been a significant factor influencing range shifts among species. Paleoenvironmental data from Mexico, though limited and covering mainly the last 70,000 years, suggest substantial regional climatic variability (Metcalfe et al. 2000; Metcalfe 2006; Sedov et al. 2009; Ramírez‐Barahona and Eguiarte 2013). This climatic heterogeneity may account for the differing range dynamics observed among species. For instance, wetter conditions persisted in the central Mexican highlands during the LGM, followed by drier Mid‐Holocene conditions (~5000 years ago) (Metcalfe et al. 2000; Metcalfe and Davies 2007; Sedov et al. 2009). These precipitation shifts likely facilitated range expansions of *S. purpurascens* and *S. variabile* during the LGM, followed by contraction in the Mid‐Holocene. Conversely, the drier conditions during the LGM and subsequent wetter periods in tropical lowlands and mid‐elevations (Piperno et al. 2007; Ramírez‐Barahona and Eguiarte 2013) likely reduced habitat suitability for *S. rugosum* and *S. zapotecum* during the LGM, with range expansions occurring from the mid‐Holocene to the present.

We found partial congruence between changes in potential distributions, demographic inferences, and genetic patterns. For *S. rugosum* , BSP analyses suggest a population decline during the LGM followed by continuous expansion beginning in the Holocene. This species also shows high overall nucleotide diversity (*π* = 0.013) and haplotype diversity (Hd = 0.89) consistent with demographic recovery and stability after a population decline during the LGM (Grant and Bowen 1998). In contrast, *S. zapotecum* showed a decline in population size since ~50 kya, as inferred from BSP analyses, despite the expansion of suitable climatic areas during the Mid‐Holocene and present. This result is consistent with the negative Tajima's *D* value and the limited genetic diversity (Hd = 0.42; *π* = 0.0005) of this species, suggesting a recent bottleneck (Tajima 1989) and low effective population size, possibly due to restricted refugial range or delayed demographic recovery (G. M. Hewitt 2004). However, caution is warranted, as an apparent decrease in effective population size may also result from recent secondary contact between previously isolated populations (Wakeley 1999).

For *S. variabile* , neither BSP nor neutrality analyses detected significant population size changes, suggesting demographic stability over time. This is consistent with our finding that about 90% of the species' current distribution remained climatically stable throughout the Late Pleistocene and aligns with the relatively high haplotype diversity (Hd = 0.87) of this species. For *S. purpurascens* , BSP analyses of the most widespread population (P1) and of the pooled dataset revealed demographic expansion concurrent with the increase in suitable areas during the LGM. Interestingly, this expansion persisted despite a reduction in suitable habitat during the Mid‐Holocene (Figure 8). A faster decline in geographic range compared to population size may explain this pattern (e.g., Rodríguez 2002). Populations at the range periphery, which tend to be small and fragmented, are more vulnerable to environmental changes and extinction, whereas core populations in climatically stable areas are more resilient (Caughley and Gunn 1996; Gavin et al. 2014). This idea aligns with our findings that climatically stable areas for *S. purpurascens* persisted in the Sierra Madre del Sur and the Mexican Volcanic Belt (Figure 8), where populations also exhibit high genetic diversity (Table 1), suggesting historically large and stable population sizes (Avise 2000). Moreover, the high haplotype and nucleotide diversity (Hd = 0.89; π = 0.005) of population P1 align with expectations under a model of historical expansion from climatically stable refugia (Excoffier et al. 2009). In contrast, population P2 of *S. purpurascens* , which likely represents a more recent colonization of the Mexican Plateau, shows low diversity (Hd = 0.30; *π* = 0.0003), suggesting a founder effect or bottleneck during post‐glacial range shifts.

Increases in population size in *S. purpurascens* and *S. rugosum* toward the present may also be associated with human‐induced environmental changes. Over the past 3000 years, human‐induced fires, deforestation, and land cultivation have intensified across central Mexico (Metcalfe and Davies 2007; Sedov et al. 2009) and in the Balsas River Basin (Piperno et al. 2007). These changes may have favored *Sphenarium* grasshoppers, which thrive in open areas (Sanabria‐Urbán and del Castillo 2020) and have been reported as agricultural pests since pre‐Columbian times (Ramos‐Elorduy and Moreno 1989).

Our mtDNA analyses revealed that none of the four *Sphenarium* species is monophyletic. However, monophyly in single‐locus gene trees is not a necessary criterion for species delimitation, particularly in recently diverged or reticulated groups (de Queiroz 2007; Ross 2014). Moreover, the species included in our study are ecologically distinct, morphologically diagnosable (Sanabria‐Urbán et al. 2017), and supported by genomic data (Benites et al. 2023) as independently evolving lineages. These multiple lines of evidence justify their recognition as separate species. The lack of monophyly may reflect retention of ancestral polymorphism due to recent divergence and reticulated evolutionary history due to interbreeding among these species (Pedraza‐Lara et al. 2015; Sanabria‐Urbán et al. 2015, 2017; Benites et al. 2023). Recent work by Benites et al. (2023) documents extensive introgressive hybridization, particularly mitochondrial introgression from *S. purpurascens* to *S. variabile* and *S. zapotecum*. This is consistent with the haplotype sharing we observed in the Sierra Madre del Sur, where these species' ranges overlap (Figures 2 and 4), as well as with previous reports of interspecific mating in nature and captivity (Sanabria‐Urbán et al. 2017). Nevertheless, only 3 out of the 74 haplotypes identified in our study were shared among three species, and these cases were geographically restricted to contact zones (Figure 2). These results suggest that hybridization is not widespread between *S. purpurascens* , *S. zapotecum*, and *S. variabile* , but instead reflect limited and spatially structured gene flow, consistent with both ongoing and secondary contact following past range changes (Funk and Omland 2003).

Our findings further suggest that hybridization events have occurred repeatedly throughout their evolutionary history, likely facilitated by demographic and range shifts. For example, we identified the same haplogroups in overlapping (H and J) and distant (C and F) populations of these three species in the Sierra Madre del Sur (see Figures 2 and 4), likely indicating both recent and more ancient hybridization events, respectively. The LGM range expansions of *S. purpurascens* and *S. variabile* may have created low‐elevation corridors, facilitating ancient hybridization with *S. zapotecum*, while more recent interbreeding may have followed Holocene expansions of *S. purpurascens* . Overall, changes in range size and location over time (Figure 8) likely promoted these interactions, highlighting the complex evolutionary history of this group.

The causes behind the extensive and unidirectional introgression remain unclear. Benites et al. (2023) proposed that selective backcrossing might play a role in *Sphenarium*, but cytonuclear incompatibilities may also favor unidirectional mitochondrial introgression, as occurs in other insects (e.g., Emerson and Glaser 2017). Introgression has the potential to facilitate adaptation to novel environments or enhance the fitness of hybrids (Hedrick 2013). Nevertheless, its role in adaptation must be confirmed experimentally. Notably, the introgression of neutral loci, including in mtDNA, does not necessarily compromise species integrity, particularly when strong selective pressures preserve divergence in crucial ecological traits (e.g., Feder et al. 2012; Dowle et al. 2014). Future studies using genome‐wide neutral and functional nuclear markers will be crucial to disentangle the role of natural selection, distinguish recent from ancient introgression, quantify gene flow directionality, and identify genomic regions with elevated introgression (e.g., Edelman et al. 2019).

Our results also highlight how local adaptation to environmental variation could drive evolutionary divergence. During the Late Pleistocene, shifts in species' potential distributions likely reduced ancestral niche availability, driving adaptation to new conditions. Similar recent niche differentiation patterns are noted in taxa from regions like the Mexican Volcanic Belt (Hidalgo‐Licona et al. 2023) and Sierra Madre del Sur (Moreno‐Contreras et al. 2020). This ecological differentiation enables the coexistence of closely related species, such as *S. purpurascens* , *S. variabile* , and *S. zapotecum*, within the Sierra Madre del Sur. These findings underscore the importance of ecological adaptation in maintaining species identity despite gene flow and suggest potential research pathways that include vegetation structure and land‐use factors to improve niche characterizations.

This study is among the few phylogeographic analyses of Mexican grasshoppers to integrate genetic data with paleoclimatic information. However, using a single mitochondrial marker has inherent limitations, as it traces only the maternal lineage and may be affected by factors like introgression or incomplete lineage sorting (Heller et al. 2013; Rodríguez‐Gómez et al. 2021). Mitochondrial data offers valuable insights into species' evolutionary history (Bryson et al. 2010), but its limitations warrant careful interpretation of phylogenetic and demographic patterns. We also recognize that this type of data alone does not allow fine‐scale temporal resolution (on the order of a few thousand years), and the lack of species monophyly may inflate estimates of effective population size and gene flow (Petit and Excoffier 2009; Knowles and Carstens 2007). While our findings align with previously described biogeographic and ecological patterns, they represent conservative estimates. Future studies integrating genome‐wide nuclear markers and larger sample sizes are necessary to conduct robust model‐based statistical comparisons and refine the phylogeographic and demographic inferences presented here.

## 5 Conclusions

Phylogeographic and paleoclimatic analyses suggest that Pleistocene climate changes significantly influenced the differentiation of mitochondrial lineages in *Sphenarium* species. The interspecific divergences, distribution, and demographic changes in the four species studied appear to closely coincide with these past climatic events. However, the species responded differently to these climatic fluctuations, and neither of the refugial models fully explained the observed patterns. It is likely that low‐elevation regions within each species' distribution ranges functioned as refugia during the Pleistocene, maintaining suitable climatic conditions and, in some cases, preserving high levels of genetic diversity. Additionally, the distribution range and demographic changes observed in *S. purpurascens* , *S. variabile* , and *S. zapotecum* may have facilitated multiple hybridization events among them over the last 120,000 years. Our findings underscore the importance of local adaptation and ecological niche divergence as key drivers of speciation in *Sphenarium*, providing new insights into the evolutionary history of this diverse Neotropical grasshopper lineage. Despite the inherent limitation of using a single mitochondrial marker for phylogeographic inferences, this study represents a foundation for future work incorporating additional nuclear markers and genomic data.

## Author Contributions

**Salomón Sanabria‐Urbán:** conceptualization (equal), data curation (equal), formal analysis (equal), investigation (equal), methodology (equal), writing – original draft (equal), writing – review and editing (equal). **Andrés Torres‐Miranda:** data curation (equal), formal analysis (equal), methodology (equal), writing – original draft (equal), writing – review and editing (equal). **David A. Prieto‐Torres:** data curation (equal), formal analysis (equal), methodology (equal), writing – original draft (equal), writing – review and editing (equal). **Ken Oyama:** conceptualization (equal), funding acquisition (equal), investigation (equal), supervision (equal), writing – review and editing (equal). **Raúl Cueva del Castillo:** conceptualization (equal), funding acquisition (equal), investigation (equal), project administration (equal), supervision (equal), writing – review and editing (equal).

## Conflicts of Interest

The authors declare no conflicts of interest.

## Supporting information


**Data S1:** ece372209‐sup‐0001‐Supinfo.docx.

## Data Availability

All sequences generated and analyzed during the current study are available in the GenBank (https://www.ncbi.nlm.nih.gov/genbank/) throughout the following accession numbers: PP409674–PP409970. Geographic records analyzed during this study are referenced in this article and are publicly available in its Supporting Information file. All scripts and input information are available in https://github.com/davidprietorres/Sphenarium_paleodistribuion.
