# Supplementary material for: Pleistocene Climate Changes and Lineage Diversification of Sphenarium Grasshoppers (Orthoptera: Pyrgomorphidae)
Source: Ecol Evol. 2025 Oct 10;15(10):e72209. doi: 10.1002/ece3.72209 (PMC12511763; doi:10.1002/ece3.72209)

**Pleistocene climate changes and lineage diversification of *Sphenarium* Grasshoppers (Orthoptera: Pyrgomorphidae)**

Salomón Sanabria-Urbán^1^, Andrés Torres-Miranda^2^, David A. Prieto-Torres^3,4^, Ken Oyama^2^, & Raúl Cueva del Castillo^1*^

^1^Laboratorio de Ecología, UBIPRO, Facultad de Estudios Superiores Iztacala, Universidad Nacional Autónoma de México (UNAM), A.P. 314, Tlalnepantla, 54090 México, México

^2^ Escuela Nacional de Estudios Superiores (ENES) Unidad Morelia, UNAM, Morelia, 58190 Michoacán, México.

^3^ Laboratorio de Biodiversidad y Cambio Global (LABIOCG); Facultad de Estudios Superiores Iztacala, Universidad Nacional Autónoma de México, Tlalnepantla, Estado de México, México.

^4^Laboratorio Nacional CONAHCyT de Biología del Cambio Climático, Ciudad de México, México

*Corresponding Author: [rcueva@ecologia.unam.mx](mailto:rcueva@ecologia.unam.mx)

**Supplementary information**

**Supplementary information file 1**

**Table S1**. List of specimens used for the genetic analyses including taxonomic identification, sampling locality description, geographic coordinates, and number (LN), as well as voucher number indicating sex (f and m, for females and males, respectively) and GenBank accession numbers for CO1sequences of each individual.

| **Species** | **Locality descriotion** | **Latitude** | **Longitude** | **LN** | **Individual voucher** | **GB Accesion** |
| --- | --- | --- | --- | --- | --- | --- |
| *Sphenarium purpurascens* | Mexico, Michoacan, Pasando Sta. Clara del Cobre, Carr120, L367, 2/11/2010, Coll. Sanabria-Urbán S. & Cueva del Castillo R. | 19.424361 | -101.60483 | 1 | PU-13S-14f | PP409674 |
| *Sphenarium purpurascens* | Mexico, Michoacan, Pasando Sta. Clara del Cobre, Carr120, L367, 2/11/2010, Coll. Sanabria-Urbán S. & Cueva del Castillo R. | 19.424361 | -101.60483 | 1 | PU-13S-15f | PP409675 |
| *Sphenarium purpurascens* | Mexico, Michoacan, Pasando Sta. Clara del Cobre, Carr120, L367, 2/11/2010, Coll. Sanabria-Urbán S. & Cueva del Castillo R. | 19.424361 | -101.60483 | 1 | PU-13S-16f | PP409676 |
| *Sphenarium purpurascens* | Mexico, Michoacan, Pasando Sta. Clara del Cobre, Carr120, L367, 2/11/2010, Coll. Sanabria-Urbán S. & Cueva del Castillo R. | 19.424361 | -101.60483 | 1 | PU-13S-1m | PP409677 |
| *Sphenarium purpurascens* | Mexico, Michoacan, Pasando Sta. Clara del Cobre, Carr120, L367, 2/11/2010, Coll. Sanabria-Urbán S. & Cueva del Castillo R. | 19.424361 | -101.60483 | 1 | PU-13S-3m | PP409678 |
| *Sphenarium purpurascens* | Mexico, Michoacan, Pasando Sta. Clara del Cobre, Carr120, L367, 2/11/2010, Coll. Sanabria-Urbán S. & Cueva del Castillo R. | 19.424361 | -101.60483 | 1 | PU-13S-5m | PP409679 |
| *Sphenarium purpurascens* | Mexico, Michoacan, Pasando Sta. Clara del Cobre, Carr120, L367, 2/11/2010, Coll. Sanabria-Urbán S. & Cueva del Castillo R. | 19.424361 | -101.60483 | 1 | PU-13S-7m | PP409680 |
| *Sphenarium purpurascens* | Mexico, Oaxaca, Entre Huajuapan y Tamazulapan Carr 190, L33, 28/10/2011, Coll. Sanabria-Urban S. & Díaz de la Vega A. | 17.721682 | -97.666333 | 2 | PU-35C-11f | PP409681 |
| *Sphenarium purpurascens* | Mexico, Oaxaca, Entre Huajuapan y Tamazulapan Carr 190, L33, 28/10/2011, Coll. Sanabria-Urban S. & Díaz de la Vega A. | 17.721682 | -97.666333 | 2 | PU-35C-12f | PP409682 |
| *Sphenarium purpurascens* | Mexico, Oaxaca, Entre Huajuapan y Tamazulapan Carr 190, L33, 28/10/2011, Coll. Sanabria-Urban S. & Díaz de la Vega A. | 17.721682 | -97.666333 | 2 | PU-35C-13f | PP409683 |
| *Sphenarium purpurascens* | Mexico, Oaxaca, Entre Huajuapan y Tamazulapan Carr 190, L33, 28/10/2011, Coll. Sanabria-Urban S. & Díaz de la Vega A. | 17.721682 | -97.666333 | 2 | PU-35C-3m | PP409684 |
| *Sphenarium purpurascens* | Mexico, Oaxaca, Entre Huajuapan y Tamazulapan Carr 190, L33, 28/10/2011, Coll. Sanabria-Urban S. & Díaz de la Vega A. | 17.721682 | -97.666333 | 2 | PU-35C-4m | PP409685 |
| *Sphenarium purpurascens* | Mexico, Oaxaca, Entre Huajuapan y Tamazulapan Carr 190, L33, 28/10/2011, Coll. Sanabria-Urban S. & Díaz de la Vega A. | 17.721682 | -97.666333 | 2 | PU-35C-7m | PP409686 |
| *Sphenarium purpurascens* | Mexico, Oaxaca, Entre Huajuapan y Tamazulapan Carr 190, L33, 28/10/2011, Coll. Sanabria-Urban S. & Díaz de la Vega A. | 17.721682 | -97.666333 | 2 | PU-35C-8m | PP409687 |
| *Sphenarium purpurascens* | Mexico, Guanajuato , Tarandacuaro, L32, 9/9/2008, Coll. Sanabria-Urbán S. & Cueva del Castillo R. | 20.158167 | -100.54929 | 3 | PU-ACA-10m | PP409688 |
| *Sphenarium purpurascens* | Mexico, Guanajuato , Tarandacuaro, L32, 9/9/2008, Coll. Sanabria-Urbán S. & Cueva del Castillo R. | 20.158167 | -100.54929 | 3 | PU-ACA-11m | PP409689 |
| *Sphenarium purpurascens* | Mexico, Guanajuato , Tarandacuaro, L32, 9/9/2008, Coll. Sanabria-Urbán S. & Cueva del Castillo R. | 20.158167 | -100.54929 | 3 | PU-ACA-12m | PP409690 |
| *Sphenarium purpurascens* | Mexico, Guanajuato , Tarandacuaro, L32, 9/9/2008, Coll. Sanabria-Urbán S. & Cueva del Castillo R. | 20.158167 | -100.54929 | 3 | PU-ACA-13f | PP409691 |
| *Sphenarium purpurascens* | Mexico, Guanajuato , Tarandacuaro, L32, 9/9/2008, Coll. Sanabria-Urbán S. & Cueva del Castillo R. | 20.158167 | -100.54929 | 3 | PU-ACA-1m | PP409692 |
| *Sphenarium purpurascens* | Mexico, Guanajuato , Tarandacuaro, L32, 9/9/2008, Coll. Sanabria-Urbán S. & Cueva del Castillo R. | 20.158167 | -100.54929 | 3 | PU-ACA-2m | PP409693 |
| *Sphenarium purpurascens* | Mexico, Guanajuato , Tarandacuaro, L32, 9/9/2008, Coll. Sanabria-Urbán S. & Cueva del Castillo R. | 20.158167 | -100.54929 | 3 | PU-ACA-3f | PP409694 |
| *Sphenarium purpurascens* | Mexico, Guanajuato , Tarandacuaro, L32, 9/9/2008, Coll. Sanabria-Urbán S. & Cueva del Castillo R. | 20.158167 | -100.54929 | 3 | PU-ACA-4m | PP409695 |
| *Sphenarium purpurascens* | Mexico, Guanajuato , Tarandacuaro, L32, 9/9/2008, Coll. Sanabria-Urbán S. & Cueva del Castillo R. | 20.158167 | -100.54929 | 3 | PU-ACA-5f | PP409696 |
| *Sphenarium purpurascens* | Mexico, Guanajuato , Tarandacuaro, L32, 9/9/2008, Coll. Sanabria-Urbán S. & Cueva del Castillo R. | 20.158167 | -100.54929 | 3 | PU-ACA-6f | PP409697 |
| *Sphenarium purpurascens* | Mexico, Tlaxcala, Atotonilco, L42, 10/9/2008, Coll. Sanabria-Urbán S. & Cueva del Castillo R. | 19.373514 | -98.4653 | 4 | PU-ATO-10m | PP409698 |
| *Sphenarium purpurascens* | Mexico, Tlaxcala, Atotonilco, L42, 10/9/2008, Coll. Sanabria-Urbán S. & Cueva del Castillo R. | 19.373514 | -98.4653 | 4 | PU-ATO-12f | PP409699 |
| *Sphenarium purpurascens* | Mexico, Tlaxcala, Atotonilco, L42, 10/9/2008, Coll. Sanabria-Urbán S. & Cueva del Castillo R. | 19.373514 | -98.4653 | 4 | PU-ATO-13m | PP409700 |
| *Sphenarium purpurascens* | Mexico, Tlaxcala, Atotonilco, L42, 10/9/2008, Coll. Sanabria-Urbán S. & Cueva del Castillo R. | 19.373514 | -98.4653 | 4 | PU-ATO-2f | PP409701 |
| *Sphenarium purpurascens* | Mexico, Tlaxcala, Atotonilco, L42, 10/9/2008, Coll. Sanabria-Urbán S. & Cueva del Castillo R. | 19.373514 | -98.4653 | 4 | PU-ATO-3m | PP409702 |
| *Sphenarium purpurascens* | Mexico, Tlaxcala, Atotonilco, L42, 10/9/2008, Coll. Sanabria-Urbán S. & Cueva del Castillo R. | 19.373514 | -98.4653 | 4 | PU-ATO-4m | PP409703 |
| *Sphenarium purpurascens* | Mexico, Tlaxcala, Atotonilco, L42, 10/9/2008, Coll. Sanabria-Urbán S. & Cueva del Castillo R. | 19.373514 | -98.4653 | 4 | PU-ATO-5f | PP409704 |
| *Sphenarium purpurascens* | Mexico, Tlaxcala, Atotonilco, L42, 10/9/2008, Coll. Sanabria-Urbán S. & Cueva del Castillo R. | 19.373514 | -98.4653 | 4 | PU-ATO-7m | PP409705 |
| *Sphenarium purpurascens* | Mexico, Tlaxcala, Atotonilco, L42, 10/9/2008, Coll. Sanabria-Urbán S. & Cueva del Castillo R. | 19.373514 | -98.4653 | 4 | PU-ATO-8f | PP409706 |
| *Sphenarium purpurascens* | Mexico, Tlaxcala, Atotonilco, L42, 10/9/2008, Coll. Sanabria-Urbán S. & Cueva del Castillo R. | 19.373514 | -98.4653 | 4 | PU-ATO-9m | PP409707 |
| *Sphenarium purpurascens* | Mexico, Puebla, Cacaxtla, L40, 10/9/2008, Coll. Sanabria-Urbán S. & Cueva del Castillo R. | 19.116563 | -98.388752 | 5 | PU-CAX-10m | PP409708 |
| *Sphenarium purpurascens* | Mexico, Puebla, Cacaxtla, L40, 10/9/2008, Coll. Sanabria-Urbán S. & Cueva del Castillo R. | 19.116563 | -98.388752 | 5 | PU-CAX-1f | PP409709 |
| *Sphenarium purpurascens* | Mexico, Puebla, Cacaxtla, L40, 10/9/2008, Coll. Sanabria-Urbán S. & Cueva del Castillo R. | 19.116563 | -98.388752 | 5 | PU-CAX-2m | PP409710 |
| *Sphenarium purpurascens* | Mexico, Puebla, Cacaxtla, L40, 10/9/2008, Coll. Sanabria-Urbán S. & Cueva del Castillo R. | 19.116563 | -98.388752 | 5 | PU-CAX-3m | PP409711 |
| *Sphenarium purpurascens* | Mexico, Puebla, Cacaxtla, L40, 10/9/2008, Coll. Sanabria-Urbán S. & Cueva del Castillo R. | 19.116563 | -98.388752 | 5 | PU-CAX-4m | PP409712 |
| *Sphenarium purpurascens* | Mexico, Puebla, Cacaxtla, L40, 10/9/2008, Coll. Sanabria-Urbán S. & Cueva del Castillo R. | 19.116563 | -98.388752 | 5 | PU-CAX-5m | PP409713 |
| *Sphenarium purpurascens* | Mexico, Puebla, Cacaxtla, L40, 10/9/2008, Coll. Sanabria-Urbán S. & Cueva del Castillo R. | 19.116563 | -98.388752 | 5 | PU-CAX-6f | PP409714 |
| *Sphenarium purpurascens* | Mexico, Puebla, Cacaxtla, L40, 10/9/2008, Coll. Sanabria-Urbán S. & Cueva del Castillo R. | 19.116563 | -98.388752 | 5 | PU-CAX-7f | PP409715 |
| *Sphenarium purpurascens* | Mexico, Puebla, Cacaxtla, L40, 10/9/2008, Coll. Sanabria-Urbán S. & Cueva del Castillo R. | 19.116563 | -98.388752 | 5 | PU-CAX-8m | PP409716 |
| *Sphenarium purpurascens* | Mexico, Puebla, Cacaxtla, L40, 10/9/2008, Coll. Sanabria-Urbán S. & Cueva del Castillo R. | 19.116563 | -98.388752 | 5 | PU-CAX-9m | PP409717 |
| *Sphenarium purpurascens* | Mexico, Puebla, Cholula, L34, 10/9/2008, Coll. Sanabria-Urbán S. & Cueva del Castillo R. | 19.056361 | -98.30425 | 6 | PU-CHO-10m | PP409718 |
| *Sphenarium purpurascens* | Mexico, Puebla, Cholula, L34, 10/9/2008, Coll. Sanabria-Urbán S. & Cueva del Castillo R. | 19.056361 | -98.30425 | 6 | PU-CHO-1f | PP409719 |
| *Sphenarium purpurascens* | Mexico, Puebla, Cholula, L34, 10/9/2008, Coll. Sanabria-Urbán S. & Cueva del Castillo R. | 19.056361 | -98.30425 | 6 | PU-CHO-2f | PP409720 |
| *Sphenarium purpurascens* | Mexico, Puebla, Cholula, L34, 10/9/2008, Coll. Sanabria-Urbán S. & Cueva del Castillo R. | 19.056361 | -98.30425 | 6 | PU-CHO-3f | PP409721 |
| *Sphenarium purpurascens* | Mexico, Puebla, Cholula, L34, 10/9/2008, Coll. Sanabria-Urbán S. & Cueva del Castillo R. | 19.056361 | -98.30425 | 6 | PU-CHO-4m | PP409722 |
| *Sphenarium purpurascens* | Mexico, Puebla, Cholula, L34, 10/9/2008, Coll. Sanabria-Urbán S. & Cueva del Castillo R. | 19.056361 | -98.30425 | 6 | PU-CHO-5m | PP409723 |
| *Sphenarium purpurascens* | Mexico, Puebla, Cholula, L34, 10/9/2008, Coll. Sanabria-Urbán S. & Cueva del Castillo R. | 19.056361 | -98.30425 | 6 | PU-CHO-6m | PP409724 |
| *Sphenarium purpurascens* | Mexico, Puebla, Cholula, L34, 10/9/2008, Coll. Sanabria-Urbán S. & Cueva del Castillo R. | 19.056361 | -98.30425 | 6 | PU-CHO-7m | PP409725 |
| *Sphenarium purpurascens* | Mexico, Puebla, Cholula, L34, 10/9/2008, Coll. Sanabria-Urbán S. & Cueva del Castillo R. | 19.056361 | -98.30425 | 6 | PU-CHO-8f | PP409726 |
| *Sphenarium purpurascens* | Mexico, Puebla, Cholula, L34, 10/9/2008, Coll. Sanabria-Urbán S. & Cueva del Castillo R. | 19.056361 | -98.30425 | 6 | PU-CHO-9m | PP409727 |
| *Sphenarium purpurascens* | Mexico, Mexico, Desviasión a Amealco , L43, 8/9/2008, Coll. Sanabria-Urbán S. & Cueva del Castillo R. | 20.113611 | -99.703056 | 7 | PU-DAM-10f | PP409728 |
| *Sphenarium purpurascens* | Mexico, Mexico, Desviasión a Amealco , L43, 8/9/2008, Coll. Sanabria-Urbán S. & Cueva del Castillo R. | 20.113611 | -99.703056 | 7 | PU-DAM-1m | PP409729 |
| *Sphenarium purpurascens* | Mexico, Mexico, Desviasión a Amealco , L43, 8/9/2008, Coll. Sanabria-Urbán S. & Cueva del Castillo R. | 20.113611 | -99.703056 | 7 | PU-DAM-2m | PP409730 |
| *Sphenarium purpurascens* | Mexico, Mexico, Desviasión a Amealco , L43, 8/9/2008, Coll. Sanabria-Urbán S. & Cueva del Castillo R. | 20.113611 | -99.703056 | 7 | PU-DAM-3m | PP409731 |
| *Sphenarium purpurascens* | Mexico, Mexico, Desviasión a Amealco , L43, 8/9/2008, Coll. Sanabria-Urbán S. & Cueva del Castillo R. | 20.113611 | -99.703056 | 7 | PU-DAM-4f | PP409732 |
| *Sphenarium purpurascens* | Mexico, Mexico, Desviasión a Amealco , L43, 8/9/2008, Coll. Sanabria-Urbán S. & Cueva del Castillo R. | 20.113611 | -99.703056 | 7 | PU-DAM-5m | PP409733 |
| *Sphenarium purpurascens* | Mexico, Mexico, Desviasión a Amealco , L43, 8/9/2008, Coll. Sanabria-Urbán S. & Cueva del Castillo R. | 20.113611 | -99.703056 | 7 | PU-DAM-6f | PP409734 |
| *Sphenarium purpurascens* | Mexico, Mexico, Desviasión a Amealco , L43, 8/9/2008, Coll. Sanabria-Urbán S. & Cueva del Castillo R. | 20.113611 | -99.703056 | 7 | PU-DAM-7f | PP409735 |
| *Sphenarium purpurascens* | Mexico, Mexico, Desviasión a Amealco , L43, 8/9/2008, Coll. Sanabria-Urbán S. & Cueva del Castillo R. | 20.113611 | -99.703056 | 7 | PU-DAM-8f | PP409736 |
| *Sphenarium purpurascens* | Mexico, Mexico, Desviasión a Amealco , L43, 8/9/2008, Coll. Sanabria-Urbán S. & Cueva del Castillo R. | 20.113611 | -99.703056 | 7 | PU-DAM-9f | PP409737 |
| *Sphenarium purpurascens* | Mexico, Guanajuato , Rumbo a Guanajuato, L35, 9/9/2008, Coll. Sanabria-Urbán S. & Cueva del Castillo R. | 19.938042 | -100.76991 | 8 | PU-GUA-10f | PP409738 |
| *Sphenarium purpurascens* | Mexico, Guanajuato , Rumbo a Guanajuato, L35, 9/9/2008, Coll. Sanabria-Urbán S. & Cueva del Castillo R. | 19.938042 | -100.76991 | 8 | PU-GUA-1m | PP409739 |
| *Sphenarium purpurascens* | Mexico, Guanajuato , Rumbo a Guanajuato, L35, 9/9/2008, Coll. Sanabria-Urbán S. & Cueva del Castillo R. | 19.938042 | -100.76991 | 8 | PU-GUA-2m | PP409740 |
| *Sphenarium purpurascens* | Mexico, Guanajuato , Rumbo a Guanajuato, L35, 9/9/2008, Coll. Sanabria-Urbán S. & Cueva del Castillo R. | 19.938042 | -100.76991 | 8 | PU-GUA-3f | PP409741 |
| *Sphenarium purpurascens* | Mexico, Guanajuato , Rumbo a Guanajuato, L35, 9/9/2008, Coll. Sanabria-Urbán S. & Cueva del Castillo R. | 19.938042 | -100.76991 | 8 | PU-GUA-4m | PP409742 |
| *Sphenarium purpurascens* | Mexico, Guanajuato , Rumbo a Guanajuato, L35, 9/9/2008, Coll. Sanabria-Urbán S. & Cueva del Castillo R. | 19.938042 | -100.76991 | 8 | PU-GUA-5m | PP409743 |
| *Sphenarium purpurascens* | Mexico, Guanajuato , Rumbo a Guanajuato, L35, 9/9/2008, Coll. Sanabria-Urbán S. & Cueva del Castillo R. | 19.938042 | -100.76991 | 8 | PU-GUA-6m | PP409744 |
| *Sphenarium purpurascens* | Mexico, Guanajuato , Rumbo a Guanajuato, L35, 9/9/2008, Coll. Sanabria-Urbán S. & Cueva del Castillo R. | 19.938042 | -100.76991 | 8 | PU-GUA-7f | PP409745 |
| *Sphenarium purpurascens* | Mexico, Guanajuato , Rumbo a Guanajuato, L35, 9/9/2008, Coll. Sanabria-Urbán S. & Cueva del Castillo R. | 19.938042 | -100.76991 | 8 | PU-GUA-8m | PP409746 |
| *Sphenarium purpurascens* | Mexico, Guanajuato , Rumbo a Guanajuato, L35, 9/9/2008, Coll. Sanabria-Urbán S. & Cueva del Castillo R. | 19.938042 | -100.76991 | 8 | PU-GUA-9m | PP409747 |
| *Sphenarium purpurascens* | Mexico, Oaxaca, Carr  175 Km 87 , L61, 13/12/2013, Coll. Sanabria-Urbán S., P. Fontana & Mariño-Pérez R. | 16.3862211 | -96.656034 | 9 | PU-L36-10m | PP409748 |
| *Sphenarium purpurascens* | Mexico, Oaxaca, Carr  175 Km 87 , L61, 13/12/2013, Coll. Sanabria-Urbán S., P. Fontana & Mariño-Pérez R. | 16.3862211 | -96.656034 | 9 | PU-L36-11m | PP409749 |
| *Sphenarium purpurascens* | Mexico, Oaxaca, Carr  175 Km 87 , L61, 13/12/2013, Coll. Sanabria-Urbán S., P. Fontana & Mariño-Pérez R. | 16.3862211 | -96.656034 | 9 | PU-L36-12m | PP409750 |
| *Sphenarium purpurascens* | Mexico, Oaxaca, Carr  175 Km 87 , L61, 13/12/2013, Coll. Sanabria-Urbán S., P. Fontana & Mariño-Pérez R. | 16.3862211 | -96.656034 | 9 | PU-L36-13m | PP409751 |
| *Sphenarium purpurascens* | Mexico, Oaxaca, Carr  175 Km 87 , L61, 13/12/2013, Coll. Sanabria-Urbán S., P. Fontana & Mariño-Pérez R. | 16.3862211 | -96.656034 | 9 | PU-L36-14m | PP409752 |
| *Sphenarium purpurascens* | Mexico, Oaxaca, Carr  175 Km 87 , L61, 13/12/2013, Coll. Sanabria-Urbán S., P. Fontana & Mariño-Pérez R. | 16.3862211 | -96.656034 | 9 | PU-L36-15m | PP409753 |
| *Sphenarium purpurascens* | Mexico, Oaxaca, Carr  175 Km 87 , L61, 13/12/2013, Coll. Sanabria-Urbán S., P. Fontana & Mariño-Pérez R. | 16.3862211 | -96.656034 | 9 | PU-L36-16m | PP409754 |
| *Sphenarium purpurascens* | Mexico, Oaxaca, El Capulín , L600, 14/12/2013, Coll. Sanabria-Urbán S., P. Fontana & Mariño-Pérez R. | 17.5168276 | -96.94341 | 10 | PU-L37-10f | PP409755 |
| *Sphenarium purpurascens* | Mexico, Oaxaca, El Capulín , L600, 14/12/2013, Coll. Sanabria-Urbán S., P. Fontana & Mariño-Pérez R. | 17.5168276 | -96.94341 | 10 | PU-L37-11f | PP409756 |
| *Sphenarium purpurascens* | Mexico, Oaxaca, El Capulín , L600, 14/12/2013, Coll. Sanabria-Urbán S., P. Fontana & Mariño-Pérez R. | 17.5168276 | -96.94341 | 10 | PU-L37-2m | PP409757 |
| *Sphenarium purpurascens* | Mexico, Oaxaca, El Capulín , L600, 14/12/2013, Coll. Sanabria-Urbán S., P. Fontana & Mariño-Pérez R. | 17.5168276 | -96.94341 | 10 | PU-L37-3m | PP409758 |
| *Sphenarium purpurascens* | Mexico, Oaxaca, El Capulín , L600, 14/12/2013, Coll. Sanabria-Urbán S., P. Fontana & Mariño-Pérez R. | 17.5168276 | -96.94341 | 10 | PU-L37-4m | PP409759 |
| *Sphenarium purpurascens* | Mexico, Oaxaca, El Capulín , L600, 14/12/2013, Coll. Sanabria-Urbán S., P. Fontana & Mariño-Pérez R. | 17.5168276 | -96.94341 | 10 | PU-L37-5m | PP409760 |
| *Sphenarium purpurascens* | Mexico, Oaxaca, El Capulín , L600, 14/12/2013, Coll. Sanabria-Urbán S., P. Fontana & Mariño-Pérez R. | 17.5168276 | -96.94341 | 10 | PU-L37-6m | PP409761 |
| *Sphenarium purpurascens* | Mexico, Oaxaca, El Capulín , L600, 14/12/2013, Coll. Sanabria-Urbán S., P. Fontana & Mariño-Pérez R. | 17.5168276 | -96.94341 | 10 | PU-L37-7f | PP409762 |
| *Sphenarium purpurascens* | Mexico, Oaxaca, El Capulín , L600, 14/12/2013, Coll. Sanabria-Urbán S., P. Fontana & Mariño-Pérez R. | 17.5168276 | -96.94341 | 10 | PU-L37-8f | PP409763 |
| *Sphenarium purpurascens* | Mexico, Oaxaca, El Capulín , L600, 14/12/2013, Coll. Sanabria-Urbán S., P. Fontana & Mariño-Pérez R. | 17.5168276 | -96.94341 | 10 | PU-L37-9f | PP409764 |
| *Sphenarium purpurascens* | Mexico, Oaxaca, Rumbo a Teotitlan Carr 131 Km 71 , L25, 14/12/2013, Coll. Sanabria-Urbán S., P. Fontana & Mariño-Pérez R. | 18.0804474 | -97.064835 | 11 | PU-L39-10f | PP409765 |
| *Sphenarium purpurascens* | Mexico, Oaxaca, Rumbo a Teotitlan Carr 131 Km 71 , L25, 14/12/2013, Coll. Sanabria-Urbán S., P. Fontana & Mariño-Pérez R. | 18.0804474 | -97.064835 | 11 | PU-L39-1m | PP409766 |
| *Sphenarium purpurascens* | Mexico, Oaxaca, Rumbo a Teotitlan Carr 131 Km 71 , L25, 14/12/2013, Coll. Sanabria-Urbán S., P. Fontana & Mariño-Pérez R. | 18.0804474 | -97.064835 | 11 | PU-L39-2m | PP409767 |
| *Sphenarium purpurascens* | Mexico, Oaxaca, Rumbo a Teotitlan Carr 131 Km 71 , L25, 14/12/2013, Coll. Sanabria-Urbán S., P. Fontana & Mariño-Pérez R. | 18.0804474 | -97.064835 | 11 | PU-L39-4m | PP409768 |
| *Sphenarium purpurascens* | Mexico, Oaxaca, Rumbo a Teotitlan Carr 131 Km 71 , L25, 14/12/2013, Coll. Sanabria-Urbán S., P. Fontana & Mariño-Pérez R. | 18.0804474 | -97.064835 | 11 | PU-L39-5m | PP409769 |
| *Sphenarium purpurascens* | Mexico, Oaxaca, Rumbo a Teotitlan Carr 131 Km 71 , L25, 14/12/2013, Coll. Sanabria-Urbán S., P. Fontana & Mariño-Pérez R. | 18.0804474 | -97.064835 | 11 | PU-L39-6f | PP409770 |
| *Sphenarium purpurascens* | Mexico, Oaxaca, Rumbo a Teotitlan Carr 131 Km 71 , L25, 14/12/2013, Coll. Sanabria-Urbán S., P. Fontana & Mariño-Pérez R. | 18.0804474 | -97.064835 | 11 | PU-L39-7f | PP409771 |
| *Sphenarium purpurascens* | Mexico, Oaxaca, Rumbo a Teotitlan Carr 131 Km 71 , L25, 14/12/2013, Coll. Sanabria-Urbán S., P. Fontana & Mariño-Pérez R. | 18.0804474 | -97.064835 | 11 | PU-L39-8f | PP409772 |
| *Sphenarium purpurascens* | Mexico, Oaxaca, Rumbo a Teotitlan Carr 131 Km 71 , L25, 14/12/2013, Coll. Sanabria-Urbán S., P. Fontana & Mariño-Pérez R. | 18.0804474 | -97.064835 | 11 | PU-L39-9m | PP409773 |
| *Sphenarium purpurascens* | México, Oaxaca, Ixtlán , L80, 19/10/2015, Coll. Sanabria-Urbán S. & Jiménez-Arcos V.H. | 17.321142 | -96.484014 | 12 | PU-L40-1m | PP409774 |
| *Sphenarium purpurascens* | México, Oaxaca, Ixtlán , L80, 19/10/2015, Coll. Sanabria-Urbán S. & Jiménez-Arcos V.H. | 17.321142 | -96.484014 | 12 | PU-L40-3m | PP409775 |
| *Sphenarium purpurascens* | México, Oaxaca, Ixtlán , L80, 19/10/2015, Coll. Sanabria-Urbán S. & Jiménez-Arcos V.H. | 17.321142 | -96.484014 | 12 | PU-L40-4m | PP409776 |
| *Sphenarium purpurascens* | México, Oaxaca, Ixtlán , L80, 19/10/2015, Coll. Sanabria-Urbán S. & Jiménez-Arcos V.H. | 17.321142 | -96.484014 | 12 | PU-L40-5f | PP409777 |
| *Sphenarium purpurascens* | México, Oaxaca, Ixtlán , L80, 19/10/2015, Coll. Sanabria-Urbán S. & Jiménez-Arcos V.H. | 17.321142 | -96.484014 | 12 | PU-L40-6f | PP409778 |
| *Sphenarium purpurascens* | México, Oaxaca, Ixtlán , L80, 19/10/2015, Coll. Sanabria-Urbán S. & Jiménez-Arcos V.H. | 17.321142 | -96.484014 | 12 | PU-L40-8f | PP409779 |
| *Sphenarium purpurascens* | México, Oaxaca, Ixtlán , L80, 19/10/2015, Coll. Sanabria-Urbán S. & Jiménez-Arcos V.H. | 17.321142 | -96.484014 | 12 | PU-L40-9f | PP409780 |
| *Sphenarium purpurascens* | México, Oaxaca, Carr. 125, ca. 8Km NE de Tlaxiaco, L81, 19/10/2015, Coll. Sanabria-Urbán S. & Jiménez-Arcos V.H. | 17.329305 | -97.63512 | 13 | PU-L41-1m | PP409781 |
| *Sphenarium purpurascens* | México, Oaxaca, Carr. 125, ca. 8Km NE de Tlaxiaco, L81, 19/10/2015, Coll. Sanabria-Urbán S. & Jiménez-Arcos V.H. | 17.329305 | -97.63512 | 13 | PU-L41-2m | PP409782 |
| *Sphenarium purpurascens* | México, Oaxaca, Carr. 125, ca. 8Km NE de Tlaxiaco, L81, 19/10/2015, Coll. Sanabria-Urbán S. & Jiménez-Arcos V.H. | 17.329305 | -97.63512 | 13 | PU-L41-3m | PP409783 |
| *Sphenarium purpurascens* | México, Oaxaca, Carr. 125, ca. 8Km NE de Tlaxiaco, L81, 19/10/2015, Coll. Sanabria-Urbán S. & Jiménez-Arcos V.H. | 17.329305 | -97.63512 | 13 | PU-L41-4f | PP409784 |
| *Sphenarium purpurascens* | México, Oaxaca, Carr. 125, ca. 8Km NE de Tlaxiaco, L81, 19/10/2015, Coll. Sanabria-Urbán S. & Jiménez-Arcos V.H. | 17.329305 | -97.63512 | 13 | PU-L41-5f | PP409785 |
| *Sphenarium purpurascens* | México, Oaxaca, Carr. 125, ca. 8Km NE de Tlaxiaco, L81, 19/10/2015, Coll. Sanabria-Urbán S. & Jiménez-Arcos V.H. | 17.329305 | -97.63512 | 13 | PU-L41-6f | PP409786 |
| *Sphenarium purpurascens* | México, Oaxaca, Carr. 125, ca. 8Km NE de Tlaxiaco, L81, 19/10/2015, Coll. Sanabria-Urbán S. & Jiménez-Arcos V.H. | 17.329305 | -97.63512 | 13 | PU-L41-7f | PP409787 |
| *Sphenarium purpurascens* | Mexico, Oaxaca, Monte Alban, L28, 4/10/2008, Coll. Sanabria-Urbán S. & Pingarroni A. | 17.047051 | -96.764276 | 14 | PU-MOA-1f | PP409788 |
| *Sphenarium purpurascens* | Mexico, Oaxaca, Monte Alban, L28, 4/10/2008, Coll. Sanabria-Urbán S. & Pingarroni A. | 17.047051 | -96.764276 | 14 | PU-MOA-2f | PP409789 |
| *Sphenarium purpurascens* | Mexico, Oaxaca, Monte Alban, L28, 4/10/2008, Coll. Sanabria-Urbán S. & Pingarroni A. | 17.047051 | -96.764276 | 14 | PU-MOA-3m | PP409790 |
| *Sphenarium purpurascens* | Mexico, Oaxaca, Monte Alban, L28, 4/10/2008, Coll. Sanabria-Urbán S. & Pingarroni A. | 17.047051 | -96.764276 | 14 | PU-MOA-4m | PP409791 |
| *Sphenarium purpurascens* | Mexico, Oaxaca, Monte Alban, L28, 4/10/2008, Coll. Sanabria-Urbán S. & Pingarroni A. | 17.047051 | -96.764276 | 14 | PU-MOA-5f | PP409792 |
| *Sphenarium purpurascens* | Mexico, Oaxaca, Monte Alban, L28, 4/10/2008, Coll. Sanabria-Urbán S. & Pingarroni A. | 17.047051 | -96.764276 | 14 | PU-MOA-6f | PP409793 |
| *Sphenarium purpurascens* | Mexico, Oaxaca, Monte Alban, L28, 4/10/2008, Coll. Sanabria-Urbán S. & Pingarroni A. | 17.047051 | -96.764276 | 14 | PU-MOA-7m | PP409794 |
| *Sphenarium purpurascens* | Mexico, Oaxaca, Monte Alban, L28, 4/10/2008, Coll. Sanabria-Urbán S. & Pingarroni A. | 17.047051 | -96.764276 | 14 | PU-MOA-8m | PP409795 |
| *Sphenarium purpurascens* | Mexico, Oaxaca, Monte Alban, L28, 4/10/2008, Coll. Sanabria-Urbán S. & Pingarroni A. | 17.047051 | -96.764276 | 14 | PU-MOA-9f | PP409796 |
| *Sphenarium purpurascens* | Mexico, Oaxaca, Monte Alban, L28, 4/10/2008, Coll. Sanabria-Urbán S. & Pingarroni A. | 17.047051 | -96.764276 | 14 | PU-MOA-Bm | PP409797 |
| *Sphenarium purpurascens* | Mexico, Oaxaca, Entrada Oaxaca, L27, 12/9/2008, Coll. Sanabria-Urbán S. & Cueva del Castillo R. | 17.1436111 | -96.815833 | 15 | PU-OAX-1m | PP409798 |
| *Sphenarium purpurascens* | Mexico, Oaxaca, Entrada Oaxaca, L27, 12/9/2008, Coll. Sanabria-Urbán S. & Cueva del Castillo R. | 17.1436111 | -96.815833 | 15 | PU-OAX-20f | PP409799 |
| *Sphenarium purpurascens* | Mexico, Oaxaca, Entrada Oaxaca, L27, 12/9/2008, Coll. Sanabria-Urbán S. & Cueva del Castillo R. | 17.1436111 | -96.815833 | 15 | PU-OAX-26f | PP409800 |
| *Sphenarium purpurascens* | Mexico, Oaxaca, Entrada Oaxaca, L27, 12/9/2008, Coll. Sanabria-Urbán S. & Cueva del Castillo R. | 17.1436111 | -96.815833 | 15 | PU-OAX-30m | PP409801 |
| *Sphenarium purpurascens* | Mexico, Oaxaca, Entrada Oaxaca, L27, 12/9/2008, Coll. Sanabria-Urbán S. & Cueva del Castillo R. | 17.1436111 | -96.815833 | 15 | PU-OAX-31m | PP409802 |
| *Sphenarium purpurascens* | Mexico, Oaxaca, Entrada Oaxaca, L27, 12/9/2008, Coll. Sanabria-Urbán S. & Cueva del Castillo R. | 17.1436111 | -96.815833 | 15 | PU-OAX-36m | PP409803 |
| *Sphenarium purpurascens* | Mexico, Oaxaca, Entrada Oaxaca, L27, 12/9/2008, Coll. Sanabria-Urbán S. & Cueva del Castillo R. | 17.1436111 | -96.815833 | 15 | PU-OAX-40m | PP409804 |
| *Sphenarium purpurascens* | Mexico, Oaxaca, Entrada Oaxaca, L27, 12/9/2008, Coll. Sanabria-Urbán S. & Cueva del Castillo R. | 17.1436111 | -96.815833 | 15 | PU-OAX-44m | PP409805 |
| *Sphenarium purpurascens* | Mexico, Oaxaca, Entrada Oaxaca, L27, 12/9/2008, Coll. Sanabria-Urbán S. & Cueva del Castillo R. | 17.1436111 | -96.815833 | 15 | PU-OAX-5m | PP409806 |
| *Sphenarium purpurascens* | Mexico, Veracruz , Cumbres de Maltrata Carr 150D, L29, 25/9/2012, Coll. Sanabria-Urban & Rivera-Ortiz F. | 18.827865 | -97.254168 | 16 | PU-P45-11f | PP409807 |
| *Sphenarium purpurascens* | Mexico, Veracruz , Cumbres de Maltrata Carr 150D, L29, 25/9/2012, Coll. Sanabria-Urban & Rivera-Ortiz F. | 18.827865 | -97.254168 | 16 | PU-P45-12f | PP409808 |
| *Sphenarium purpurascens* | Mexico, Veracruz , Cumbres de Maltrata Carr 150D, L29, 25/9/2012, Coll. Sanabria-Urban & Rivera-Ortiz F. | 18.827865 | -97.254168 | 16 | PU-P45-13f | PP409809 |
| *Sphenarium purpurascens* | Mexico, Veracruz , Cumbres de Maltrata Carr 150D, L29, 25/9/2012, Coll. Sanabria-Urban & Rivera-Ortiz F. | 18.827865 | -97.254168 | 16 | PU-P45-2m | PP409810 |
| *Sphenarium purpurascens* | Mexico, Veracruz , Cumbres de Maltrata Carr 150D, L29, 25/9/2012, Coll. Sanabria-Urban & Rivera-Ortiz F. | 18.827865 | -97.254168 | 16 | PU-P45-3m | PP409811 |
| *Sphenarium purpurascens* | Mexico, Veracruz , Cumbres de Maltrata Carr 150D, L29, 25/9/2012, Coll. Sanabria-Urban & Rivera-Ortiz F. | 18.827865 | -97.254168 | 16 | PU-P45-5m | PP409812 |
| *Sphenarium purpurascens* | Mexico, Veracruz , Cumbres de Maltrata Carr 150D, L29, 25/9/2012, Coll. Sanabria-Urban & Rivera-Ortiz F. | 18.827865 | -97.254168 | 16 | PU-P45-6m | PP409813 |
| *Sphenarium purpurascens* | Mexico, Veracruz , Cumbres de Maltrata Carr 150D, L29, 25/9/2012, Coll. Sanabria-Urban & Rivera-Ortiz F. | 18.827865 | -97.254168 | 16 | PU-P45-8m | PP409814 |
| *Sphenarium purpurascens* | Mexico, Oaxaca, San Adres Paxtlan Carr 175 km 116, L39, 2/10/2012, Coll. Sanabria-Urban & Rivera-Ortiz F. | 16.241674 | -96.52472 | 17 | PU-P56-1m | PP409815 |
| *Sphenarium purpurascens* | Mexico, Oaxaca, San Adres Paxtlan Carr 175 km 116, L39, 2/10/2012, Coll. Sanabria-Urban & Rivera-Ortiz F. | 16.241674 | -96.52472 | 17 | PU-P56-4m | PP409816 |
| *Sphenarium purpurascens* | Mexico, Oaxaca, San Adres Paxtlan Carr 175 km 116, L39, 2/10/2012, Coll. Sanabria-Urban & Rivera-Ortiz F. | 16.241674 | -96.52472 | 17 | PU-P56-5m | PP409817 |
| *Sphenarium purpurascens* | Mexico, Oaxaca, San Adres Paxtlan Carr 175 km 116, L39, 2/10/2012, Coll. Sanabria-Urban & Rivera-Ortiz F. | 16.241674 | -96.52472 | 17 | PU-P56-6m | PP409818 |
| *Sphenarium purpurascens* | Mexico, Oaxaca, San Adres Paxtlan Carr 175 km 116, L39, 2/10/2012, Coll. Sanabria-Urban & Rivera-Ortiz F. | 16.241674 | -96.52472 | 17 | PU-P56-8m | PP409819 |
| *Sphenarium purpurascens* | Mexico, Oaxaca, Rumbo a Hierve el Agua, L94, 3/10/2012, Coll. Sanabria-Urban & Rivera-Ortiz F. | 16.934178 | -96.317295 | 18 | PU-P59-2m | PP409820 |
| *Sphenarium purpurascens* | Mexico, Oaxaca, Rumbo a Hierve el Agua, L94, 3/10/2012, Coll. Sanabria-Urban & Rivera-Ortiz F. | 16.934178 | -96.317295 | 18 | PU-P59-3m | PP409821 |
| *Sphenarium purpurascens* | Mexico, Oaxaca, Rumbo a Hierve el Agua, L94, 3/10/2012, Coll. Sanabria-Urban & Rivera-Ortiz F. | 16.934178 | -96.317295 | 18 | PU-P59-4m | PP409822 |
| *Sphenarium purpurascens* | Mexico, Oaxaca, Rumbo a Hierve el Agua, L94, 3/10/2012, Coll. Sanabria-Urban & Rivera-Ortiz F. | 16.934178 | -96.317295 | 18 | PU-P59-5m | PP409823 |
| *Sphenarium purpurascens* | Mexico, Oaxaca, Rumbo a Hierve el Agua, L94, 3/10/2012, Coll. Sanabria-Urban & Rivera-Ortiz F. | 16.934178 | -96.317295 | 18 | PU-P59-6m | PP409824 |
| *Sphenarium purpurascens* | Mexico, Oaxaca, Rumbo a Hierve el Agua, L94, 3/10/2012, Coll. Sanabria-Urban & Rivera-Ortiz F. | 16.934178 | -96.317295 | 18 | PU-P59-7m | PP409825 |
| *Sphenarium purpurascens* | Mexico, Oaxaca, Rumbo a Hierve el Agua, L94, 3/10/2012, Coll. Sanabria-Urban & Rivera-Ortiz F. | 16.934178 | -96.317295 | 18 | PU-P59-8m | PP409826 |
| *Sphenarium purpurascens* | Mexico, Oaxaca, Rumbo a Hierve el Agua, L94, 3/10/2012, Coll. Sanabria-Urban & Rivera-Ortiz F. | 16.934178 | -96.317295 | 18 | PU-P59-9m | PP409827 |
| *Sphenarium purpurascens* | Mexico, Guanajuato , León , L30, 5/10/2013, Coll. Sanabria-Urban & Rivera-Ortiz F. | 21.0415404 | -101.56044 | 19 | PU-P83-15f | PP409828 |
| *Sphenarium purpurascens* | Mexico, Guanajuato , León , L30, 5/10/2013, Coll. Sanabria-Urban & Rivera-Ortiz F. | 21.0415404 | -101.56044 | 19 | PU-P83-16f | PP409829 |
| *Sphenarium purpurascens* | Mexico, Guanajuato , León , L30, 5/10/2013, Coll. Sanabria-Urban & Rivera-Ortiz F. | 21.0415404 | -101.56044 | 19 | PU-P83-18f | PP409830 |
| *Sphenarium purpurascens* | Mexico, Guanajuato , León , L30, 5/10/2013, Coll. Sanabria-Urban & Rivera-Ortiz F. | 21.0415404 | -101.56044 | 19 | PU-P83-3m | PP409831 |
| *Sphenarium purpurascens* | Mexico, Guanajuato , León , L30, 5/10/2013, Coll. Sanabria-Urban & Rivera-Ortiz F. | 21.0415404 | -101.56044 | 19 | PU-P83-4m | PP409832 |
| *Sphenarium purpurascens* | Mexico, Guanajuato , León , L30, 5/10/2013, Coll. Sanabria-Urban & Rivera-Ortiz F. | 21.0415404 | -101.56044 | 19 | PU-P83-5m | PP409833 |
| *Sphenarium purpurascens* | Mexico, Guanajuato , León , L30, 5/10/2013, Coll. Sanabria-Urban & Rivera-Ortiz F. | 21.0415404 | -101.56044 | 19 | PU-P83-6m | PP409834 |
| *Sphenarium purpurascens* | Mexico, Guanajuato , León , L30, 5/10/2013, Coll. Sanabria-Urban & Rivera-Ortiz F. | 21.0415404 | -101.56044 | 19 | PU-P83-7m | PP409835 |
| *Sphenarium purpurascens* | Mexico, Guanajuato , León , L30, 5/10/2013, Coll. Sanabria-Urban & Rivera-Ortiz F. | 21.0415404 | -101.56044 | 19 | PU-P83-8m | PP409836 |
| *Sphenarium purpurascens* | Mexico, Guanajuato , Sta Catarina , L26, 6/10/2013, Coll. Sanabria-Urban & Rivera-Ortiz F. | 21.1361692 | -100.06848 | 20 | PU-P84-10f | PP409837 |
| *Sphenarium purpurascens* | Mexico, Guanajuato , Sta Catarina , L26, 6/10/2013, Coll. Sanabria-Urban & Rivera-Ortiz F. | 21.1361692 | -100.06848 | 20 | PU-P84-2m | PP409838 |
| *Sphenarium purpurascens* | Mexico, Guanajuato , Sta Catarina , L26, 6/10/2013, Coll. Sanabria-Urban & Rivera-Ortiz F. | 21.1361692 | -100.06848 | 20 | PU-P84-3m | PP409839 |
| *Sphenarium purpurascens* | Mexico, Guanajuato , Sta Catarina , L26, 6/10/2013, Coll. Sanabria-Urban & Rivera-Ortiz F. | 21.1361692 | -100.06848 | 20 | PU-P84-4f | PP409840 |
| *Sphenarium purpurascens* | Mexico, Guanajuato , Sta Catarina , L26, 6/10/2013, Coll. Sanabria-Urban & Rivera-Ortiz F. | 21.1361692 | -100.06848 | 20 | PU-P84-7f | PP409841 |
| *Sphenarium purpurascens* | Mexico, Guanajuato , Sta Catarina , L26, 6/10/2013, Coll. Sanabria-Urban & Rivera-Ortiz F. | 21.1361692 | -100.06848 | 20 | PU-P84-8f | PP409842 |
| *Sphenarium purpurascens* | Mexico, Hidalgo, Carr 85 entre Tasquillo y Aguacatal, L379, 6/10/2013, Coll. Sanabria-Urban & Rivera-Ortiz F. | 20.6010751 | -99.339313 | 21 | PU-P85-10m | PP409843 |
| *Sphenarium purpurascens* | Mexico, Hidalgo, Carr 85 entre Tasquillo y Aguacatal, L379, 6/10/2013, Coll. Sanabria-Urban & Rivera-Ortiz F. | 20.6010751 | -99.339313 | 21 | PU-P85-11f | PP409844 |
| *Sphenarium purpurascens* | Mexico, Hidalgo, Carr 85 entre Tasquillo y Aguacatal, L379, 6/10/2013, Coll. Sanabria-Urban & Rivera-Ortiz F. | 20.6010751 | -99.339313 | 21 | PU-P85-13f | PP409845 |
| *Sphenarium purpurascens* | Mexico, Hidalgo, Carr 85 entre Tasquillo y Aguacatal, L379, 6/10/2013, Coll. Sanabria-Urban & Rivera-Ortiz F. | 20.6010751 | -99.339313 | 21 | PU-P85-1m | PP409846 |
| *Sphenarium purpurascens* | Mexico, Hidalgo, Carr 85 entre Tasquillo y Aguacatal, L379, 6/10/2013, Coll. Sanabria-Urban & Rivera-Ortiz F. | 20.6010751 | -99.339313 | 21 | PU-P85-2m | PP409847 |
| *Sphenarium purpurascens* | Mexico, Hidalgo, Carr 85 entre Tasquillo y Aguacatal, L379, 6/10/2013, Coll. Sanabria-Urban & Rivera-Ortiz F. | 20.6010751 | -99.339313 | 21 | PU-P85-3m | PP409848 |
| *Sphenarium purpurascens* | Mexico, Hidalgo, Carr 85 entre Tasquillo y Aguacatal, L379, 6/10/2013, Coll. Sanabria-Urban & Rivera-Ortiz F. | 20.6010751 | -99.339313 | 21 | PU-P85-5m | PP409849 |
| *Sphenarium purpurascens* | Mexico, Hidalgo, Carr 85 entre Tasquillo y Aguacatal, L379, 6/10/2013, Coll. Sanabria-Urban & Rivera-Ortiz F. | 20.6010751 | -99.339313 | 21 | PU-P85-6m | PP409850 |
| *Sphenarium purpurascens* | Mexico, Tlaxcala, Papalotla, L37, 11/9/2008, Coll. Sanabria-Urbán S. & Cueva del Castillo R. | 19.162208 | -98.223497 | 22 | PU-PAP-10m | PP409851 |
| *Sphenarium purpurascens* | Mexico, Tlaxcala, Papalotla, L37, 11/9/2008, Coll. Sanabria-Urbán S. & Cueva del Castillo R. | 19.162208 | -98.223497 | 22 | PU-PAP-1m | PP409852 |
| *Sphenarium purpurascens* | Mexico, Tlaxcala, Papalotla, L37, 11/9/2008, Coll. Sanabria-Urbán S. & Cueva del Castillo R. | 19.162208 | -98.223497 | 22 | PU-PAP-2m | PP409853 |
| *Sphenarium purpurascens* | Mexico, Tlaxcala, Papalotla, L37, 11/9/2008, Coll. Sanabria-Urbán S. & Cueva del Castillo R. | 19.162208 | -98.223497 | 22 | PU-PAP-3m | PP409854 |
| *Sphenarium purpurascens* | Mexico, Tlaxcala, Papalotla, L37, 11/9/2008, Coll. Sanabria-Urbán S. & Cueva del Castillo R. | 19.162208 | -98.223497 | 22 | PU-PAP-4m | PP409855 |
| *Sphenarium purpurascens* | Mexico, Tlaxcala, Papalotla, L37, 11/9/2008, Coll. Sanabria-Urbán S. & Cueva del Castillo R. | 19.162208 | -98.223497 | 22 | PU-PAP-5m | PP409856 |
| *Sphenarium purpurascens* | Mexico, Tlaxcala, Papalotla, L37, 11/9/2008, Coll. Sanabria-Urbán S. & Cueva del Castillo R. | 19.162208 | -98.223497 | 22 | PU-PAP-6m | PP409857 |
| *Sphenarium purpurascens* | Mexico, Tlaxcala, Papalotla, L37, 11/9/2008, Coll. Sanabria-Urbán S. & Cueva del Castillo R. | 19.162208 | -98.223497 | 22 | PU-PAP-7m | PP409858 |
| *Sphenarium purpurascens* | Mexico, Tlaxcala, Papalotla, L37, 11/9/2008, Coll. Sanabria-Urbán S. & Cueva del Castillo R. | 19.162208 | -98.223497 | 22 | PU-PAP-8f | PP409859 |
| *Sphenarium purpurascens* | Mexico, Tlaxcala, Papalotla, L37, 11/9/2008, Coll. Sanabria-Urbán S. & Cueva del Castillo R. | 19.162208 | -98.223497 | 22 | PU-PAP-9m | PP409860 |
| *Sphenarium purpurascens* | Mexico, Queretaro, El Paraíso, Peña de Bernal, L31, 8/9/2008, Coll. Sanabria-Urbán S. & Cueva del Castillo R. | 20.564156 | -100.21541 | 23 | PU-PEB-10f | PP409861 |
| *Sphenarium purpurascens* | Mexico, Queretaro, El Paraíso, Peña de Bernal, L31, 8/9/2008, Coll. Sanabria-Urbán S. & Cueva del Castillo R. | 20.564156 | -100.21541 | 23 | PU-PEB-1m | PP409862 |
| *Sphenarium purpurascens* | Mexico, Queretaro, El Paraíso, Peña de Bernal, L31, 8/9/2008, Coll. Sanabria-Urbán S. & Cueva del Castillo R. | 20.564156 | -100.21541 | 23 | PU-PEB-2f | PP409863 |
| *Sphenarium purpurascens* | Mexico, Queretaro, El Paraíso, Peña de Bernal, L31, 8/9/2008, Coll. Sanabria-Urbán S. & Cueva del Castillo R. | 20.564156 | -100.21541 | 23 | PU-PEB-3f | PP409864 |
| *Sphenarium purpurascens* | Mexico, Queretaro, El Paraíso, Peña de Bernal, L31, 8/9/2008, Coll. Sanabria-Urbán S. & Cueva del Castillo R. | 20.564156 | -100.21541 | 23 | PU-PEB-4m | PP409865 |
| *Sphenarium purpurascens* | Mexico, Queretaro, El Paraíso, Peña de Bernal, L31, 8/9/2008, Coll. Sanabria-Urbán S. & Cueva del Castillo R. | 20.564156 | -100.21541 | 23 | PU-PEB-5m | PP409866 |
| *Sphenarium purpurascens* | Mexico, Queretaro, El Paraíso, Peña de Bernal, L31, 8/9/2008, Coll. Sanabria-Urbán S. & Cueva del Castillo R. | 20.564156 | -100.21541 | 23 | PU-PEB-6f | PP409867 |
| *Sphenarium purpurascens* | Mexico, Queretaro, El Paraíso, Peña de Bernal, L31, 8/9/2008, Coll. Sanabria-Urbán S. & Cueva del Castillo R. | 20.564156 | -100.21541 | 23 | PU-PEB-7m | PP409868 |
| *Sphenarium purpurascens* | Mexico, Queretaro, El Paraíso, Peña de Bernal, L31, 8/9/2008, Coll. Sanabria-Urbán S. & Cueva del Castillo R. | 20.564156 | -100.21541 | 23 | PU-PEB-8m | PP409869 |
| *Sphenarium purpurascens* | Mexico, Queretaro, El Paraíso, Peña de Bernal, L31, 8/9/2008, Coll. Sanabria-Urbán S. & Cueva del Castillo R. | 20.564156 | -100.21541 | 23 | PU-PEB-9m | PP409870 |
| *Sphenarium purpurascens* | Mexico, Ciudad de México , Pedregal de San Ángel, L41, 5/10/2008, Coll. Sanabria-Urbán S. & Cueva del Castillo R. | 19.3333333 | -99.133333 | 24 | PU-PED-11m | PP409871 |
| *Sphenarium purpurascens* | Mexico, Ciudad de México , Pedregal de San Ángel, L41, 5/10/2008, Coll. Sanabria-Urbán S. & Cueva del Castillo R. | 19.3333333 | -99.133333 | 24 | PU-PED-12f | PP409872 |
| *Sphenarium purpurascens* | Mexico, Ciudad de México , Pedregal de San Ángel, L41, 5/10/2008, Coll. Sanabria-Urbán S. & Cueva del Castillo R. | 19.3333333 | -99.133333 | 24 | PU-PED-13f | PP409873 |
| *Sphenarium purpurascens* | Mexico, Ciudad de México , Pedregal de San Ángel, L41, 5/10/2008, Coll. Sanabria-Urbán S. & Cueva del Castillo R. | 19.3333333 | -99.133333 | 24 | PU-PED-14f | PP409874 |
| *Sphenarium purpurascens* | Mexico, Ciudad de México , Pedregal de San Ángel, L41, 5/10/2008, Coll. Sanabria-Urbán S. & Cueva del Castillo R. | 19.3333333 | -99.133333 | 24 | PU-PED-15m | PP409875 |
| *Sphenarium purpurascens* | Mexico, Ciudad de México , Pedregal de San Ángel, L41, 5/10/2008, Coll. Sanabria-Urbán S. & Cueva del Castillo R. | 19.3333333 | -99.133333 | 24 | PU-PED-18m | PP409876 |
| *Sphenarium purpurascens* | Mexico, Ciudad de México , Pedregal de San Ángel, L41, 5/10/2008, Coll. Sanabria-Urbán S. & Cueva del Castillo R. | 19.3333333 | -99.133333 | 24 | PU-PED-21m | PP409877 |
| *Sphenarium purpurascens* | Mexico, Ciudad de México , Pedregal de San Ángel, L41, 5/10/2008, Coll. Sanabria-Urbán S. & Cueva del Castillo R. | 19.3333333 | -99.133333 | 24 | PU-PED-22f | PP409878 |
| *Sphenarium purpurascens* | Mexico, Ciudad de México , Pedregal de San Ángel, L41, 5/10/2008, Coll. Sanabria-Urbán S. & Cueva del Castillo R. | 19.3333333 | -99.133333 | 24 | PU-PED-24f | PP409879 |
| *Sphenarium purpurascens* | Mexico, Ciudad de México , Pedregal de San Ángel, L41, 5/10/2008, Coll. Sanabria-Urbán S. & Cueva del Castillo R. | 19.3333333 | -99.133333 | 24 | PU-PED-25f | PP409880 |
| *Sphenarium purpurascens* | Mexico, Tlaxcala, Techachalco, L38, 11/9/2008, Coll. Sanabria-Urbán S. & Cueva del Castillo R. | 19.324697 | -98.29475 | 25 | PU-TEC-10m | PP409881 |
| *Sphenarium purpurascens* | Mexico, Tlaxcala, Techachalco, L38, 11/9/2008, Coll. Sanabria-Urbán S. & Cueva del Castillo R. | 19.324697 | -98.29475 | 25 | PU-TEC-1m | PP409882 |
| *Sphenarium purpurascens* | Mexico, Tlaxcala, Techachalco, L38, 11/9/2008, Coll. Sanabria-Urbán S. & Cueva del Castillo R. | 19.324697 | -98.29475 | 25 | PU-TEC-2m | PP409883 |
| *Sphenarium purpurascens* | Mexico, Tlaxcala, Techachalco, L38, 11/9/2008, Coll. Sanabria-Urbán S. & Cueva del Castillo R. | 19.324697 | -98.29475 | 25 | PU-TEC-3f | PP409884 |
| *Sphenarium purpurascens* | Mexico, Tlaxcala, Techachalco, L38, 11/9/2008, Coll. Sanabria-Urbán S. & Cueva del Castillo R. | 19.324697 | -98.29475 | 25 | PU-TEC-4m | PP409885 |
| *Sphenarium purpurascens* | Mexico, Tlaxcala, Techachalco, L38, 11/9/2008, Coll. Sanabria-Urbán S. & Cueva del Castillo R. | 19.324697 | -98.29475 | 25 | PU-TEC-5f | PP409886 |
| *Sphenarium purpurascens* | Mexico, Tlaxcala, Techachalco, L38, 11/9/2008, Coll. Sanabria-Urbán S. & Cueva del Castillo R. | 19.324697 | -98.29475 | 25 | PU-TEC-6f | PP409887 |
| *Sphenarium purpurascens* | Mexico, Tlaxcala, Techachalco, L38, 11/9/2008, Coll. Sanabria-Urbán S. & Cueva del Castillo R. | 19.324697 | -98.29475 | 25 | PU-TEC-7f | PP409888 |
| *Sphenarium purpurascens* | Mexico, Tlaxcala, Techachalco, L38, 11/9/2008, Coll. Sanabria-Urbán S. & Cueva del Castillo R. | 19.324697 | -98.29475 | 25 | PU-TEC-8m | PP409889 |
| *Sphenarium purpurascens* | Mexico, Tlaxcala, Techachalco, L38, 11/9/2008, Coll. Sanabria-Urbán S. & Cueva del Castillo R. | 19.324697 | -98.29475 | 25 | PU-TEC-9m | PP409890 |
| *Sphenarium purpurascens* | Mexico, Tlaxcala, Zacualpan, L36, 11/9/2008, Coll. Sanabria-Urbán S. & Cueva del Castillo R. | 19.238442 | -98.258072 | 26 | PU-ZAC-11m | PP409891 |
| *Sphenarium purpurascens* | Mexico, Tlaxcala, Zacualpan, L36, 11/9/2008, Coll. Sanabria-Urbán S. & Cueva del Castillo R. | 19.238442 | -98.258072 | 26 | PU-ZAC-12m | PP409892 |
| *Sphenarium purpurascens* | Mexico, Tlaxcala, Zacualpan, L36, 11/9/2008, Coll. Sanabria-Urbán S. & Cueva del Castillo R. | 19.238442 | -98.258072 | 26 | PU-ZAC-13m | PP409893 |
| *Sphenarium purpurascens* | Mexico, Tlaxcala, Zacualpan, L36, 11/9/2008, Coll. Sanabria-Urbán S. & Cueva del Castillo R. | 19.238442 | -98.258072 | 26 | PU-ZAC-14m | PP409894 |
| *Sphenarium purpurascens* | Mexico, Tlaxcala, Zacualpan, L36, 11/9/2008, Coll. Sanabria-Urbán S. & Cueva del Castillo R. | 19.238442 | -98.258072 | 26 | PU-ZAC-15m | PP409895 |
| *Sphenarium purpurascens* | Mexico, Tlaxcala, Zacualpan, L36, 11/9/2008, Coll. Sanabria-Urbán S. & Cueva del Castillo R. | 19.238442 | -98.258072 | 26 | PU-ZAC-1m | PP409896 |
| *Sphenarium purpurascens* | Mexico, Tlaxcala, Zacualpan, L36, 11/9/2008, Coll. Sanabria-Urbán S. & Cueva del Castillo R. | 19.238442 | -98.258072 | 26 | PU-ZAC-2f | PP409897 |
| *Sphenarium purpurascens* | Mexico, Tlaxcala, Zacualpan, L36, 11/9/2008, Coll. Sanabria-Urbán S. & Cueva del Castillo R. | 19.238442 | -98.258072 | 26 | PU-ZAC-3m | PP409898 |
| *Sphenarium purpurascens* | Mexico, Tlaxcala, Zacualpan, L36, 11/9/2008, Coll. Sanabria-Urbán S. & Cueva del Castillo R. | 19.238442 | -98.258072 | 26 | PU-ZAC-4m | PP409899 |
| *Sphenarium purpurascens* | Mexico, Tlaxcala, Zacualpan, L36, 11/9/2008, Coll. Sanabria-Urbán S. & Cueva del Castillo R. | 19.238442 | -98.258072 | 26 | PU-ZAC-5m | PP409900 |
| *Sphenarium rugosum* | Mexico, Guerrero, Mirador Rio Mezcala Carr 95, L50, 24/10/2011, Coll. Sanabria-Urban S. & Díaz de la Vega A. | 17.898793 | -99.580017 | 41 | RU-11M-m1_ORSP57-L50 | PP409901 |
| *Sphenarium rugosum* | Mexico, Guerrero, Petaquillas, L52, 24/10/2011, Coll. Sanabria-Urban S. & Díaz de la Vega A. | 17.476755 | -99.459517 | 35 | RU-12C-m3_ORSP55-L52 | PP409902 |
| *Sphenarium rugosum* | Mexico, Guerrero, Huajuapan de Leon, L54, 28/10/2011, Coll. Sanabria-Urban S. & Díaz de la Vega A. | 17.827303 | -97.7889 | 36 | RU-36H-m2_ORSP56-L54 | PP409903 |
| *Sphenarium rugosum* | Mexico, Puebla, Tilapa, L82, 17/10/2011, Coll. Sanabria-Urbán S. & Jiménez-Arcos V.H. | 18.610559 | -98.546388 | 37 | RU-8TI-m22_ORSP101-L82 | PP409904 |
| *Sphenarium rugosum* | Mexico, Guerrero, Rumbo a Iguala Carr 95D km 44, L83, 24/10/2011, Coll. Sanabria-Urban S. & Díaz de la Vega A. | 18.41797 | -99.479383 | 38 | RU-9IG-m1_ORSP102-L83 | PP409905 |
| *Sphenarium rugosum* | Mexico, Michoacan, Entre Tuxpan y Ocurio Carr 15, L49, 6/12/2013, Coll. Sanabria-Urbán S., P. Fontana & Mariño-Pérez R. | 19.5125557 | -100.46963 | 39 | RU-L14-m1_ORSP52-L49 | PP409906 |
| *Sphenarium rugosum* | Mexico, Oaxaca, Freznillo Trujano, L84, 9/12/2013, Coll. Sanabria-Urbán S., P. Fontana & Mariño-Pérez R. | 17.9506259 | -98.132787 | 40 | RU-L22-m1_ORSP103-L84 | PP409907 |
| *Sphenarium rugosum* | Mexico, Mexico, Malinalco, L55, 12/9/2008, Coll. Sanabria-Urbán S. & Cueva del Castillo R. | 18.946547 | -99.487494 | 34 | RU-MAL-01m | PP409908 |
| *Sphenarium rugosum* | Mexico, Mexico, Malinalco, L55, 12/9/2008, Coll. Sanabria-Urbán S. & Cueva del Castillo R. | 18.946547 | -99.487494 | 34 | RU-MAL-02m | PP409909 |
| *Sphenarium rugosum* | Mexico, Mexico, Malinalco, L55, 12/9/2008, Coll. Sanabria-Urbán S. & Cueva del Castillo R. | 18.946547 | -99.487494 | 34 | RU-MAL-03f | PP409910 |
| *Sphenarium rugosum* | Mexico, Mexico, Malinalco, L55, 12/9/2008, Coll. Sanabria-Urbán S. & Cueva del Castillo R. | 18.946547 | -99.487494 | 34 | RU-MAL-04m | PP409911 |
| *Sphenarium rugosum* | Mexico, Mexico, Malinalco, L55, 12/9/2008, Coll. Sanabria-Urbán S. & Cueva del Castillo R. | 18.946547 | -99.487494 | 34 | RU-MAL-05f | PP409912 |
| *Sphenarium rugosum* | Mexico, Mexico, Malinalco, L55, 12/9/2008, Coll. Sanabria-Urbán S. & Cueva del Castillo R. | 18.946547 | -99.487494 | 34 | RU-MAL-10m | PP409913 |
| *Sphenarium rugosum* | Mexico, Mexico, Malinalco, L55, 12/9/2008, Coll. Sanabria-Urbán S. & Cueva del Castillo R. | 18.946547 | -99.487494 | 34 | RU-MAL-11m | PP409914 |
| *Sphenarium rugosum* | Mexico, Mexico, Malinalco, L55, 12/9/2008, Coll. Sanabria-Urbán S. & Cueva del Castillo R. | 18.946547 | -99.487494 | 34 | RU-MAL-12m | PP409915 |
| *Sphenarium rugosum* | Mexico, Mexico, Malinalco, L55, 12/9/2008, Coll. Sanabria-Urbán S. & Cueva del Castillo R. | 18.946547 | -99.487494 | 34 | RU-MAL-13m | PP409916 |
| *Sphenarium rugosum* | Mexico, Mexico, Malinalco, L55, 12/9/2008, Coll. Sanabria-Urbán S. & Cueva del Castillo R. | 18.946547 | -99.487494 | 34 | RU-MAL-14m | PP409917 |
| *Sphenarium rugosum* | Mexico, Guerrero, Ceibitas Carr 51, L87, 14/10/2012, Coll. Sanabria-Urbán S. & Pingarroni A. | 18.400313 | -100.083 | 42 | RU-P69-m1_ORSP109-L87 | PP409918 |
| *Sphenarium rugosum* | Mexico, Guerrero, Xixila, L56, 15/9/2011, Coll. Sanabria-Urbán S. & Cueva del Castillo R. | 17.952664 | -98.848483 | 43 | RU-XIX-01m | PP409919 |
| *Sphenarium rugosum* | Mexico, Guerrero, Xixila, L56, 15/9/2011, Coll. Sanabria-Urbán S. & Cueva del Castillo R. | 17.952664 | -98.848483 | 43 | RU-XIX-02m | PP409920 |
| *Sphenarium rugosum* | Mexico, Morelos, Yecapixtla, L53, 12/9/2008, Coll. Sanabria-Urbán S. & Cueva del Castillo R. | 18.89705 | -98.902911 | 33 | RU-YEC-01f | PP409921 |
| *Sphenarium rugosum* | Mexico, Morelos, Yecapixtla, L53, 12/9/2008, Coll. Sanabria-Urbán S. & Cueva del Castillo R. | 18.89705 | -98.902911 | 33 | RU-YEC-02m | PP409922 |
| *Sphenarium rugosum* | Mexico, Morelos, Yecapixtla, L53, 12/9/2008, Coll. Sanabria-Urbán S. & Cueva del Castillo R. | 18.89705 | -98.902911 | 33 | RU-YEC-03m | PP409923 |
| *Sphenarium rugosum* | Mexico, Morelos, Yecapixtla, L53, 12/9/2008, Coll. Sanabria-Urbán S. & Cueva del Castillo R. | 18.89705 | -98.902911 | 33 | RU-YEC-04f | PP409924 |
| *Sphenarium rugosum* | Mexico, Morelos, Yecapixtla, L53, 12/9/2008, Coll. Sanabria-Urbán S. & Cueva del Castillo R. | 18.89705 | -98.902911 | 33 | RU-YEC-05f | PP409925 |
| *Sphenarium rugosum* | Mexico, Morelos, Yecapixtla, L53, 12/9/2008, Coll. Sanabria-Urbán S. & Cueva del Castillo R. | 18.89705 | -98.902911 | 33 | RU-YEC-06f | PP409926 |
| *Sphenarium rugosum* | Mexico, Morelos, Yecapixtla, L53, 12/9/2008, Coll. Sanabria-Urbán S. & Cueva del Castillo R. | 18.89705 | -98.902911 | 33 | RU-YEC-07m | PP409927 |
| *Sphenarium rugosum* | Mexico, Morelos, Yecapixtla, L53, 12/9/2008, Coll. Sanabria-Urbán S. & Cueva del Castillo R. | 18.89705 | -98.902911 | 33 | RU-YEC-08m | PP409928 |
| *Sphenarium rugosum* | Mexico, Morelos, Yecapixtla, L53, 12/9/2008, Coll. Sanabria-Urbán S. & Cueva del Castillo R. | 18.89705 | -98.902911 | 33 | RU-YEC-09m | PP409929 |
| *Sphenarium rugosum* | Mexico, Morelos, Yecapixtla, L53, 12/9/2008, Coll. Sanabria-Urbán S. & Cueva del Castillo R. | 18.89705 | -98.902911 | 33 | RU-YEC-10f | PP409930 |
| *Sphenarium variabile* | Mexico, Oaxaca, Pasando San Juan Lajarcia Carr 190 km 150, L60, 27/10/2011, Coll. Sanabria-Urban S. & Díaz de la Vega A. | 16.556248 | -96.000787 | 27 | VA-27L-10f | PP409931 |
| *Sphenarium variabile* | Mexico, Oaxaca, Pasando San Juan Lajarcia Carr 190 km 150, L60, 27/10/2011, Coll. Sanabria-Urban S. & Díaz de la Vega A. | 16.556248 | -96.000787 | 27 | VA-27L-11f | PP409932 |
| *Sphenarium variabile* | Mexico, Oaxaca, Pasando San Juan Lajarcia Carr 190 km 150, L60, 27/10/2011, Coll. Sanabria-Urban S. & Díaz de la Vega A. | 16.556248 | -96.000787 | 27 | VA-27L-12f | PP409933 |
| *Sphenarium variabile* | Mexico, Oaxaca, Pasando San Juan Lajarcia Carr 190 km 150, L60, 27/10/2011, Coll. Sanabria-Urban S. & Díaz de la Vega A. | 16.556248 | -96.000787 | 27 | VA-27L-13f | PP409934 |
| *Sphenarium variabile* | Mexico, Oaxaca, Pasando San Juan Lajarcia Carr 190 km 150, L60, 27/10/2011, Coll. Sanabria-Urban S. & Díaz de la Vega A. | 16.556248 | -96.000787 | 27 | VA-27L-16f | PP409935 |
| *Sphenarium variabile* | Mexico, Oaxaca, Pasando San Juan Lajarcia Carr 190 km 150, L60, 27/10/2011, Coll. Sanabria-Urban S. & Díaz de la Vega A. | 16.556248 | -96.000787 | 27 | VA-27L-17f | PP409936 |
| *Sphenarium variabile* | Mexico, Oaxaca, Pasando San Juan Lajarcia Carr 190 km 150, L60, 27/10/2011, Coll. Sanabria-Urban S. & Díaz de la Vega A. | 16.556248 | -96.000787 | 27 | VA-27L-2m | PP409937 |
| *Sphenarium variabile* | Mexico, Oaxaca, Pasando San Juan Lajarcia Carr 190 km 150, L60, 27/10/2011, Coll. Sanabria-Urban S. & Díaz de la Vega A. | 16.556248 | -96.000787 | 27 | VA-27L-5m | PP409938 |
| *Sphenarium variabile* | Mexico, Oaxaca, Carr 190 km 65, L62, 27/10/2011, Coll. Sanabria-Urban S. & Díaz de la Vega A. | 16.762034 | -96.343341 | 28 | VA-31K-10m | PP409939 |
| *Sphenarium variabile* | Mexico, Oaxaca, Carr 190 km 65, L62, 27/10/2011, Coll. Sanabria-Urban S. & Díaz de la Vega A. | 16.762034 | -96.343341 | 28 | VA-31K-11m | PP409940 |
| *Sphenarium variabile* | Mexico, Oaxaca, Carr 190 km 65, L62, 27/10/2011, Coll. Sanabria-Urban S. & Díaz de la Vega A. | 16.762034 | -96.343341 | 28 | VA-31K-13m | PP409941 |
| *Sphenarium variabile* | Mexico, Oaxaca, Carr 190 km 65, L62, 27/10/2011, Coll. Sanabria-Urban S. & Díaz de la Vega A. | 16.762034 | -96.343341 | 28 | VA-31K-20f | PP409942 |
| *Sphenarium variabile* | Mexico, Oaxaca, Carr 190 km 65, L62, 27/10/2011, Coll. Sanabria-Urban S. & Díaz de la Vega A. | 16.762034 | -96.343341 | 28 | VA-31K-5m | PP409943 |
| *Sphenarium variabile* | Mexico, Oaxaca, Carr 190 km 65, L62, 27/10/2011, Coll. Sanabria-Urban S. & Díaz de la Vega A. | 16.762034 | -96.343341 | 28 | VA-31K-7m | PP409944 |
| *Sphenarium variabile* | Mexico, Oaxaca, Carr 190 km 65, L62, 27/10/2011, Coll. Sanabria-Urban S. & Díaz de la Vega A. | 16.762034 | -96.343341 | 28 | VA-31K-8m | PP409945 |
| *Sphenarium variabile* | Mexico, Oaxaca, Carr 175 Km 158, L63, 12/12/2013, Coll. Sanabria-Urbán S., P. Fontana & Mariño-Pérez R. | 16.0586885 | -96.498846 | 29 | VA-L32-2m | PP409946 |
| *Sphenarium variabile* | Mexico, Oaxaca, Carr 175 Km 158, L63, 12/12/2013, Coll. Sanabria-Urbán S., P. Fontana & Mariño-Pérez R. | 16.0586885 | -96.498846 | 29 | VA-L32-4f | PP409947 |
| *Sphenarium variabile* | Mexico, Oaxaca, Carr 175 Km 158, L63, 12/12/2013, Coll. Sanabria-Urbán S., P. Fontana & Mariño-Pérez R. | 16.0586885 | -96.498846 | 29 | VA-L32-6f | PP409948 |
| *Sphenarium variabile* | Mexico, Oaxaca, Carr 175 Km 87 , L61, 7/11/2018, Coll. Sanabria-Urbán S. & Cueva del Castillo R. | 16.3862211 | -96.656034 | 9 | VA-L36-1m | PP409949 |
| *Sphenarium variabile* | Mexico, Oaxaca, Carr 175 Km 87 , L61, 7/11/2018, Coll. Sanabria-Urbán S. & Cueva del Castillo R. | 16.3862211 | -96.656034 | 9 | VA-L36-27m | PP409950 |
| *Sphenarium variabile* | Mexico, Oaxaca, Carr 175 Km 87 , L61, 7/11/2018, Coll. Sanabria-Urbán S. & Cueva del Castillo R. | 16.3862211 | -96.656034 | 9 | VA-L36-3m | PP409951 |
| *Sphenarium variabile* | Mexico, Oaxaca, Carr 175 Km 87 , L61, 7/11/2018, Coll. Sanabria-Urbán S. & Cueva del Castillo R. | 16.3862211 | -96.656034 | 9 | VA-L36-4m | PP409952 |
| *Sphenarium variabile* | Mexico, Oaxaca, Carr 175 Km 87 , L61, 7/11/2018, Coll. Sanabria-Urbán S. & Cueva del Castillo R. | 16.3862211 | -96.656034 | 9 | VA-L36-5m | PP409953 |
| *Sphenarium variabile* | Mexico, Oaxaca, Carr 175 Km 87 , L61, 7/11/2018, Coll. Sanabria-Urbán S. & Cueva del Castillo R. | 16.3862211 | -96.656034 | 9 | VA-L36-6m | PP409954 |
| *Sphenarium variabile* | Mexico, Oaxaca, Carr 175 Km 87 , L61, 7/11/2018, Coll. Sanabria-Urbán S. & Cueva del Castillo R. | 16.3862211 | -96.656034 | 9 | VA-L36-7m | PP409955 |
| *Sphenarium variabile* | Mexico, Oaxaca, San Adres Paxtlan Carr 175 km 116, L39, 2/10/2012, Coll. Sanabria-Urban & Rivera-Ortiz F. | 16.241674 | -96.52472 | 17 | VA-P56-3m | PP409956 |
| *Sphenarium variabile* | Mexico, Oaxaca, San Adres Paxtlan Carr 175 km 116, L39, 2/10/2012, Coll. Sanabria-Urban & Rivera-Ortiz F. | 16.241674 | -96.52472 | 17 | VA-P56-9m | PP409957 |
| *Sphenarium zapotecum* | Mexico, Oaxaca, Plum a Hidalgo 1 , L91, 11/12/2013, Coll. Sanabria-Urbán S., P. Fontana & Mariño-Pérez R. | 15.9398788 | -96.429961 | 30 | ZA-L27-1m | PP409958 |
| *Sphenarium zapotecum* | Mexico, Oaxaca, Plum a Hidalgo 1 , L91, 11/12/2013, Coll. Sanabria-Urbán S., P. Fontana & Mariño-Pérez R. | 15.9398788 | -96.429961 | 30 | ZA-L27-2m | PP409959 |
| *Sphenarium zapotecum* | Mexico, Oaxaca, Plum a Hidalgo 1 , L91, 11/12/2013, Coll. Sanabria-Urbán S., P. Fontana & Mariño-Pérez R. | 15.9398788 | -96.429961 | 30 | ZA-L27-3m | PP409960 |
| *Sphenarium zapotecum* | Mexico, Oaxaca, Plum a Hidalgo 1 , L91, 11/12/2013, Coll. Sanabria-Urbán S., P. Fontana & Mariño-Pérez R. | 15.9398788 | -96.429961 | 30 | ZA-L27-4f | PP409961 |
| *Sphenarium zapotecum* | Mexico, Oaxaca, Plum a Hidalgo 1 , L91, 11/12/2013, Coll. Sanabria-Urbán S., P. Fontana & Mariño-Pérez R. | 15.9398788 | -96.429961 | 30 | ZA-L27-5f | PP409962 |
| *Sphenarium zapotecum* | Mexico, Oaxaca, Pluma Hidalgo 2 , L92, 11/12/2013, Coll. Sanabria-Urbán S., P. Fontana & Mariño-Pérez R. | 15.9566251 | -96.45034 | 31 | ZA-L28-4m | PP409963 |
| *Sphenarium zapotecum* | Mexico, Oaxaca, Pluma Hidalgo 2 , L92, 11/12/2013, Coll. Sanabria-Urbán S., P. Fontana & Mariño-Pérez R. | 15.9566251 | -96.45034 | 31 | ZA-L28-5m | PP409964 |
| *Sphenarium zapotecum* | Mexico, Oaxaca, Pluma Hidalgo 2 , L92, 11/12/2013, Coll. Sanabria-Urbán S., P. Fontana & Mariño-Pérez R. | 15.9566251 | -96.45034 | 31 | ZA-L28-6m | PP409965 |
| *Sphenarium zapotecum* | Mexico, Oaxaca, Pluma Hidalgo 2 , L92, 11/12/2013, Coll. Sanabria-Urbán S., P. Fontana & Mariño-Pérez R. | 15.9566251 | -96.45034 | 31 | ZA-L28-7m | PP409966 |
| *Sphenarium zapotecum* | Mexico, Oaxaca, Pluma Hidalgo 2 , L92, 11/12/2013, Coll. Sanabria-Urbán S., P. Fontana & Mariño-Pérez R. | 15.9566251 | -96.45034 | 31 | ZA-L28-9f | PP409967 |
| *Sphenarium zapotecum* | Mexico, Oaxaca, Carr 175 Km 172 , L93, 12/12/2013, Coll. Sanabria-Urbán S., P. Fontana & Mariño-Pérez R. | 16.0186485 | -96.530311 | 32 | ZA-L31-3m | PP409968 |
| *Sphenarium zapotecum* | Mexico, Oaxaca, Carr 175 Km 172 , L93, 12/12/2013, Coll. Sanabria-Urbán S., P. Fontana & Mariño-Pérez R. | 16.0186485 | -96.530311 | 32 | ZA-L31-4f | PP409969 |
| *Sphenarium zapotecum* | Mexico, Oaxaca, Carr 175 Km 172 , L93, 12/12/2013, Coll. Sanabria-Urbán S., P. Fontana & Mariño-Pérez R. | 16.0186485 | -96.530311 | 32 | ZA-L31-5f | PP409970 |
| *Sphenarium crypticum** |  |  |  |  | ORSP53 | KU146983 |
| *Sphenarium macrophallicum** |  |  |  |  | ORSP05 | KU146937 |
| *Sphenarium minimum** |  |  |  |  | ORSP26 | KU146957 |
| *Sphenarium planum** |  |  |  |  | ORSP49 | KU146980 |
| *Sphenarium tarascum** |  |  |  |  | ORSP46 | KU146977 |

*, Sequences generated previously by Sanabria-Urbán, S., Song, H., Oyama, K., González-Rodríguez, A. & Castillo, R. C. D. (2017). Integrative taxonomy reveals cryptic diversity in neotropical grasshoppers: taxonomy, phylogenetics, and evolution of the genus Sphenarium Charpentier, 1842 (Orthoptera: Pyrgomorphidae). *Zootaxa*, *4274*(1), 1–86. https://doi.org/10.11646/zootaxa.4274.1.1

**Table S2**. Nucleotide substitution models and number of segments applied for each dataset for the Bayesian Skyline Plot analyses in the software BEAST.

| Dataset | Substitution model | Number of segments |
| --- | --- | --- |
| S. purpurascens (pooled populations) | HKY | 10 |
| S. purpurascens, population P1 | HKY+I | 10 |
| S. purpurascens, population P2 | HKY+I | 10 |
| S. purpurascens, population P3 | HKY | 10 |
| S. purpurascens, population P4 | HKY+I | 10 |
| S. purpurascens, population P5 | HKY | 10 |
| S. purpurascens, population P6 | HKY | 10 |
| S. purpurascens, population P9 | HKY | 5 |
| S. rugosum (pooled populations) | HKY | 10 |
| S. rugosum, population R1 | JC | 5 |
| S. rugosum, population R2 | HKY | 5 |
| S. variabile (pooled populations) | HKY+I | 10 |
| S. variabile, population V1 | JC | 5 |
| S. variabile, population V2 | JC | 5 |
| S. variabile, population V3 | HKY | 10 |
| S. zapotecum (pooled populations) | HKY | 10 |

**Table S3**. Georeferenced records of *S. purpurascens*, *S. rugosum*, *S. variabile* and *S. zapotecum* used for the ecological niche models.

| **Species** | **Locality** | **Latitude** | **Longitude** |
| --- | --- | --- | --- |
| *Sphenarium purpurascens* | Rumbo a Teotitlan Carr 131 Km 71 | 18.08045 | -97.06484 |
| *Sphenarium purpurascens* | Papalotla | 19.16221 | -98.2235 |
| *Sphenarium purpurascens* | Cacaxtla | 19.11656 | -98.38875 |
| *Sphenarium purpurascens* | Cholula | 19.05636 | -98.30425 |
| *Sphenarium purpurascens* | Monte Alban | 17.04705 | -96.76428 |
| *Sphenarium purpurascens* | Rumbo a Peña de Bernal | 20.56416 | -100.21541 |
| *Sphenarium purpurascens* | Acambaro | 20.15817 | -100.54929 |
| *Sphenarium purpurascens* | Desviasión a Amealco | 20.11361 | -99.70306 |
| *Sphenarium purpurascens* | Rumbo a Guanajuato | 19.93804 | -100.76991 |
| *Sphenarium purpurascens* | Atotonilco | 19.37351 | -98.4653 |
| *Sphenarium purpurascens* | Techachalco | 19.3247 | -98.29475 |
| *Sphenarium purpurascens* | Zacualpan | 19.23844 | -98.25807 |
| *Sphenarium purpurascens* | Rumbo a Morelia Carr14 Km 19 | 19.56944 | -101.32089 |
| *Sphenarium purpurascens* | Pasando Sta Clara del Cobre Carr120 | 19.42436 | -101.60483 |
| *Sphenarium purpurascens* | Desviasión a Araro Carr 15 | 19.90094 | -100.8265 |
| *Sphenarium purpurascens* | Tlacolula Carr 190 | 16.90298 | -96.39572 |
| *Sphenarium purpurascens* | Yautitlan Carr 190 | 17.5354 | -97.35231 |
| *Sphenarium purpurascens* | Tamazulapan Carr 190 | 17.67751 | -97.57972 |
| *Sphenarium purpurascens* | Entre Huajuapan y Tamazulapan Carr 190 | 17.72168 | -97.66633 |
| *Sphenarium purpurascens* | Tultepec | 19.69151 | -99.13264 |
| *Sphenarium purpurascens* | Cumbres de Maltrata Carr 150D | 18.82787 | -97.25417 |
| *Sphenarium purpurascens* | San Adres Paxtlan Carr 175 km 116 | 16.24167 | -96.52472 |
| *Sphenarium purpurascens* | Miahuatlan Carr 175 | 16.31231 | -96.58809 |
| *Sphenarium purpurascens* | Ejutla Carr 175 | 16.52271 | -96.73626 |
| *Sphenarium purpurascens* | Rumbo a Hierve el Agua | 16.93418 | -96.3173 |
| *Sphenarium purpurascens* | Penuelas Carr. 15 Km 5 | 19.70861 | -100.74895 |
| *Sphenarium purpurascens* | Rumbo a Zitacuaro | 19.51256 | -100.46963 |
| *Sphenarium purpurascens* | Camino a Huiloapan | 19.4275 | -98.29794 |
| *Sphenarium purpurascens* | Ixtenco pop Victor | 19.22769 | -97.91208 |
| *Sphenarium purpurascens* | Frontera Oaxaca Puebla Carr 190 | 17.9117 | -97.85666 |
| *Sphenarium purpurascens* | Carr 175 Km 87 | 16.38622 | -96.65603 |
| *Sphenarium purpurascens* | El Capulin | 17.51683 | -96.94341 |
| *Sphenarium purpurascens* | Rumbo a Tonaltepec | 17.56731 | -96.94911 |
| *Sphenarium purpurascens* | Desviacion Dolores | 20.89858 | -101.06049 |
| *Sphenarium purpurascens* | Leon | 21.04154 | -101.56044 |
| *Sphenarium purpurascens* | Sta Catarina | 21.13617 | -100.06848 |
| *Sphenarium purpurascens* | Carr 85 entre Tasquillo y Aguacatal | 20.60108 | -99.33931 |
| *Sphenarium purpurascens* | Desviacion Quiroga | 19.60216 | -101.4286 |
| *Sphenarium purpurascens* | Carr 15 Km15, cerca de Zacapú | 19.7324 | -101.64865 |
| *Sphenarium purpurascens* | Los Alferes Panjamo Gto. | 20.43962 | -101.73261 |
| *Sphenarium purpurascens* | San Juan del Río Qto. | 20.36738 | -100.02054 |
| *Sphenarium purpurascens* | San Antonio, Tarandacuao Gto. | 19.97679 | -100.52759 |
| *Sphenarium purpurascens* | El Copal Irapuato Gto. | 20.65149 | -101.34701 |
| *Sphenarium purpurascens* | Valenciacnita Gto. | 20.74291 | -101.30147 |
| *Sphenarium purpurascens* | Abasolo Gto. | 20.45187 | -101.53018 |
| *Sphenarium purpurascens* | Tarandacuao Gto. | 19.99792 | -100.53351 |
| *Sphenarium purpurascens* | Gervacio Mendoza Valle De Santiago | 20.38723 | -101.19896 |
| *Sphenarium purpurascens* | Pedro Escobedo Qto. | 20.49684 | -100.14997 |
| *Sphenarium purpurascens* | Ezequiel Montes Qto. | 20.66319 | -99.89807 |
| *Sphenarium purpurascens* | Cedereyta Qto. | 20.69275 | -99.81781 |
| *Sphenarium purpurascens* | Huimilpan Qto. | 20.3675 | -100.27743 |
| *Sphenarium purpurascens* | Tequisquiapan Qto. | 20.5138 | -99.90103 |
| *Sphenarium purpurascens* | Amealco Qto. | 20.18196 | -100.1469 |
| *Sphenarium purpurascens* | Corregidora Qto. | 20.48144 | -100.44011 |
| *Sphenarium purpurascens* | La Tinaja Corregidora Qto. | 20.47779 | -100.41625 |
| *Sphenarium purpurascens* | Carr. 175 (ib09) | 16.38597 | -96.65519 |
| *Sphenarium purpurascens* | Carr 125 km43.5 ib09 | 17.365 | -97.61028 |
| *Sphenarium purpurascens* | Carr 190 km 44.3 Tamazulapan ib09 | 17.66861 | -97.52636 |
| *Sphenarium purpurascens* | Carr. Tehuacan-Oaxaca ib09 | 17.48944 | -96.93819 |
| *Sphenarium purpurascens* | spp el chapulin ib09 | 17.53492 | -96.95386 |
| *Sphenarium purpurascens* | spp tonaltepec ib09 | 17.57278 | -96.94603 |
| *Sphenarium purpurascens* | spp rio poblano ib08 | 17.79158 | -97.25678 |
| *Sphenarium purpurascens* | spp yoguna ib08 | 16.48333 | -96.73761 |
| *Sphenarium purpurascens* | spp nacaltepec ib08 | 17.50404 | -96.92317 |
| *Sphenarium purpurascens* | spp coixtlahuaca ib08 | 17.72972 | -97.30969 |
| *Sphenarium purpurascens* | spp san juan nacaltepec ib09 | 17.56703 | -96.93544 |
| *Sphenarium purpurascens* | spp san juan los cues ib09 | 18.03472 | -97.06444 |
| *Sphenarium purpurascens* | spp rio blanco ib08 | 17.73539 | -97.25244 |
| *Sphenarium purpurascens* | spp. santa ma. coatlan ib08 | 16.28681 | -96.70461 |
| *Sphenarium purpurascens* | spp presa yosocuta ib09 | 17.72564 | -97.83159 |
| *Sphenarium purpurascens* | spp ejutla/miahuatlan ib08 | 16.4605 | -96.72139 |
| *Sphenarium purpurascens* | spp. san vicente coatlan ib08 | 16.40178 | -96.80072 |
| *Sphenarium purpurascens* | spp. tlacolula de matamoros ib08 | 16.95444 | -96.47614 |
| *Sphenarium purpurascens* | Car. 131 Oax.-Pto. Escondido La cumbres | 16.46431 | -96.99419 |
| *Sphenarium purpurascens* | Car. 175 Oax.-Pto. angel Km 53 | 16.61881 | -96.7385 |
| *Sphenarium purpurascens* | San Geronimo, Pedregal | 19.32104 | -99.18441 |
| *Sphenarium purpurascens* | Sacatepec, Pedregal | 19.35241 | -99.0134 |
| *Sphenarium purpurascens* | Lomas Altas | 19.39693 | -99.22947 |
| *Sphenarium purpurascens* | Tlalpan | 19.30326 | -99.1984 |
| *Sphenarium purpurascens* | 15mi W Ixmiquilpan | 20.4328 | -99.4394 |
| *Sphenarium purpurascens* | Nochixtlan | 17.45869 | -97.22349 |
| *Sphenarium purpurascens* | Miahuatlan | 16.32889 | -96.59348 |
| *Sphenarium purpurascens* | Ejutla | 16.56541 | -96.73127 |
| *Sphenarium purpurascens* | Huajuapan | 17.80451 | -97.76839 |
| *Sphenarium purpurascens* | 1.0mi NW Tamazulapan at Km 388 (Km38.8) | 17.68082 | -97.58253 |
| *Sphenarium purpurascens* | 15.5mi NW Oaxaca | 17.25923 | -96.89975 |
| *Sphenarium purpurascens* | 6mi NW Oaxaca, main Plaza, on Hwy 190 | 17.13141 | -96.77202 |
| *Sphenarium purpurascens* | Tlaxiaco | 17.26916 | -97.67911 |
| *Sphenarium purpurascens* | 10mi SE Nochixtlan | 17.37724 | -97.11119 |
| *Sphenarium purpurascens* | 31mi SE Nochixtlan on Hwy 190 | 17.22559 | -96.87459 |
| *Sphenarium purpurascens* | Yanhuitlan | 17.52621 | -97.34392 |
| *Sphenarium purpurascens* | 26mi NE Sola de Vega on Mex 131, rd E to Santiago Textitlan | 16.70303 | -96.85304 |
| *Sphenarium purpurascens* | San Andres Chicohuaxtla | 17.15508 | -97.83904 |
| *Sphenarium purpurascens* | 12mi SW Sola de Vega on Mex 131 | 16.47519 | -97.01764 |
| *Sphenarium purpurascens* | 12 mi SE Nochixtlan | 17.35733 | -97.07255 |
| *Sphenarium purpurascens* | 13mi S Ocotlan, rd to Pto Angel #39 | 16.60126 | -96.73628 |
| *Sphenarium purpurascens* | 5mi N Tizayuca | 19.89347 | -98.93295 |
| *Sphenarium purpurascens* | 7mi S Oaxaca rd to Pto. Angel #38 | 16.95382 | -96.70606 |
| *Sphenarium purpurascens* | Agua Fria Grnade | 20.98216 | -99.21122 |
| *Sphenarium purpurascens* | Atizapan | 19.55259 | -99.26771 |
| *Sphenarium purpurascens* | Cerro San Felipe | 17.09487 | -96.74565 |
| *Sphenarium purpurascens* | Ejutla | 16.56053 | -96.73237 |
| *Sphenarium purpurascens* | Ixmiquilpan | 20.47848 | -99.21833 |
| *Sphenarium purpurascens* | Lomas de Chapultepec | 19.42014 | -99.21891 |
| *Sphenarium purpurascens* | Mitla | 16.91022 | -96.36268 |
| *Sphenarium purpurascens* | Monte Alban | 17.03873 | -96.76767 |
| *Sphenarium purpurascens* | Oaxaca-Pto Angel rd, 49rd mi S Ejutla # 41 | 16.07872 | -96.46811 |
| *Sphenarium purpurascens* | Oaxaca-Pto Angel rd, ca 10 mi S Ejutla # 40 | 16.43701 | -96.7012 |
| *Sphenarium purpurascens* | Tlalpan | 19.29284 | -99.19718 |
| *Sphenarium purpurascens* | Mexico City, Res. Pedregal de San angel Facultad de Ciencias | 19.32397 | -99.18136 |
| *Sphenarium purpurascens* | Ixmiquilpan Panales, cross Queretaro - Zimapan | 20.48403 | -99.29203 |
| *Sphenarium purpurascens* | Ixmiquilpan Panales, 4 km after cross to Zimapan | 20.51064 | -99.31061 |
| *Sphenarium purpurascens* | Queretaro N rd. to La Cañada &Tequisquiapan | 20.58475 | -100.35442 |
| *Sphenarium purpurascens* | Ixmiquilpan Panales, Motel El Paraiso | 20.48334 | -99.28881 |
| *Sphenarium purpurascens* | Rd. to Queretaro, km 24, 6 Km E Huichapan | 20.42683 | -99.48419 |
| *Sphenarium purpurascens* | Acambaro, 2km NE. rd. 120 to Jerecuaro | 20.05131 | -100.69769 |
| *Sphenarium purpurascens* | Queretaro SE between La Cañada and El Milagro | 20.61953 | -100.31386 |
| *Sphenarium purpurascens* | Tula, 4 km w of Michimaloya, road to S. Miguel de las Piedras | 20.09917 | -99.43661 |
| *Sphenarium purpurascens* | Atenco | 19.54389 | -98.95861 |
| *Sphenarium purpurascens* | Tula, Presa W side, loc. San Pedro | 20.15881 | -99.37597 |
| *Sphenarium purpurascens* | Santa Maria Jalteanguis | 17.35841897 | -96.521769 |
| *Sphenarium purpurascens* | San Juan Atepec | 17.43977398 | -96.514817 |
| *Sphenarium purpurascens* | Ixtlán | 17.32114197 | -96.484014 |
| *Sphenarium rugosum* | Malinalco | 18.94655 | -99.48749 |
| *Sphenarium rugosum* | Yecapixtla | 18.89705 | -98.90291 |
| *Sphenarium rugosum* | Rumbo a Iguala Carr 95D km 44 | 18.41797 | -99.47938 |
| *Sphenarium rugosum* | Pasando Iguala Carr 95 | 18.30151 | -99.50745 |
| *Sphenarium rugosum* | Pasando Rio Mezcala Carr 95 km 175 | 17.89879 | -99.58002 |
| *Sphenarium rugosum* | Huajuapan de Leon Carr 190 | 17.8273 | -97.7889 |
| *Sphenarium rugosum* | Petaquillas Carr 95 km 14 | 17.47676 | -99.45952 |
| *Sphenarium rugosum* | Palo Blanco Carr 95 | 17.40981 | -99.46677 |
| *Sphenarium rugosum* | Desviasion a Atenango del Rio Carr 1 | 18.22908 | -99.21515 |
| *Sphenarium rugosum* | Copalillo desviacion a Papalutla Carr 7 | 18.06543 | -99.03882 |
| *Sphenarium rugosum* | Barranca Papalutla | 18.01865 | -98.88996 |
| *Sphenarium rugosum* | Rumbo a Xixila | 17.99369 | -98.8512 |
| *Sphenarium rugosum* | Xixila | 17.95266 | -98.84848 |
| *Sphenarium rugosum* | Axochiapan | 18.47705 | -98.7336 |
| *Sphenarium rugosum* | Tilapa | 18.61056 | -98.54639 |
| *Sphenarium rugosum* | Alpuyeca Carr 95D | 18.71842 | -99.26575 |
| *Sphenarium rugosum* | Rumbo a Huajuapan | 18.05076 | -97.69076 |
| *Sphenarium rugosum* | Frontera Oaxaca Puebla | 17.9117 | -97.85666 |
| *Sphenarium rugosum* | Freznillo Trujano | 17.95063 | -98.13279 |
| *Sphenarium rugosum* | Atixtac Km 52 Tlapa-Chilapa Gro. | 17.54917 | -98.91556 |
| *Sphenarium rugosum* | Tlayacapan, Sn. Jose de los Laureles Mor. | 18.98197 | -99.00019 |
| *Sphenarium rugosum* | Tepalcingo Mor. | 18.59583 | -98.84401 |
| *Sphenarium rugosum* | Cuautla Mor. | 18.80408 | -98.95246 |
| *Sphenarium rugosum* | Tixtla Km 29 Chilpancingo-Chilapa | 17.60306 | -99.32278 |
| *Sphenarium rugosum* | Chilpancingo Der. El alquitran Gro. | 17.4375 | -99.49222 |
| *Sphenarium rugosum* | 11 km SE Izucar de Matamoros, rd. 190 | 18.53742 | -98.43086 |
| *Sphenarium rugosum* | 20 km NW. 10 km SE Acatl├ƒn rd. 190 | 18.14078 | -98.01153 |
| *Sphenarium rugosum* | S of Cuernavaca, Mazatepec | 18.72431 | -99.37025 |
| *Sphenarium rugosum* | S of Cuernavaca, Amacuzac | 18.61931 | -99.36886 |
| *Sphenarium rugosum* | Cuernavaca | 18.93402 | -99.23148 |
| *Sphenarium rugosum* | 4mi N Chilpancingo | 17.64465 | -99.50488 |
| *Sphenarium rugosum* | 3mi N Chilpancingo | 17.63046 | -99.50939 |
| *Sphenarium rugosum* | Tepoztlan | 18.985 | -99.1 |
| *Sphenarium rugosum* | 14mi S Chilpancingo #52 | 17.37851 | -99.47708 |
| *Sphenarium rugosum* | 1mi E Tepoztlan | 18.97876 | -99.08123 |
| *Sphenarium rugosum* | 3mi SE Petlalcingo | 18.05128 | -97.90353 |
| *Sphenarium rugosum* | 1.2mi W Tehuitzingo on Hwy 190 | 18.3389 | -98.28353 |
| *Sphenarium rugosum* | 9mi S Cuernavaca on Hwy 95 | 18.83814 | -99.22498 |
| *Sphenarium rugosum* | 16rd mi ENE Taxco, 4.7rd mi NE Acuitlapan | 18.60703 | -99.49406 |
| *Sphenarium rugosum* | 9rd mi NE Taxco, 1.7rd mi SW Acuitlapan | 18.59567 | -99.55396 |
| *Sphenarium rugosum* | 1mi S Rio Balsas on Hwy 95, 33mi N Chilpancingo | 17.92304 | -99.5895 |
| *Sphenarium rugosum* | 3rd mi SW Almolonga, 8mi NE Tixtla | 17.60042 | -99.31652 |
| *Sphenarium rugosum* | 10mi S Chilpancingo on Hwy 95, 1.5mi N Mazatlan turnoff | 17.44876 | -99.46916 |
| *Sphenarium rugosum* | 6mi S Iguala on Hwy 95 | 18.27945 | -99.51506 |
| *Sphenarium rugosum* | 9rd mi NE Taxco, 2rd mi SW Acuitlapan | 18.59101 | -99.55572 |
| *Sphenarium rugosum* | 6mi NW Petlalcingo on Hwy 190 | 18.1426 | -97.98573 |
| *Sphenarium rugosum* | 2.4mi SW Almolonga, 9.4mi NE Tixtla | 17.6073 | -99.3159 |
| *Sphenarium rugosum* | 4.3mi E Petaquillas, 1.3mi E Tepechicotlan | 17.47411 | -99.39411 |
| *Sphenarium rugosum* | 8.5mi SE Iguala on Huitzuco rd | 18.26311 | -99.4426 |
| *Sphenarium rugosum* | 19mi W Iguala on Arcelia rd | 18.35057 | -99.84691 |
| *Sphenarium rugosum* | 18rd mi NE Taxco, 7rd mi NE Acuitlapan | 18.68905 | -99.48591 |
| *Sphenarium rugosum* | 16rd mi NE Taxco, 5rd mi NE Acuitlapan | 18.66823 | -99.49889 |
| *Sphenarium rugosum* | 1mi S Taxco | 18.52044 | -99.60497 |
| *Sphenarium rugosum* | 12rd mi NW of Iguala | 18.38431 | -99.68143 |
| *Sphenarium rugosum* | 6mi W Iguala | 18.31476 | -99.61479 |
| *Sphenarium rugosum* | 2.2rd mi S Petaquillas | 17.46757 | -99.45555 |
| *Sphenarium rugosum* | 4mi SE Chilpancingo | 17.51847 | -99.44738 |
| *Sphenarium rugosum* | 6mi S Petaquillas | 17.40933 | -99.46321 |
| *Sphenarium rugosum* | Ixtapa de la Sal | 18.84322 | -99.67226 |
| *Sphenarium rugosum* | Matamoros | 18.60168 | -98.46617 |
| *Sphenarium rugosum* | Ceibitas Carr 51 | 18.40031 | -100.083 |
| *Sphenarium rugosum* | Tejupilco Carr 134 | 18.93259 | -100.21336 |
| *Sphenarium rugosum* | Rumbo a Zitacuaro | 19.51256 | -100.46963 |
| *Sphenarium variabile* | Carr 190 km 65 | 16.76203 | -96.34334 |
| *Sphenarium variabile* | Pasando San Juan Lajarcia Carr 190 km 150 | 16.53892 | -95.93637 |
| *Sphenarium variabile* | Carr 175 Km 87 | 16.38622 | -96.65603 |
| *Sphenarium variabile* | 2mi SE Tlacolula on Hwy #190 | 16.94104 | -96.44495 |
| *Sphenarium variabile* | 45mi NW Tequisistlan (1mi S El Camaron) | 16.6679 | -96.11797 |
| *Sphenarium variabile* | 18mi Nw La Reforma (Km695 onHwy 190; 31mi WNW Tequisistlan) | 16.54512 | -95.97994 |
| *Sphenarium variabile* | 13mi SE Tlacolula | 16.82096 | -96.35734 |
| *Sphenarium variabile* | 7mi SE El Camaron (37mi NW Tequisistlan onhwy 190) | 16.52268 | -95.92578 |
| *Sphenarium variabile* | Mitla | 16.92128 | -96.36165 |
| *Sphenarium variabile* | Carr 175 Km 172 | 16.01865 | -96.53031 |
| *Sphenarium variabile* | Carr 175 Km 158 | 16.05869 | -96.49885 |
| *Sphenarium variabile* | San Adres Paxtlan Carr 175 km 116 | 16.24167 | -96.52472 |
| *Sphenarium variabile* | Camino a San Pablo Coatlan 2 | 16.22873104 | -96.709925 |
| *Sphenarium variabile* | 46mi W Jalapa del Marquez at Portillo Nejapam pass | 16.547761 | -95.948928 |
| *Sphenarium variabile* | Portillo San Dionisio | 16.77638699 | -96.353185 |
| *Sphenarium variabile* | Entre km 55 y km54, Carr. 190 | 16.82244002 | -96.36101 |
| *Sphenarium zapotecum* | Copalita Carr 175 | 15.96039 | -96.46172 |
| *Sphenarium zapotecum* | Plum a Hidalgo 1 | 15.93988 | -96.42996 |
| *Sphenarium zapotecum* | Pluma Hidalgo 2 | 15.95663 | -96.45034 |
| *Sphenarium zapotecum* | Carr 175 Km184 | 15.98294 | -96.52032 |
| *Sphenarium zapotecum* | Carr 175 Km 172 | 16.01865 | -96.53031 |
| *Sphenarium zapotecum* | spp pluma hidalgo/huatulco ib08 | 15.88194 | -96.39556 |
| *Sphenarium zapotecum* | spp cerca pluma hidalgo ib09 | 15.92639 | -96.42333 |
| *Sphenarium zapotecum* | Car. 131 Oax.-Pto. Escondido Rio Salado | 16.19525 | -97.11925 |
| *Sphenarium zapotecum* | 24-25mi N Pto Escondido Rd to Oaxaca #45 | 16.16656 | -97.09174 |
| *Sphenarium zapotecum* | Oaxaca road ca. 85km N Pto Angel #43 | 16.07159 | -96.4898 |

**Table S4**. SAMOVA results for each number of genetic groups (K) tested for *S. purpurascens* and *S. variabile*. Bold numbers indicate the *K* value used for configuration of geographic groups, in which differentiation between the groups (*𝛷_CT_*) was the highest and stopped increasing.

|  | *K* values tested for *S. purpurascens* | | | | | | | | | | | | | |  | *K* values tested for *S. variabile* | | |
| --- | --- | --- | --- | --- | --- | --- | --- | --- | --- | --- | --- | --- | --- | --- | --- | --- | --- | --- |
| Index | 2 | 3 | 4 | 5 | 6 | 7 | 8 | **9** | 10 | 11 | 12 | 13 | 14 | 15 |  | 2 | **3** | 4 |
| *𝛷_CT_* | 0.60 | 0.58 | 0.62 | 0.65 | 0.66 | 0.65 | 0.64 | **0.70** | 0.67 | 0.69 | 0.70 | 0.69 | 0.72 | 0.70 |  | 0.46 | **0.80** | 0.78 |
| *𝛷_SC_* | 0.50 | 0.50 | 0.43 | 0.38 | 0.35 | 0.27 | 0.26 | **0.14** | 0.19 | 0.13 | 0.11 | 0.12 | 0.03 | 0.07 |  | 0.70 | **-0.03** | 0.16 |
| *𝛷_ST_* | 0.80 | 0.79 | 0.79 | 0.78 | 0.78 | 0.74 | 0.74 | **0.74** | 0.73 | 0.73 | 0.73 | 0.73 | 0.73 | 0.73 |  | 0.84 | **0.80** | 0.82 |

**Table S5**. Summarized parameter settings and statistical performance values obtained for individual models of the four *Sphenarium*. Acronyms into feature response type correspond to: linear [L], quadratic [Q], product [P], threshold [T], and Hinge [H]

| **Species** | **Occurrence points** | **Regularizator multiple** | **Feature response type** | **Mean value for AUC** | **AICc** | **ROC-Partial value** | **Threshold value** | **%Omission testing data** |
| --- | --- | --- | --- | --- | --- | --- | --- | --- |
| *S. purpurascens* | 102 | 0.4 | LQ | 0.756 | 1,514.53 | 1.19 | 0.228 | 7.14% |
| *S. variabile* | 12 | 2 | LQ | 0.831 | 186.69 | 1.99 | 0.564 | 8.33% |
| *S. rugosum* | 51 | 1.2 | LQP | 0.818 | 746.52 | 1.38 | 0.384 | 12.5% |
| *S. zapotecum* | 8 | 1 | LQ | 0.782 | 101.02 | 1.98 | 0.502 | 12.5% |

**Table S6**. Summarize the individual results for pair-comparison between the four *Sphenarium* species considering the observed values of niche overlap (based on Schoener’s D index) and the simulated overlap from the 1,000 pseudo-replicated data sets. For each comparison, we reported the number of occurrence points used per species, the explained variation for the two principal components of PCA-env, their values of niche overlap, as well as the values for the Niche Equivalency and background Similarity tests.

| **Species pair comparison** | **Explained variation PC1/PC2** (Total) | **Empirical Shoener’s *D* value** | **Niche Equivalency test** (*P-*values) | **Niche Similarity test** (*P-*values)  **A-B / B-A** |
| --- | --- | --- | --- | --- |
| only climatic data | | | | |
| *S. purpurascens* vs. *S. rugosum* | 42.66 / 32.95 (75.61%) | 0.129 | 0.965 | 0.348 / 0.301 |
| *S. purpurascens* vs. *S. variabile* | 40.16 / 35.12 (75.28 %) | 0.227 | 1.000 | 0.192 / 0.202 |
| *S. purpurascens* vs. *S. zapotecum* | 40.75 / 34.08 (74.83 %) | 0.001 | 1.000 | 1.000 / 1.000 |
| *S. rugosum* vs. *S. variabile* | 46.32 / 30.81 (77.13 %) | 0.281 | 0.934 | 0.073 / 0.119 |
| *S. rugosum* vs. *S. zapotecum* | 48.65 / 27.21 (75.86 %) | 0.008 | 1.000 | 1.000 / 1.000 |
| *S. variabile* vs. *S. zapotecum* | 45.04 / 30.99 (76.03 %) | 0.023 | 1.000 | 1.000 / 1.000 |
| climate-plus-habitat | | | | |
| *S. purpurascens* vs. *S. rugosum* | 37.73 / 28.43 (66.16%) | 0.092 | 0.949 | 0.435 / 0.320 |
| *S. purpurascens* vs. *S. variabile* | 36.12 / 29.75 (65.87%) | 0.322 | 0.922 | 0.092 / 0.010* |
| *S. purpurascens* vs. *S. zapotecum* | 36.38 / 29.08 (65.46%) | 0.000 | 1.000 | 1.000 / 1.000 |
| *S. rugosum* vs. *S. variabile* | 40.73 / 26.24 (66.93%) | 0.415 | 0.655 | 0.030* / 0.040* |
| *S. rugosum* vs. *S. zapotecum* | 42.63 / 23.22 (65.85%) | 0.002 | 1.000 | 0.340 / 0.876 |
| *S. variabile* vs. *S. zapotecum* | 39.42 / 26.14 (65.56%) | 0.028 | 1.000 | 0.513 / 0.623 |

**Fig. S1** Mean mobility-oriented parity (MOP) test to evaluate the existence of areas of strict extrapolation of the three past climatic scenarios. The analysis was performed considering random sampling of 50% of the M surface. Similarity values to M for MOP calculations for the Mid Holocene and Last Glacial Maximum were obtained considering the average from CCSM4, MIROC-ESM, and MPI-ESM-P global climate circular models. Green polygons correspond to species’ potential distribution areas predicted for each temporal scenario; while the red polygons represent the individual “M” area defined for each species into modelling approach. The color scale indicates values of strict extrapolation (closer to 0) and similar to present (closer to 1).


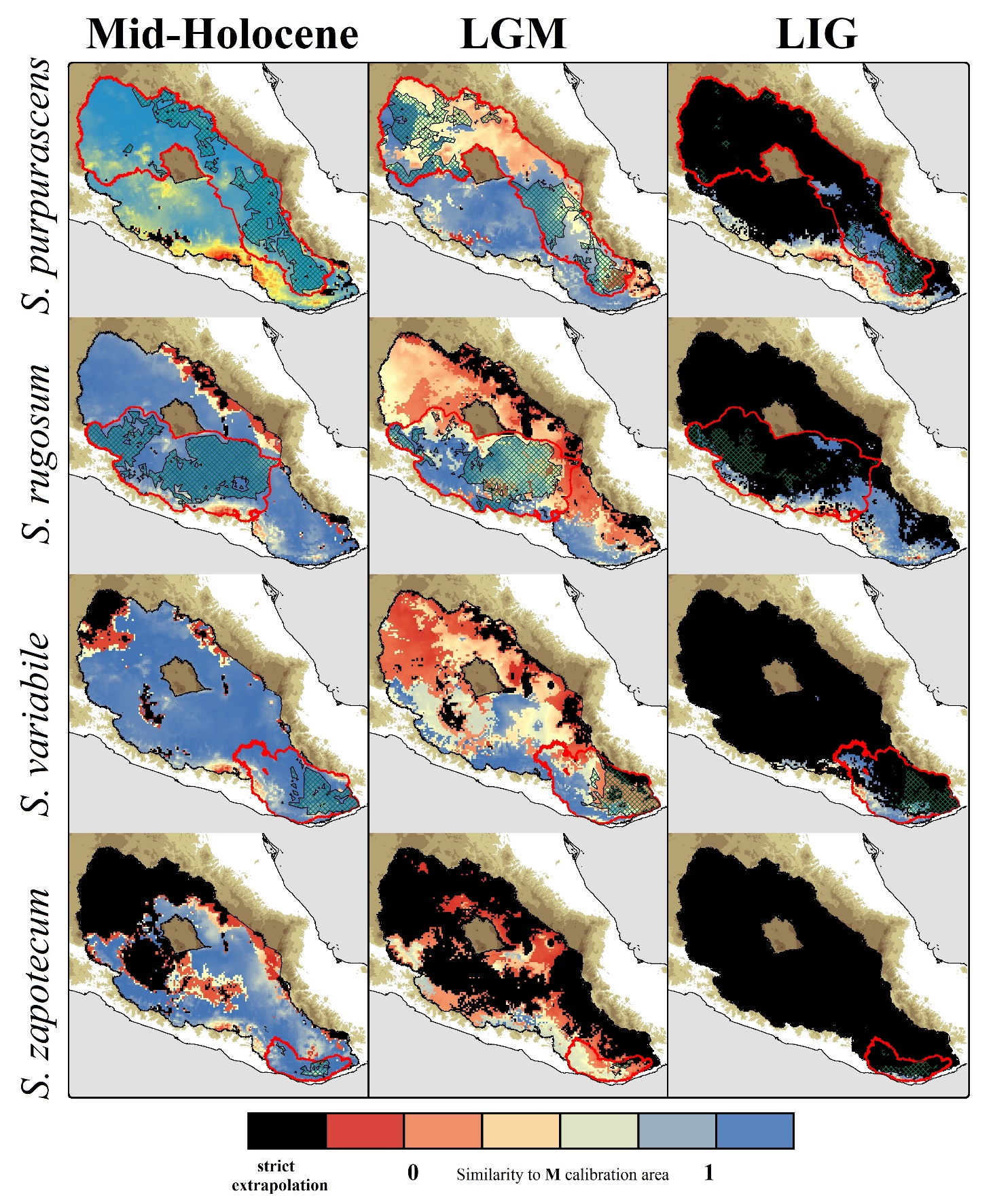

Supplement: Supplementary file 1 — Data S1: ece372209‐sup‐0001‐Supinfo.docx. [file ECE3-15-e72209-s001.docx]
